# Bretschneider solution-induced alterations in the urine metabolome in cardiac surgery patients

**DOI:** 10.1038/s41598-018-35631-w

**Published:** 2018-12-11

**Authors:** Cheng-Chia Lee, Ya-Ju Hsieh, Shao-Wei Chen, Shu-Hsuan Fu, Chia-Wei Hsu, Chih-Ching Wu, Wei Han, Yunong Li, Tao Huan, Yu-Sun Chang, Jau-Song Yu, Liang Li, Chih-Hsiang Chang, Yi-Ting Chen

**Affiliations:** 1 Kidney Research Center, Department of Nephrology, Chang Gung Memorial Hospital, Linkou branch, College of Medicine, Chang Gung University, Guishan, Taoyuan Taiwan; 2 Molecular and Medicine Research Center, Chang Gung University, Guishan, Taoyuan Taiwan; 3 Graduate Institute of Clinical Medical Sciences, College of medicine, Chang Gung University, Guishan, Taoyuan Taiwan; 4 Department of cardiothoracic and vascular surgery, Chang Gung Memorial Hospital, Linkou branch, College of Medicine, Chang Gung University, Guishan, Taoyuan Taiwan; 5 Department of Medical Biotechnology and Laboratory Science, Chang Gung University, Guishan, Taoyuan 33302 Taiwan; 6 Department of Otolaryngology-Head & Neck Surgery, Chang Gung Memorial Hospital at Linkou, Guishan, Taoyuan 33305 Taiwan; 7 Department of Chemistry, University of Alberta, Edmonton, AB T6G2G2 Canada; 8 Graduate Institute of Biomedical Sciences, Chang Gung University, Guishan, Taoyuan 33302 Taiwan; 9 Liver Research Center, Chang Gung Memorial Hospital at Linkou, Guishan, Taoyuan 33305 Taiwan; 10 Department of Cell and Molecular Biology, Chang Gung University, Guishan, Taoyuan 33302 Taiwan; 11 Department of Biomedical Sciences, College of Medicine, Chang Gung University, Guishan, Taoyuan Taiwan; 12 Graduate Institute of Biomedical Sciences, College of Medicine, Chang Gung University, Guishan, Taoyuan Taiwan

## Abstract

The development of Bretschneider’s histidine-tryptophan-ketoglutarate (HTK) cardioplegia solution represented a major advancement in cardiac surgery, offering significant myocardial protection. However, metabolic changes induced by this additive in the whole body have not been systematically investigated. Using an untargeted mass spectrometry-based method to deeply explore the urine metabolome, we sought to provide a holistic and systematic view of metabolic perturbations occurred in patients receiving HTK. Prospective urine samples were collected from 100 patients who had undergone cardiac surgery, and metabolomic changes were profiled using a high-performance chemical isotope labeling liquid chromatography-mass spectrometry (LC-MS) method. A total of 14,642 peak pairs or metabolites were quantified using differential ^13^C-/^12^C-dansyl labeling LC-MS, which targets the amine/phenol submetabolome from urine specimens. We identified 223 metabolites that showed significant concentration change (fold change > 5) and assembled several potential metabolic pathway maps derived from these dysregulated metabolites. Our data indicated upregulated histidine metabolism with subsequently increased glutamine/glutamate metabolism, altered purine and pyrimidine metabolism, and enhanced vitamin B_6_ metabolism in patients receiving HTK. Our findings provide solid evidence that HTK solution causes significant perturbations in several metabolic pathways and establish a basis for further study of key mechanisms underlying its organ-protective or potential harmful effects.

## Introduction

More than one million patients worldwide undergo cardiac surgery annually^1^. The development of cardiopulmonary bypass (CPB) in the 1950s and subsequent introduction of cardioplegia solutions were two tremendous leaps in the progression of cardiac surgery that allowed surgeons to repair intra-cardiac lesions with a bloodless field^2^. Although these two developments collectively reduced the risk of death from as high as nearly 50% in 1950s to single digit percentages today^2,3^, post-operative morbidity remains high. About 10–40% of cardiac surgeries will be complicated by peri-operative myocardial injury or infarction, leading to low cardiac output syndrome^4^. Acute kidney injury is another common complication following cardiac surgery, reported in up to 30–50% of patients in different studies^5,6^.

Possible mechanisms for these organ injuries after cardiac surgery include organ ischemia, subsequent reperfusion injury, and a systemic inflammatory response related to exposure to CPB per se or surgical trauma^7^. Moreover, the traditional hyperkalemia cardioplegic solution has been shown to exacerbate myocardial ischemia-reperfusion injury owing to sodium and calcium loading^3^. By contrast, Bretschneider’s Histidine-Tryptophan-Ketoglutarate (HTK) cardioplegic solution, the so-called intracellular solution, has low sodium and calcium and relatively low potassium, and has been extensively used clinically during cardiac surgery^8–10^. Some clinical reports have indicated that the use of HTK solution is associated with increased cardiac output and fewer arrhythmias than traditional hyperkalemic cardioplegic solution, suggesting better myocardial protection^9,11,12^, although others have reported equivalent myocardial protection and peri-operative complications for the two solutions^10,13^. Nonetheless, whereas cardiac effects of HTK solution during cardiac surgery have been studied extensively, metabolic alterations in other vital organs or in the whole body caused by these solutions have not been systematically investigated.

Metabolomics offers the ability to simultaneously detect many substrates, intermediates and products of metabolism, providing a holistic and systematic view of host responses to pathological status. Some metabolites may contribute to biological processes underlying organ hypoperfusion and possibly to post-cardiac surgery organ adaptation. To date, only a few metabolomic studies using ^1^H nuclear magnetic resonance or targeted mass spectrometry (MS)-based methods have been reported in samples of patients undergoing cardiac surgery^14–17^. However, no previous studies have used a sensitive untargeted MS-based technique to characterize the metabolic changes among these patients. In this study, we report the study of a deep and quantitative analysis of the untargeted urinary metabolite profiles of adult patients undergoing cardiac surgery using differential ^12^C-/^13^C-isotope dansylation labeling liquid chromatography (LC)-MS. The purpose of this investigation was to explore the metabolic shifts that occur with cardiac surgery, with a specific focus on the influence of HTK solution.

## Results

### Patient demographics and clinical characteristics

A total of 100 consecutive cardiac surgery patients, who provided informed consent, were enrolled in the current study. Clinical characteristics and surgical details of all study patients are summarized in Table 1. The mean age of the study population was 61.5 ± 13.6 years, and 61% were male. A history of diabetes mellitus was present in 40% of the cohort. Coronary artery bypass grafting was the most frequent surgery type (49%), followed by valve surgery (32%) and aortic surgery (16%). Of the 100 patients, 49 received HTK cardioplegia solution.

**Table 1:** Baseline characteristics of the study population.

Characteristics	All (n = 100)	PCA group 1 (n = 49)	PCA group 2 (n = 51)	*P* value
Age (years)	61.5 ± 13.6	58.9 ± 13.7	64.6 ± 12.6	0.030
Men [n (%)]	61 (61.0%)	21 (42.9%)	40 (78.4%)	<0.001
Diabetes mellitus [n (%)]	40 (40.0%)	12 (24.5%)	28 (54.9%)	0.002
Body mass index	24.7 ± 3.6	24.7 ± 3.6	24.7 ± 3.6	0.492
Ejection fraction (%)	59.5 ± 16.4	62.3 ± 15.8	56.9 ± 16.7	0.132
Preoperative Creatinine (mg/dl)	1.10 ± 0.59	0.99 ± 0.55	1.19 ± 0.63	0.097
Preoperative eGFR (ml/min)	75.9 ± 29.1	80.4 ± 30.1	71.6 ± 27.8	0.136
Surgery type [n (%)]				<0.001
CABG	37 (37.0%)	2 (4.1%)	35 (68.6%)	
Valve	32 (32.0%)	28 (57.1%)	4 (7.8%)	
CABG + Valve	12 (12.0%)	5 (10.2%)	7 (13.7%)	
Aorta	16 (16.0%)	12 (24.5%)	4 (7.8%)	
Other	3 (3.0%)	2 (4.1%)	1 (2.0%)	
Status of the procedure [n (%)]				0.722
Elective	76 (76.0%)	38 (77.6%)	38 (74.5%)	
Emergent	24 (24.0%)	11 (22.4%)	13 (25.5%)	
Cardioplegia solution HTK use [n (%)]	49 (49.0%)	49 (100%)	0 (0%)	<0.001

Note: Data are presented as mean ± SD and n (%). eGFR was calculated using Chronic Kidney Disease-Epidemiology Collaboration (CKD-EPI) equation.

Abbreviation: PCA, principal component analysis; eGFR, estimated glomerular filtration rate; CABG, coronary artery bypass grafting; HTK, Bretschneider’s histidine-tryptophan-ketoglutarate solution.

### Metabolomic analysis of urine using chemical isotope labeling (CIL) LC-MS

**Figure 1:**
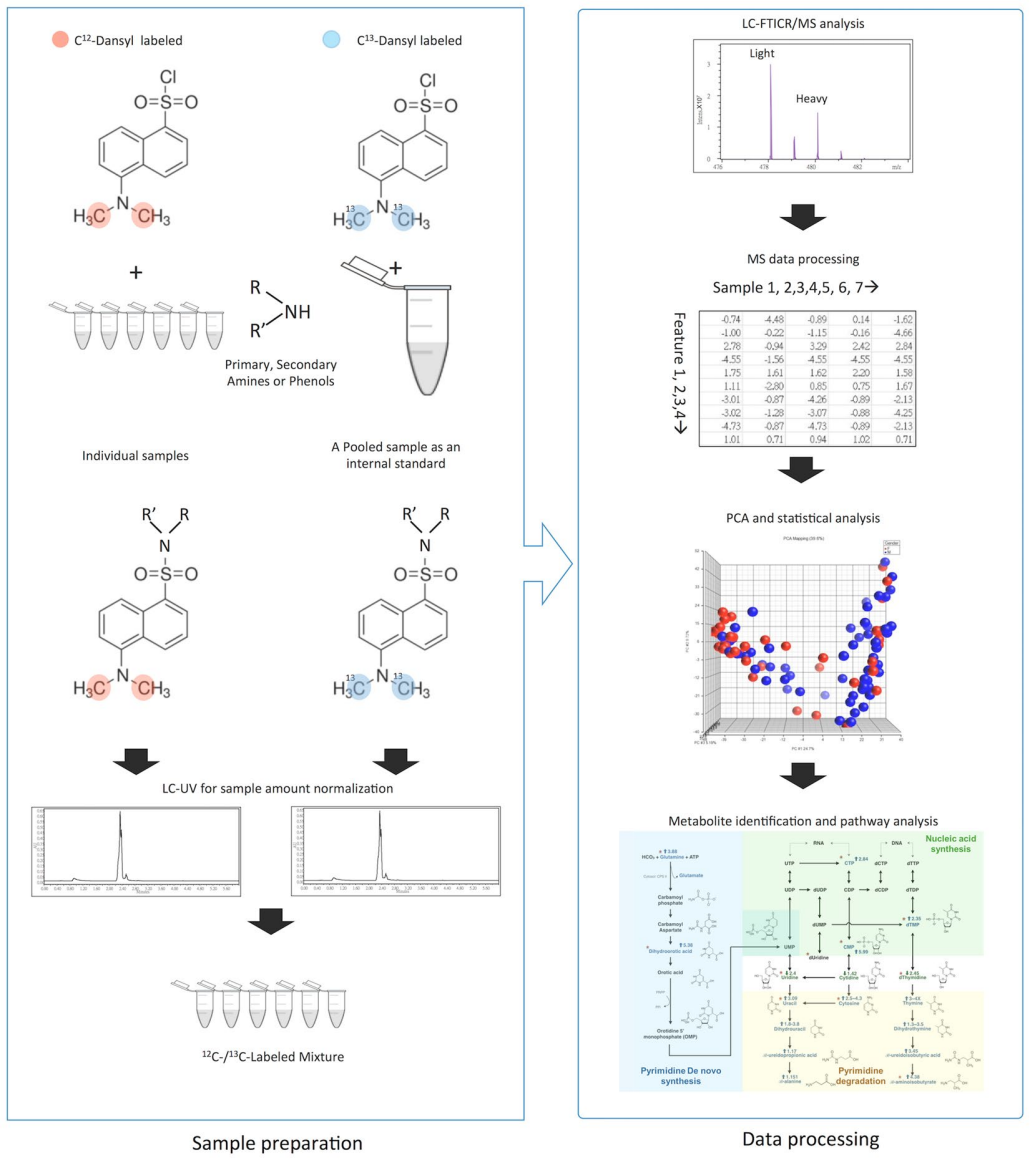
Workflow of the stable isotope labeled universal internal standard platform for urine metabolomics profiling.

Guo, K. demonstrated the excellent experimental reproducibility of dansylation method in urine samples and the experimental RSD ranges from 3.1 to 7% with an average of 5.3%^18^. We also tried to analyze pooled urine samples from 10 normal people which were labeled both heavy and light and equally mixed. The samples were labeled twice independently and there were more than 1426 peak pairs (80%) observed in both experiments. The ratio of 1426 peak pairs between two experiments were averaged with a relative standard deviation (RSD) as 4.99%. As the result showed, there were 57 peak pairs with quantitative ratio more than ±1.5-fold which implied the false determined rate was 4%. All the results above demonstrated that the dansylation platform was quite stable with excellent experimental reproducibility. Figure 1 shows the overall workflow of chemical isotopic dansylation labeling LC-MS for submetabolomic profiling of amine- and phenol-containing urinary metabolites in patients undergoing cardiac surgery. Amine- and phenol-containing metabolites are major groups of metabolites in the human metabolome involved in most known metabolic pathways. After processing, a total of 14,642 peak pairs were detected from the 100 urine samples with an average number of 5667 ± 638 per sample. A peak pair corresponds to the signal generated by an endogenous metabolite, as the IsoMS program filters out all the redundant peaks (adducts, dimers, etc.) from the same metabolite. A total of 2114 peak pairs or metabolites were detected in common in more than 80% of samples (Supplementary Fig. S1). By comparison, a previous study of the urine metabolome using dansylation LC-MS detected about 3000 peak pairs in each sample^19^. The greater number of metabolites detected in our samples indicates a higher sensitivity of our workflow and may also imply a more diverse urine metabolomic profile in cardiac surgery patients. Furthermore, a wide range of fold changes (^12^C-labeled individual sample vs. the same ^13^C-labeled pool) of metabolites from 0.04 to 400 was found, providing further evidence of the dramatic perturbations in physiological balance during cardiac surgery.

### Metabolomic profiles in patients undergoing cardiac surgery

**Figure 2:**
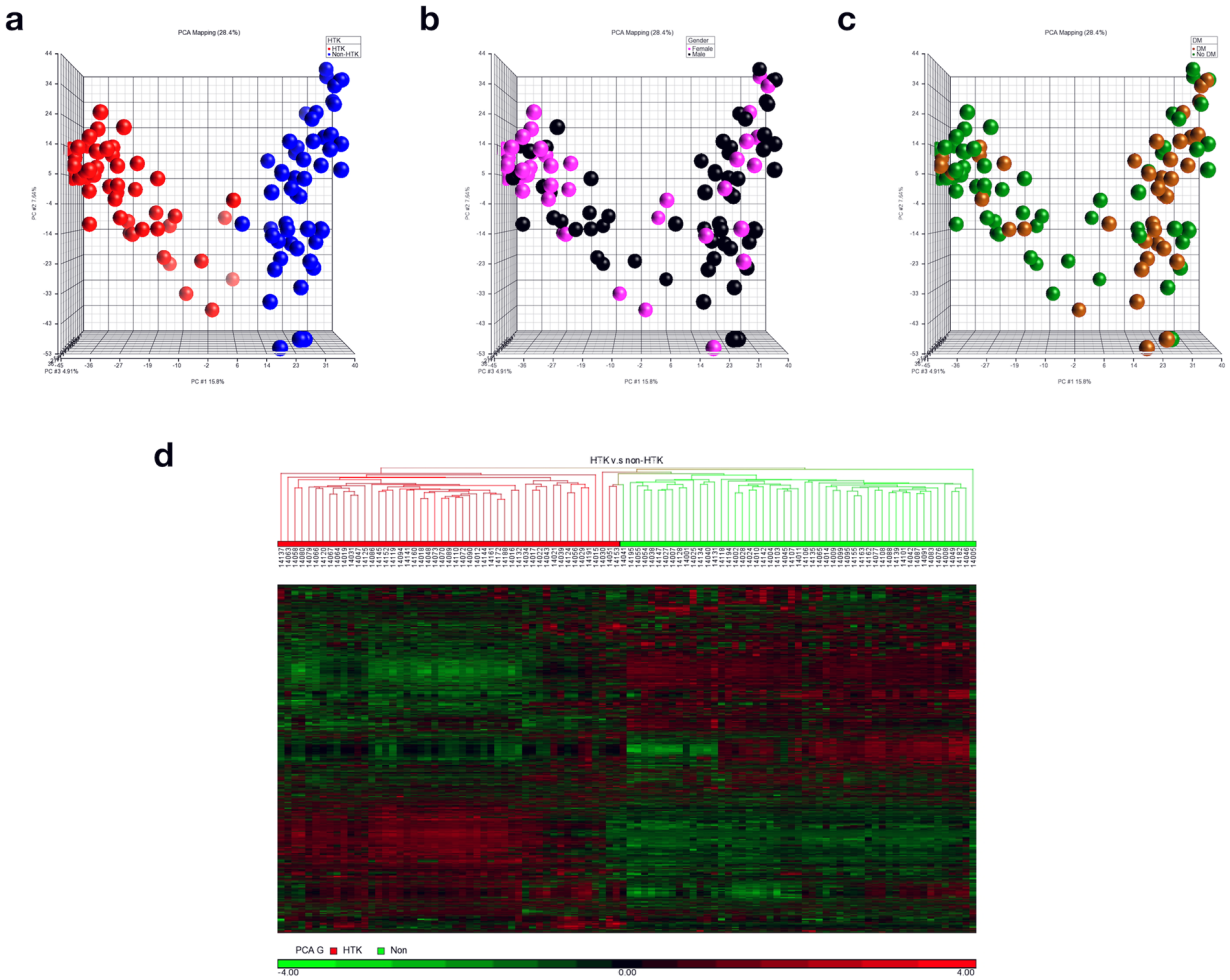
PCA and hierarchical clustering analyses of metabolomics data. The concentration ratios of all metabolite peak-pairs for the 100 urine samples were analyzed by PCA and hierarchical clustering. Results segregated on the basis of (**a**) HTK group (red) versus non-HTK group (blue) (**b**) biological sex (pink, females; black, males), and (**c**) DM (gold) versus non-DM (green). (**d**) Heatmap of hierarchical clustering of 7136 metabolite peak-pairs (HTK vs. non-HTK group). Each column represents an individual, and each row represents a metabolite. The color scale is log2 transformed value and indicates relative high (red) and low (green) metabolite levels.

To investigate the alteration of amine- and phenol-containing submetabolome among patients who had undergone cardiac surgery, we used a total of 14,642 concentration ratios of metabolite peak pairs from all 100 urine samples in principal component analysis (PCA). This analysis demonstrated two characteristic clustering (PCA group 1 versus PCA group 2) and we then discovered that the use of HTK during cardiac surgery clearly contributed to this segregation (Fig. 2a). As shown in Table 1, we observed that there were significant differences between PCA group 1 (HTK users) and PCA group 2 (non-HTK users) according to sex, the presence of diabetes, and surgical type. However, PCA showed that the measured urine submetabolome during the first 4 hour post cardiac surgery were not significantly different between the two groups with respect to sex (Fig. 2b), diabetes (Fig. 2c), surgical types, and even baseline renal function. We speculated the reasons are isotopic dansylation labeling mainly focused on amine- and phenol-containing metabolites, sugar-derived or gender-derived urinary metabolites could be missed in our study. Additionally, gender, diabetes differences maybe also missed by only the first three PCs are considered in this PCA analysis.

To study the representative metabolic shifts among patients, we included only those metabolite peak pairs detected in more than 50% of individual samples in each clinical group for statistical analysis. Applying these criteria, we obtained a dataset of 7136 metabolite peak pairs that was subsequently used for a multivariate analysis. The heatmap of these 7136 metabolites exhibited a distinctly different pattern between the two groups stratified by the use of HTK solution, indicating that the metabolomic changes in urine were caused by the use of HTK solution (Fig. 2d). We therefore performed a global assessment of urinary metabolites associated with HTK solution use.

### Targeted metabolic profiling related to the use of HTK cardioplegia solution

**Figure 3:**
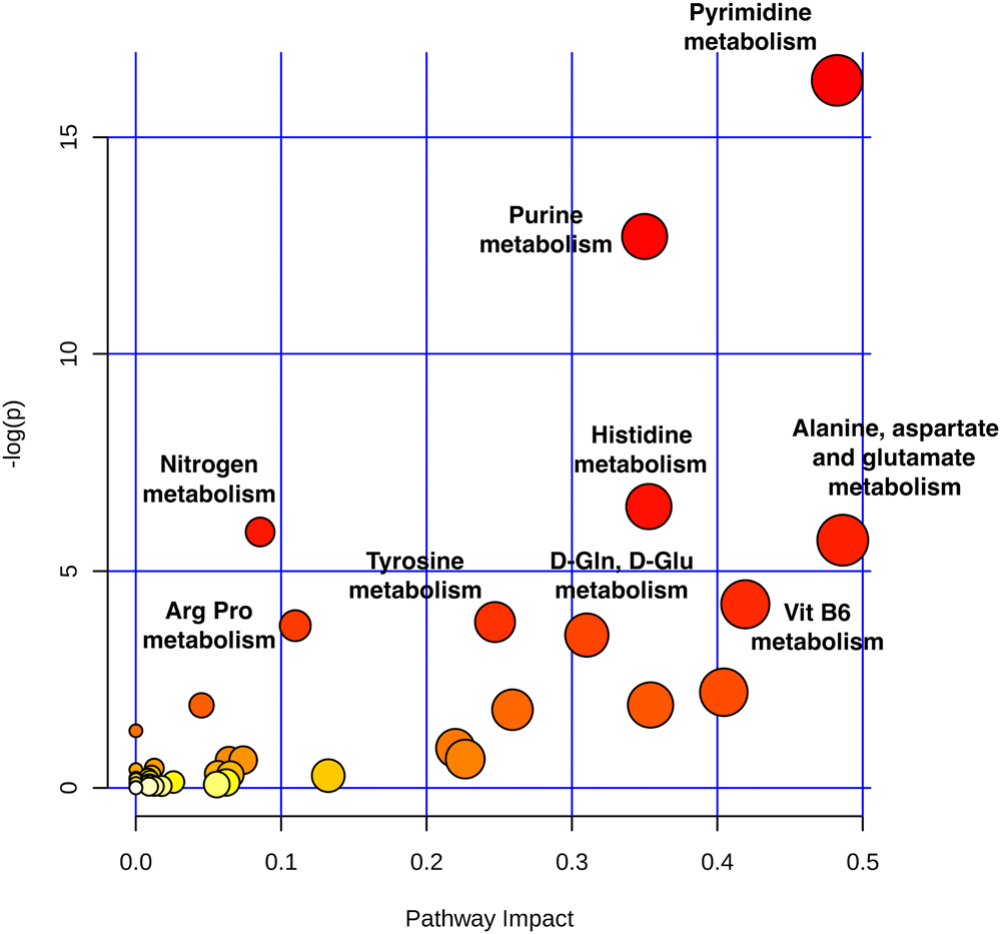
Metabolic pathway analysis plot created using MetaboAnalyst 3.0. Plots depict several metabolic pathway alterations induced by the use of HTK. The *x*-axis represents the pathway impact value computed from pathway topological analysis, and the *y*-axis is the -log of the *P*-value obtained from pathway enrichment analysis. The pathways that were most significantly changed are characterized by both a high -log(*p*) value and high impact value (top right region).

To determine pathways impacted by the use of HTK solution, we searched metabolite peak pairs with a significant change (*P* < 0.01) between the two groups of greater than 5-fold (log_2_ fold changes > 2.32) using MyCompoundID MS putative metabolite identification software^20^. 4816 metabolite peak pairs with the nominal significance by a *t*-test (*P* < 0.01) also passed the false discovery rate threshold (5%) accounting for multiple comparison^21^. As shown in Supplementary Table S1, 1567 significant metabolite peak pairs were differentially expressed between the two groups (Supplementary Dataset 1 and Supplementary Fig. S3), yielding 562 hits after a database search. Because dansylation only labels amine and phenol functional groups, we manually checked the structures of 562 hits and removed those without labelable amine or phenol functional groups. We also removed the redundant metabolites from the same m/z and retention time. A final dataset of 223 metabolite hits (Supplementary Dataset 2) was assessed using the web-based software, MetaboAnalyst 3.0, to identify affected metabolic pathways. This analysis revealed nine metabolic pathways (Fig. 3 and Supplementary Table S2) that were significantly altered by the use of HTK (*P* < 0.05). The four most significantly altered pathways were pyrimidine metabolism (impact value: 0.48, *P* < 0.001), purine metabolism (impact value: 0.35, *P* < 0.001), histidine metabolism (impact value: 0.35, *P* = 0.002), and alanine, aspartate, and glutamate metabolism (impact value: 0.49, *P* = 0.003).

**Figure 4:**
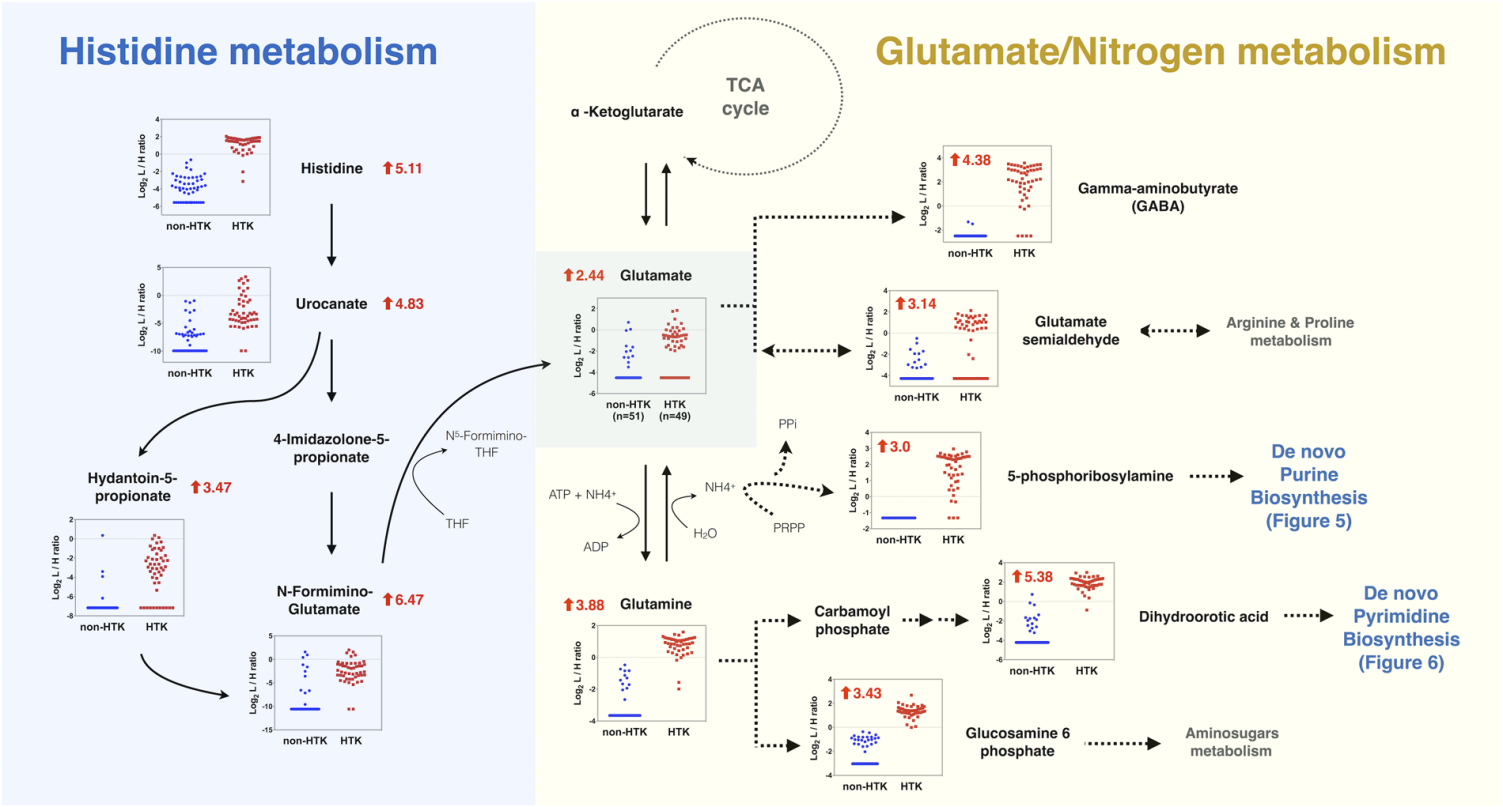
Integrated schematic illustration of metabolic alterations in histidine and glutamate/nitrogen metabolism induced by HTK. The scatter plots indicate the differential log_2_-concentration ratios of selected metabolites in the non-HTK group (blue, n = 51) versus the HTK group (red, n = 49). Each data point represents a single individual. Log_2_ fold changes are denoted as arrows and values (HTK/Non-HTK). Red indicates metabolites that were increased in the HTK group and black indicates metabolites that were decreased in the HTK group.

**Figure 5:**
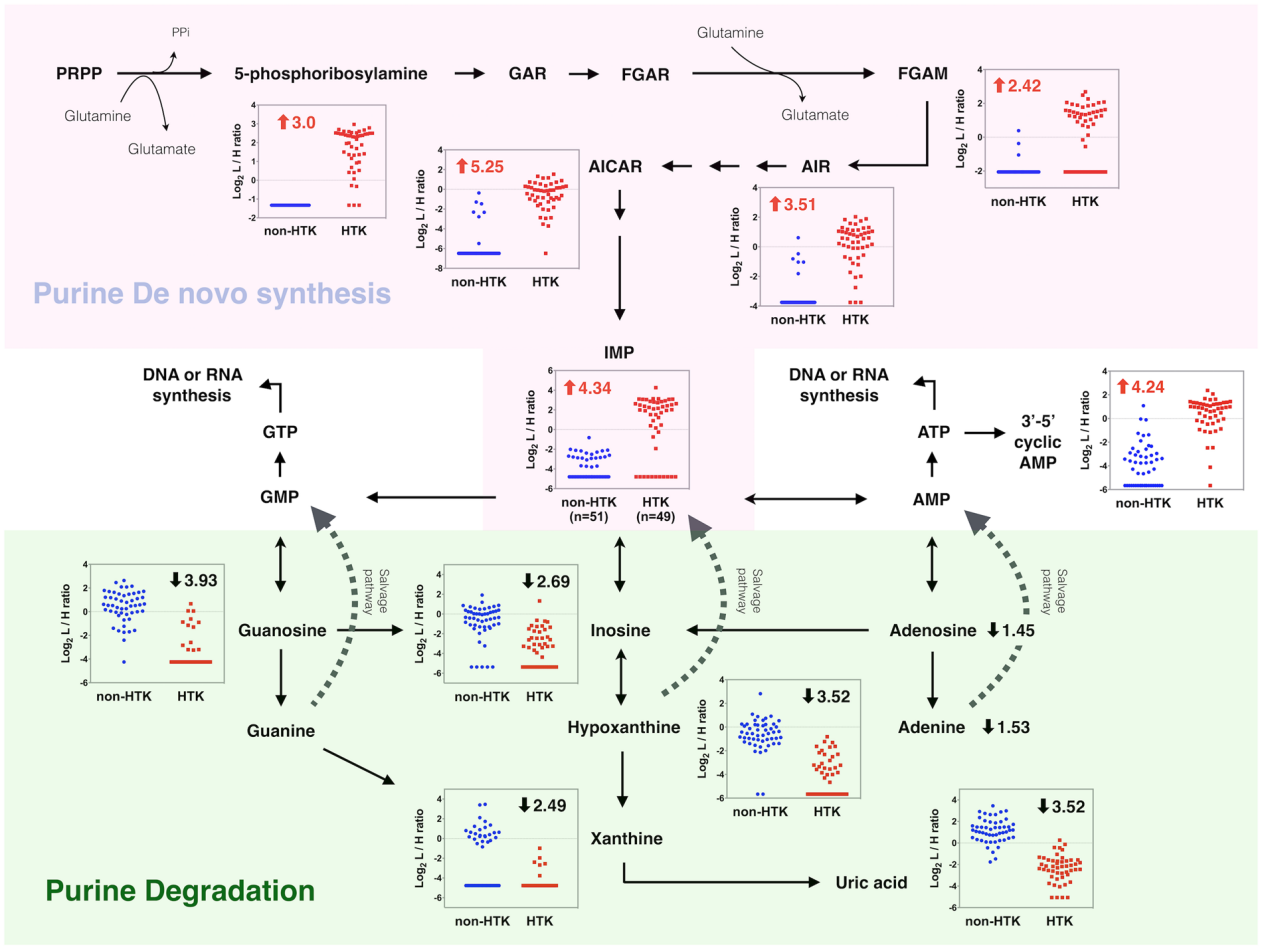
Integrated schematic illustration of metabolic alterations in purine metabolism induced by HTK. The scatter plots indicate the differential log_2_-concentration ratios of selected metabolites in the non-HTK group (blue, n = 51) versus the HTK group (red, n = 49). Each data point represents a single individual. Log_2_ fold changes are denoted as arrows and values (HTK/Non-HTK). Red indicates metabolites that were increased in the HTK group and black indicates metabolites that were decreased in the HTK group. Abbreviations: Phosphoribosyl pyrophosphate (PRPP), Glycineamide ribonucleotide (GAR), Formylglycinamide ribonucleotide (FGAR), Formylglycinamidine ribonucleotide (FGAM), Aminoimidazole ribonucleotide (AIR), Aminoimidazole carboxamide ribonucleotide (AICAR), Inosine monophosphate (IMP), Adenosine monophosphate (AMP), Adenosine triphosphate (ATP), Guanosine monophosphate (GMP), Guanosine triphosphate (GTP).

**Figure 6:**
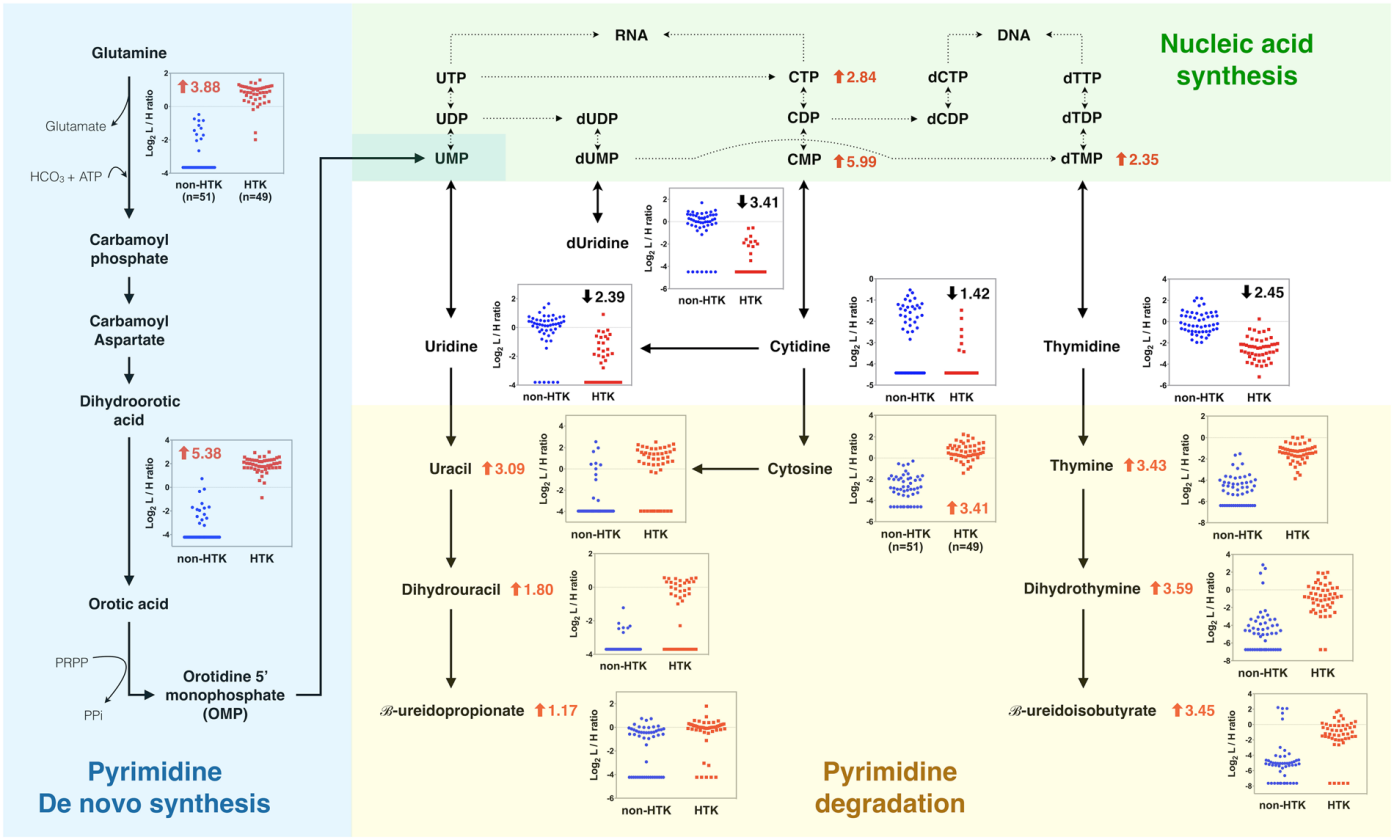
Integrated schematic illustration of metabolic alterations in pyrimidine metabolism induced by HTK. The scatter plots indicate the differential log_2_-concentration ratios of selected metabolites in the non-HTK group (blue, n = 51) versus the HTK group (red, n = 49). Each data point represents a single individual. Log_2_ fold changes are denoted as arrows and values (HTK/Non-HTK). Red indicates metabolites that were increased in the HTK group and black indicates metabolites that were decreased in the HTK group. Abbreviations: Uridine monophosphate (UMP), Uridine diphosphate (UDP), Uridine triphosphate (UTP), Cytidine monophosphate (CMP), Cytidine diphosphate (CDP), Cytidine triphosphate (CTP), deoxythymidine monophosphate (dTMP), deoxythymidine diphosphate (dTDP), deoxythymidine triphosphate (dTTP).

Further analysis of each metabolite in the nine significant pathways showed that several overlapping metabolites behaved as hub metabolites that were capable of linking the various pathways. Using two such metabolites, glutamine and glutamate, we were able to construct a combined pathway map (Fig. 4) that involved five identified pathways: histidine metabolism, nitrogen metabolism, alanine, aspartate and glutamate metabolism, arginine and proline metabolism, and glutamine and glutamate metabolism. The same approach was also used to extend this analysis to consider the complex crosstalk between this pathway map and two of the remaining altered pathways identified in this study: purine metabolism (Fig. 5) and pyrimidine metabolism (Fig. 6).

Taking into account fold-changes (expressed on a log_2_ scale) in the involved metabolites, determined by differential ^12^C-/^13^C-isotope labeling, we investigated how alterations in metabolites during cardiac surgery were modulated by HTK solution. Figure 4 shows that histidine and the principal degradation products of histidine were distinctly increased after the use of HTK solution, indicating an increase in histidine metabolism. In addition to higher levels of glutamate among HTK users, we also observed an almost 15-fold increase in glutamine compared with that among non-HTK users. As shown in Figs 4 and 5, 5-phosphoribosylamine and some downstream derivatives involved in de novo purine biosynthesis were consistently increased among HTK users, whereas most metabolites associated with purine-degrading metabolic processes were significantly and consistently decreased. We also found that 1-methyladenosine was significantly decreased by ~4-fold among HTK users (Supplementary Fig. S2a). As shown in Figs 4 and 6, dihydroorotic acid and some nucleic acids showed an increase among HTK users, suggesting increased pyrimidine de novo synthesis and nucleic acid synthesis after HTK use. The four pyrimidine ribonucleosides all showed the same decreasing trend in response to HTK use. In contrast, free pyrimidine bases (cytosine, uracil, thymine) and their downstream products (e.g., dihydrouracil, dihydrothymine) involved in the pyrimidine-degradation pathway all significantly increased (by 2- to 10-fold) in response to the use of HTK solution.

## Discussion

One of the challenges of metabolomic profiling analysis is the diverse hydrophilicity of chemical structures and poor ionization efficiency of metabolites during ionization by reversed-phase (RP) LC-MS, the most common LC system for MS. Here, we labeled metabolites with dansyl chloride, which increases the hydrophobicity to an extent that the labeled metabolites can be efficiently retained and separated in RPLC and enhances ionization efficiency by 1–3 orders of magnitude^18^, enabling us to systematically investigate hydrophilic metabolites as well as hydrophobic metabolites. In addition, the isotopic peak-pairs allowed us to distinguish background noise from metabolite signals, since redundant peaks such as sodium or potassium adducts, tailing peaks, isotopic peaks, and different charged peaks could be considered for grouping and removal. Furthermore, our study showed that the peak pair number increased from 1118 to 5667 per sample after using the Zerofill and IsoMS-Quant Program to retrieve missing values, mostly metabolites with low intensities; thus, we were able to obtain a more comprehensive profile of the urine metabolome of cardiac surgery patients. Compared with a label-free strategy, which yields a total of about 15,000 metabolite signals or features and most of them are redundant and noise peaks^22,23^, our platform offers a number of advantages. First, our method was capable of detecting all hydrophilic compounds, which are difficult detect in label-free systems owing to poor binding or the weaker power of RPLC to separate hydrophilic compounds. Second, we applied a universal pooled standard (heavy label) in every sample as a reference or control, resulting in more accurate quantification than is possible with a label-free system, where comparisons and normalizations between runs are always a problem. Thus, the improved ability of untargeted dansylation metabolomics allowed us to identify more metabolites within a given pathway and further strengthen confidence in the significant biological processes or drug metabolism identified in this patient group.

Although HTK cardioplegia solution has demonstrated promise in supporting extended periods of myocardial ischemia during cardiac surgery, relatively little is known about how the human body modulates its metabolites and their interconversions, which may potentially contribute to other systematic effects. Critically, some studies have shown that HTK solution is associated with reduced graft survival when used for kidney^24^ or liver^25^ preservation during transplantation in cases of extended-criteria donors or organs donated after cardiac death, implying that HTK or its metabolites might be harmful to donor grafts with prolonged cold ischemia. The current study using untargeted metabolomics to address metabolic shifts in patients receiving HTK solution helps to establish a foundation for further investigation into key mechanisms by which HTK solution contributes to organ protection or causes potential harm.

Among the metabolomic alterations identified, the most prominent were related to histidine metabolism. It is conceivable that HTK solution, which contains 198 mM histidine, resulted in an elevated histidine concentration in plasma with subsequently increased histidine degradation. Although data regarding amino acid turnover during cardiac surgery is limited, it has been reported that surplus histidine cannot be stored and may be catabolized. Relevant in this context is an earlier report by Teloh *et al*. that administration of HTK solution during cardiac surgery leads to marked elevations in histidine, glutamate, and glutamine concentration in both plasma and urine^26^. Our data (Fig. 4) provide further evidence showing consistent increases in the levels of downstream degradation metabolites of histidine, including urocanate, hydantoin-5-propionate, N-formimino-glutamate and, ultimately, glutamate.

Subsequent pathway analyses revealed that two metabolites, glutamate and glutamine, occupied the nexus of five of nine pathways that showed significant HTK-induced alterations. Glutamate and glutamine play crucial roles in amino acid metabolism. In adults in positive nitrogen balance, amino acids in the liver first lose their amine group through transamination to form glutamate, which enters mitochondria and is metabolized to ammonia and ultimately converted to urea for excretion. However, most amino acid degradation takes place in extrahepatic tissues, which lack the enzymes of the urea cycle. Thus, excess ammonia in these tissues must combine with glutamate to yield glutamine first before transport to the liver^27^. In line with this, our study showed that the level of glutamine in the HTK group was significantly increased. Critics may argue that the increased level of glutamine could have resulted from more muscle protein breakdown in patients receiving HTK compared with those without HTK. However, our analysis refutes this idea, showing that urinary 3-methylhistidine level, an index of muscle protein catabolism^28^, was significantly reduced in the HTK group, with a log_2_ fold-change of −2.2 (Supplementary Fig. S2b). Nevertheless, transamination to glutamate and other amino acids through transaminase-catalyzed reactions requires pyridoxal-5′-phosphate, a derivative of vitamin B_6_, as a cofactor. Intriguingly, our data demonstrated that vitamin B_6_ metabolism is one of the three most-significantly altered pathways in patients receiving HTK solution, showing that urinary 4-pyridoxic acid, which is the major catabolite of vitamin B_6_^29,30^, was significantly reduced. In addition, our data revealed that pyridoxal 5′-phosphate, the metabolically active form of vitamin B_6_, was significantly increased among HTK users, indicating a vitamin B_6_ status sufficient for the process of degrading surplus amino acids.

Glutamine has also been considered a conditionally essential amino acid in certain catabolic states because its utilization exceeds its de novo synthesis during critical illnesses. A recent report showed that glutamine levels are significantly lower immediately after cardiac surgery compared with pre-operative levels^31^. In addition, several clinical studies have demonstrated associations between low plasma glutamine levels and unfavorable outcome in critically ill patients^32,33^. However, clinical trials regarding glutamine supplementation and reduced mortality have yielded seemingly inconsistent results^34,35^. Our data showed that the level of glutamine was increased in the HTK group. Whether this increased synthesis of glutamine from HTK solution confers clinical benefit or potential harm will require further investigation.

Glutamate/glutamine metabolism constitutes one of the major pathways in the biosynthesis of nucleic acids and proteins^36^. Using untargeted CIL LC-MS, we identified multiple intermediate metabolites that participated in de novo purine and pyrimidine biosynthesis and showed that they were consistently increased in the HTK group. Some studies have suggested a link between increased catabolism of purines and acute systemic hypoxia^37,38^. During ischemia, increased degradation of adenosine triphosphate and activity of xanthine oxidase may lead to the formation of xanthine and uric acid. In addition, purine nucleosides can be released from injured and hypoxic cells and remain elevated for days after the insult. An increase in urinary 1-methyladenosine was also reported to be a sensitive biomarker of cellular hypoxia^38^. Our study found that most purine catabolites, including 1-methyladenosine, decreased in the HTK group, although de novo purine biosynthesis was upregulated. Whether these findings suggest less systemic hypoxia in our HTK group warrants further study. On the other hand, the specific mechanism underlying the upregulation of de novo purine biosynthesis is not clear, but it is possible that the total purines pool might be depleted during cardiac surgery. Thus, the increase in glutamine synthesis caused by HTK solution might provide sufficient precursors for de novo purine synthesis in conjunction with a decrease in purine catabolism that restores purine levels to normal^39^.

In addition to altered purine metabolism, our data revealed that de novo pyrimidine and nucleic acid synthesis pathways were increased, as were products of pyrimidine degradation. A possible explanation for these observations is that HTK use leads to increased glutamine synthesis, which provides the fuel necessary to increase de novo pyrimidine synthesis^39^. A recent report demonstrated that reactive oxygen species (ROS) may increase endothelial injury and subsequent release of RNASE1 (ribonuclease A family 1), which then degrades extracellular ribonucleic acid and leads to an increase in purine and pyrimidine degradation products^40^. However, our study demonstrated that patients receiving HTK had higher levels of pyrimidine degradation products, but lower levels of purine degradation products. Thus, increased ROS is unlikely to have contributed appreciably to changes in the metabolome in patients receiving HTK.

There are several limitations of this study. First, the reaction characteristics of dansyl chloride labeling only allow detection of amine- and phenol-containing metabolites. We expect that integration of our data with submetabolomes obtained using three additional labeling methods would provide a more comprehensive view of the HTK urine metabolome^41^. Second, positive identification of metabolites in each pathway needs to be strengthened. It requires the extension of the entries in metabolite standard library and human metabolome databases. Third, although some of our results are consistent with previous findings of Teloh *et al*.^26^, patients receiving HTK or not might differ with variability in disease state coupled with individual differences in drug response. Thus, some metabolic alteration may have derived from non-HTK source. Fourth, the sample size in different groups is still limited. Future work of recruiting more patients based on sex and comorbidity will provide the opportunity to validate the findings presented herein.

In summary, untargeted metabolomics provides a powerful approach for detecting and quantifying metabolic shifts in patients receiving HTK cardioplegia solution in association with cardiac surgery. Although a medium-sized sample set was analyzed, this is the first study to utilize a global approach to address this issue. Metabolic pathway analysis revealed increased histidine metabolism with subsequently increased glutamine/glutamate metabolism, altered purine and pyrimidine metabolism, and increased vitamin B6 metabolism in patients receiving HTK. Critically, the ideal cardioplegic solution in cardiac surgery is still a matter of debate and the safety of using HTK solution in the setting of donor grafts with prolonged cold ischemia is still a concern. These metabolites and pathways may suggest likely mechanisms involved in the organ-protective or potential harmful effects of HTK solution. Future studies of the relationship between the metabolome and clinical disease will also need to closely examine the effects of medications on changes in metabolic profile.

## Methods

### Subject enrollment and clinical sample collection

This prospective study was carried out in accordance with the Declaration of Helsinki and was approved by the institutional review board of the Chang Gung Memorial Hospital (IRB number: 104–2821C). This study was performed in the cardiac surgery intensive care unit (ICU) at a tertiary care referral center in Taiwan between July 2014 and February 2015. During this period, anesthetic management for cardiac surgery was standardized and CPB was established after a standard median sternotomy, aortic root cannulation, and atrial cannulation for venous return. Myocardial protection was achieved using two types of cardioplegia solution either HTK solution or traditional hyperkalemia solution depending on the preference of the surgeon. Consecutive patients admitted to the ICU immediately after cardiac surgery were enrolled after providing written informed consent. Exclusion criteria included patients younger than 20 years of age; preexisting renal insufficiency, defined as a preoperative estimated glomerular filtration rate less than 30 ml/min; and anuria immediately after cardiac surgery. Fresh urine samples were obtained during the first 4 hours postoperatively via an indwelling Foley catheter and then centrifuged at 4000 rpm for 10 min at 4 °C to remove cells and debris. The supernatants were filtered twice through 0.22-μm filters (Millipore) and then stored at −80 °C until analysis.

### Dansylation labeling LC-MS metabolome profiling and data processing

Figure 1 shows the overall workflow for dansylation labeling LC-MS. The detailed chemical isotopic dansylation method is available in Supplementary Methods. A ^13^C-dansyl chloride-labeled, pooled urine sample, prepared using a mixture of individual urine samples, served as a universal internal standard. Separately, individual urine samples were labeled with ^12^C-dansyl chloride. Labeled samples were quantified using the LC-UV method^42^ to normalize the concentration of metabolites, after which light (^12^C-labeled individual urine specimen) and heavy (^13^C-labeled universal internal standard) samples were mixed in equal moles and metabolites were quantified using LC-MS analysis. This method of sample normalization makes it possible to acquire an accurate concentration ratio of a given metabolite in a fixed amount of total metabolites of all individual samples, even though the total metabolite concentration varies significantly from one sample to another. For LC-MS analysis, we used a Waters ACQUITY Ultra-high-pressure liquid chromatography system (Waters) connected to a Fourier Transform Ion-Cyclotron Resonance MS system (Apex-Qe-SHEDS FTICR, 9.4 Tesla; Bruker Daltonics, Bremen, Germany). The resulting MS data were then processed using a pipeline developed by Li’s group (http://www.mycompoundid.org), written in the R programming language. Details of MS methods and data processing are available in Supplementary Methods. Final identification of putative metabolites was based on accurate mass matches of metabolites in the HMDB (www.hmdb.ca) and MyCompoundID MS Search Program (www.mycompoundid.org) databases. Pathway analyses were performed using the online analysis tools, MetaboAnalyst^43^ (www.metaboanalyst.ca) and KEGG library (http://www.genome.jp/kegg/).

### Statistical analysis

Each urine sample was analyzed three times by MS, and peak-pairs were aligned based on retention time and accurate mass using IsoMS-align script^44^. The peak-pair ratio of each sample was globally normalized to the median of each run, and only those peak-pair features that showed a coefficient of variation (CV) <30% in triplicate clinical samples were retained and averaged for quantification. In order to facilitate visualization and perform statistical analysis, metabolite peak pairs detected in more than 50% of individual samples in each clinical group were retained for further analysis and 7136 peak pairs were selected. The metabolite intensity in MS spectra below the detection limit was assigned the value of half the minimum light-to-heavy ratio of the same metabolites in all urine specimens. The concentration ratios of all metabolite peak pairs were log_2_ transformed before further statistical analysis, such as fold change, *t*-test, and hierarchical clustering. PCA analysis was performed using fold changes (linear) and total 14642 peak pairs were analyzed. PCA and clustering were performed using Partek Genomics Suite software, version 6.6 (Partek Inc., St. Louis, MO, USA). Metabolite peak-pairs with fold changes ≥5.0 or ≤0.2 (log_2_ ratio > 2.32 or <−2.32) and a *P*-value ≤ 0.01 were considered statistically significant. With regard to multiple comparison, we also applied Benjamini-Hochberg method using false discovery rate at 5% as a threshold for statistically significant^21^.

## Electronic supplementary material

Supplementary information

Supplementary Dataset 2

Supplementary Dataset 1
