# Supplementary material for: Bretschneider solution-induced alterations in the urine metabolome in cardiac surgery patients
Source: Sci Rep. 2018 Dec 11;8:17774. doi: 10.1038/s41598-018-35631-w (PMC6290005; doi:10.1038/s41598-018-35631-w)

Bretschneider solution-induced alterations in the urine metabolome in cardiac surgery patients

Cheng-Chia Lee[1,3,#], Ya-Ju Hsieh[2,#], Shao-Wei Chen[3,4], Shu-Hsuan Fu[2], Chia-Wei Hsu[2], Chih-Ching Wu[2,5,6], Wei Han[7], Yunong Li[7], Huan Tao[7], Yu-Sun Chang[2,6,8], Jau-Song Yu[2,9,10], Liang Li[7,*], Chih-Hsiang Chang[1,3,*], Yi-Ting Chen[1,2,11,12,*]

[1]Kidney Research Center, Department of Nephrology, Chang Gung Memorial Hospital, Linkou branch, College of Medicine, Chang Gung University, Taoyuan, Taiwan

[2] Molecular and Medicine Research Center, Chang Gung University, Taoyuan, Taiwan

[3]Graduate Institute of Clinical Medical Sciences, College of medicine, Chang Gung University, Taoyuan, Taiwan

[4] Department of cardiothoracic and vascular surgery, Chang Gung Memorial Hospital, Linkou branch, College of Medicine, Chang Gung University, Taoyuan, Taiwan

[5]Department of Medical Biotechnology and Laboratory Science, Chang Gung University, Guishan, Taoyuan 33302, Taiwan

[6] Department of Otolaryngology-Head & Neck Surgery, Chang Gung Memorial Hospital at Linkou, Gueishan, Taoyuan 33305, Taiwan

[7] Department of Chemistry, University of Alberta, Edmonton, AB, T6G2G2, Canada

[8]Graduate Institute of Biomedical Sciences, Chang Gung University, Guishan, Taoyuan 33302, Taiwan

[9] Liver Research Center, Chang Gung Memorial Hospital at Linkou, Gueishan, Taoyuan 33305, Taiwan.

[10]Department of Cell and Molecular Biology, Chang Gung University, Guishan, Taoyuan 33302, Taiwan

[11]Department of Biomedical Sciences, College of Medicine, Chang Gung University, Taoyuan, Taiwan

[12] Graduate Institute of Biomedical Sciences, College of Medicine, Chang Gung University, Taoyuan, Taiwan

[#]Cheng-Chia Lee and Ya-Ju Hsieh contributed equally to this manuscript.

*Correspondence and requests for materials should be addressed to Yi-Ting Chen. (email: ytchen@mail.cgu.edu.tw) or Chih-Hsiang Chang (franwisandsun@gmail.com; sunchang@cgmh.org.tw) or Liang Li (email: liang.li@ualberta.ca) Tel: +886-32118800 ext. 3558; FAX: +886-3-211-8700.

I. Supplementary Methods -------------------------------------- Page 1 - 4

II. Supplementary References ------------------------------------ Page 5

III. Supplementary Table S1 and S2 ----------------------------- Page 6-7

IV. Supplementary Figure S1, S2, and S3 ---------------------- Page 8-10

V. Supplementary Figure S3-1 ~ S3-57 ------------------------- Page 11-67

I. Supplementary Methods

The strategy of dansylation-labeling LC-MS metabolome profiling using a universal internal standard

A chemical derivatization strategy based on the dansylation reaction was used for relative quantification of amine- and phenol-containing metabolites in a given sample. Individual samples were separately labeled with ^{12}C-dansyl chloride (light labeling), while a pooled sample prepared by mixing aliquots of individual samples was labeled with ^{13}C-dansyl chloride (heavy labeling). The ^{13}C-dansyl chloride-labeled, pooled sample served as a universal internal standard for all ^{12}C-dansyl chloride-labeled individual samples. The concentrations of dansyl-labeled metabolites in samples, quantified using an LC-UV method[1], were normalized, individual ^{12}C-labeled (light) and ^{13}C-labeled, pooled (heavy) samples were mixed equally. The metabolite mixtures were measured and quantified then using LC-MS analysis. This sample normalization method makes it possible to acquire accurate a concentration ratio of a given metabolite in a fixed amount of total metabolites of

all individual samples, even though the total metabolite concentration varies from one sample to another.

Reagents

Compounds for dansylation, including dansyl chloride, sodium bicarbonate ($NaHCO_3$), sodium carbonate (Na_2CO_3), sodium hydroxide (NaOH), formic acid and amino acid standards (AAS 18), were purchased from Sigma–Aldrich (St. Louis, MO, USA). Acetonitrile containing 0.1% formic acid, water containing 0.1% formic acid, MS grade water and acetonitrile, used for the LC-MS buffer system, were also obtained from Sigma–Aldrich. ^{13}C-dansyl chloride was custom-synthesized by Wuxi Beita Pharmatech (Wuxi, China).

Dansylation labeling

The dansylation method used is based on that reported by Guo et al [2]. In brief, a pooled sample, used as a universal internal standard, was prepared by combining aliquots from individual samples and mixing well. Thereafter, 25 μl of 0.5 M $NaHCO_3/Na_2CO_3$ and 75 μl of dansyl chloride (1 mg per 80.5 μl of acetonitrile; ^{12}C-dansyl chloride for individual samples and ^{13}C-dansyl chloride for pooled internal standard) was added into 50 μl samples (previously diluted in cases where the concentration of metabolites was high). Labeling reactions were performed by incubating at 40°C for 45 min on an orbital shaker (200 rpm) and then immediately placing on ice to stop the reaction. Excess dansyl chloride was quenched by adding 10 μl of 250 mM NaOH and incubating at 40°C for 10 min. After adding 50 μl of 425 mM formic acid/50% acetonitrile into samples, the concentration of metabolites was determined using a LC-UV method. Amino acid standards (AAS18; Sigma-Aldrich)

were serially diluted and labeled for use in preparing a standard curve for concentration normalization [1].

LC-UV quantitation

Metabolite concentration was determined by measuring absorbance at 338 nm using a Waters ACQUITY UPLC system (Waters, Milford, MA, USA) equipped with a TUV detector. Prepared samples (2 μl) were analyzed on a Waters ACQUITY BEH C_{18} column (2.1 × 50 mm, 1.7 μm particle size, 130 Å) using a flow rate of 0.45 ml/min. The column was eluted using a fast step gradient starting with 99% mobile phase A (0.1% [v/v] formic acid in 5% acetonitrile) and 1% mobile phase B (0.1% formic acid in acetonitrile) for 1 min, and increasing to 95% B within 0.01 min and holding for 1 min to elute all labeled metabolites. The gradient was returned to 1% B over 0.5 min and held at 1% for 3.5 min to re-equilibrate the column. A standard curve was prepared from an amino acid standard mixture (AAS18, Sigma–Aldrich) by serially diluting (8.3, 6.3, 4.2, 2.1, 1.0, 0.42, 0.1 and 0.02 mM), labeling with dansyl chloride, and detecting by measuring LC-UV absorption at 338 nm.

LC-MS analysis

For each LC-MS run, 10 nmol of dansyl-labeled amine-phenol metabolites was analyzed using a Fourier Transform Ion-Cyclotron Resonance mass spectrometer (Apex-Qe-SHEDS FTICR system, 9.4 Tesla; Bruker Daltonics, Bremen, Germany) linked to a Waters ACQUITY UPLC system (Waters). Samples were run on a reversed-phase Waters ACQUITY BEH C_{18} column (1 × 100 mm, 1.7 mm particle size, 130 Å) at a flow rate of 60 μl/min, and eluted with the following gradient of solvent A (0.1% [v/v] formic acid in 5% [v/v] acetonitrile) and B (0.1% [v/v] formic acid in acetonitrile): t = 0 min, 5% B; t = 2 min, 5% B; t = 3 min, 15% B; t = 13 min, 35% B; t = 25 min, 70% B; t = 28 min, 99% B; t = 30

min, 99% B; t = 30.1 min, 5% B; t = 32.5 min, 5% B. All MS spectra were obtained in positive ion mode.

Data processing

The resulting MS data were processed using a pipeline developed by Li's group (http://www.mycompoundid.org/mycompoundid_IsoMS/LCMS.jsp) and written in the R programming language. Briefly, raw data were transformed into peak pairs using IsoMS software and each peak pair was aligned using the IsoMS-align script [3]. Since this feature alignment is based on m/z and retention time, we also adapted the RT correction script to correct the minor shift in retention time [2]. Missing values were filled using the Zero-fill program, based on the signal of universal heavy dansyl-labeled standards in each individual sample [4]. The final metabolite-intensity data file was recalculated with IsoMS-Quant using chromatographic area information [5]. Visualization and statistical analyses were facilitated by assigning metabolite intensities in MS spectra below the detection limit a value of half the minimum light-to-heavy ratio of the same metabolites in all urine specimens.

II. Supplementary References

1 Wu, Y. & Li, L. Determination of total concentration of chemically labeled metabolites as a means of metabolome sample normalization and sample loading optimization in mass spectrometry-based metabolomics. *Anal Chem* **84**, 10723-10731, doi:10.1021/ac3025625 (2012).

2 Guo, K., Ji, C. & Li, L. Stable-isotope dimethylation labeling combined with LC-ESI MS for quantification of amine-containing metabolites in biological samples. *Anal Chem* **79**, 8631-8638, doi:10.1021/ac0704356 (2007).

3 Zhou, R., Tseng, C. L., Huan, T. & Li, L. IsoMS: automated processing of LC-MS data generated by a chemical isotope labeling metabolomics platform. *Anal Chem* **86**, 4675-4679, doi:10.1021/ac5009089 (2014).

4 Huan, T. & Li, L. Counting missing values in a metabolite-intensity data set for measuring the analytical performance of a metabolomics platform. *Anal Chem* **87**, 1306-1313, doi:10.1021/ac5039994 (2015).

5 Zhou, R. & Li, L. Effects of sample injection amount and time-of-flight mass spectrometric detection dynamic range on metabolome analysis by high-performance chemical isotope labeling LC-MS. *J Proteomics* **118**, 130-139, doi:10.1016/j.jprot.2014.08.004 (2015).

III. Supplementary Table

Supplementary Table S1. Comparative metabolomic results of urine specimens from HTK and non-HTK groups.

**Criteria**	**Peak pair number**
100 samples total	14642
Detected in more than 50% individual samples of each group	7136
P value < 0.01	4816
Significant metabolite peak pairs between two groups	1567
Fold change more than 5 (log_2 ratio beyond ± 2.32)	1280 [#]
All or none [##]	287

For pathway search	
MycompoundID hits in 1567 metabolites (MS search) [###]	562
After removal of metabolites without amine/phenol group	223
Pathway hits after MetaboAnalyst search (P< 0.05)	9

[#] This includes 1005 peak-pair metabolites that increased > 5 fold among HTK group and 275 peak-pair metabolites that decreased < 5 fold among HTK group.

[##] Metabolite peak-pair detected in only one clinical group.

[###] Compound identification using molecular mass in the form of neutral mass (Da), m/z values of positive and negative ions, including protonated molecules and common adduct ions.

Supplementary Table S2. Altered metabolic pathways and the number of metabolite hits after HTK treatment

Pathway name	Total	Hit	*p*-value
Pyrimidine metabolism	60	17	<0.001
Purine metabolism	92	19	<0.001
Histidine metabolism	44	9	0.002
Nitrogen metabolism	39	8	0.003
Alanine, Aspartate and Glutamate metabolism	24	6	0.003
Vitamin B_6 metabolism	32	6	0.014
Tyrosine metabolism	76	10	0.021
Arginine and Proline metabolism	77	10	0.024
Glutamine and Glutamate metabolism	11	3	0.029

IV. Supplementary Figure

Supplementary Fig. S1 The inverse relationship between the percentage of common peak-pairs detected and total peak-pair numbers in 100 urine samples. As the number of samples increased, the number of commonly detected peak-pairs decreased and the total number of detected peak-pairs increased slightly. A total of 2114 peak-pairs were detected in more than 80% of samples. A total of 11,728 peak pairs were detected in at least 10 samples, indicating the diversity of urinary metabolites among cardiac surgery patients.

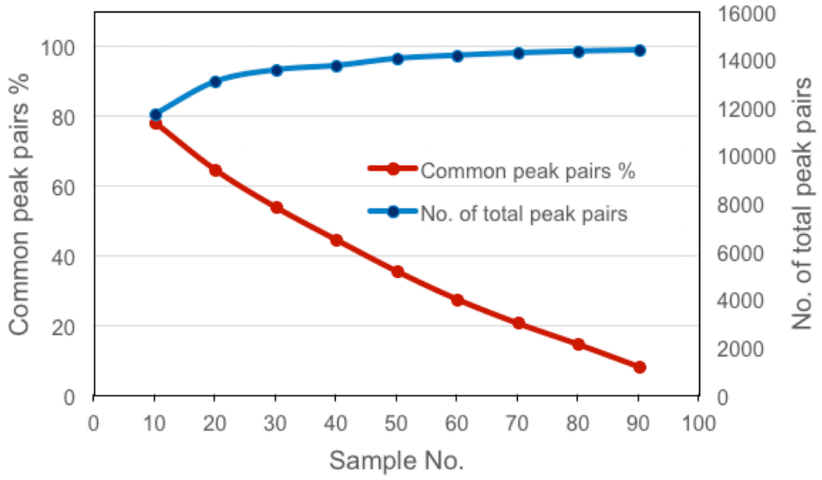

Common peak pairs			Total peak pairs	
Sample No.	%	Numbers	Sample No.	Numbers
>90	8	1194	10	11728
>80	15	2114	20	13069
>70	21	2988	30	13556
>60	28	3974	40	13738
>50	36	5135	50	14031
>40	45	6434	60	14162
>30	54	7780	70	14264
>20	65	9322	80	14337
>10	78	11283	90	14385
>0	100	14416	100	14416

Supplementary Fig. S2 Scatter plots of the relative concentration ratios of selected metabolites in the non-HTK group (blue, n = 51) versus the HTK group (red, n = 49). Log_2 fold changes are denoted as arrows and values (HTK/Non-HTK). (a) 1-methyladenosine; (b) 3-methylhistidine.

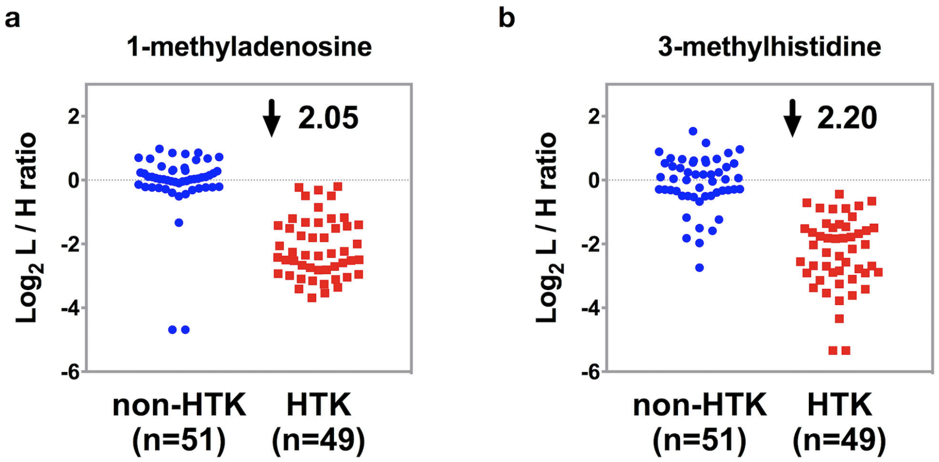

Supplementary Fig. S3 Heatmap of hierarchical clustering of 1567 differential expressed metabolite peak pairs (HTK vs. non-HTK group). Each column represents an individual, and each row represents a metabolite with its corresponding peak pair number. The color scale is $\log_2$ transformed value and indicates relative high (red) and low (green) metabolite level. For easy visualization of detailed information about metabolite peak pair number in the left side, we then split Supplementary Fig.3 into multiple part and label as Supplementary Fig S3-1~S3-57 in the following pages.

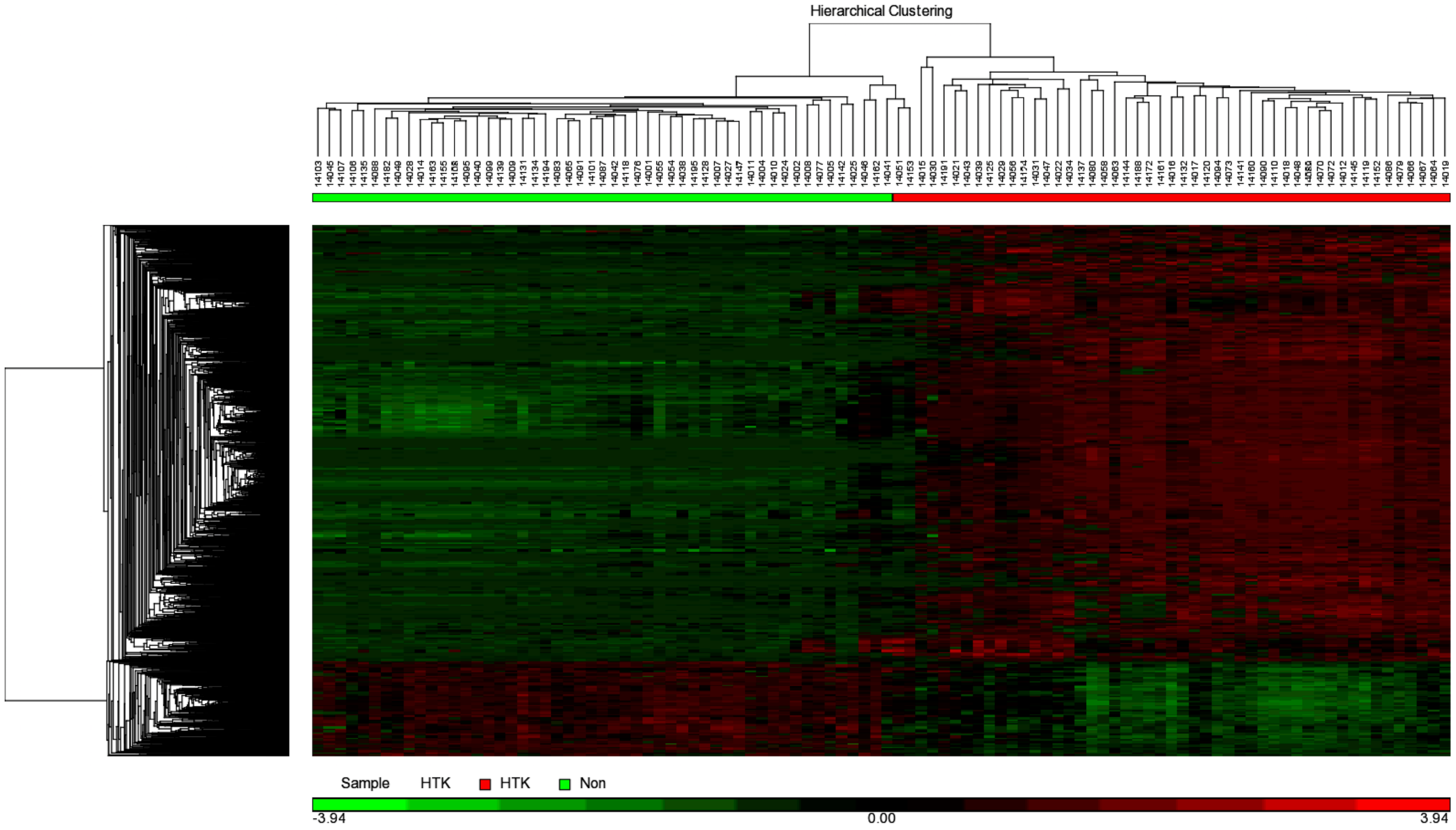

V. Supplementary Figure S3-1 ~ S3-57

Supplementary Fig. S3-1

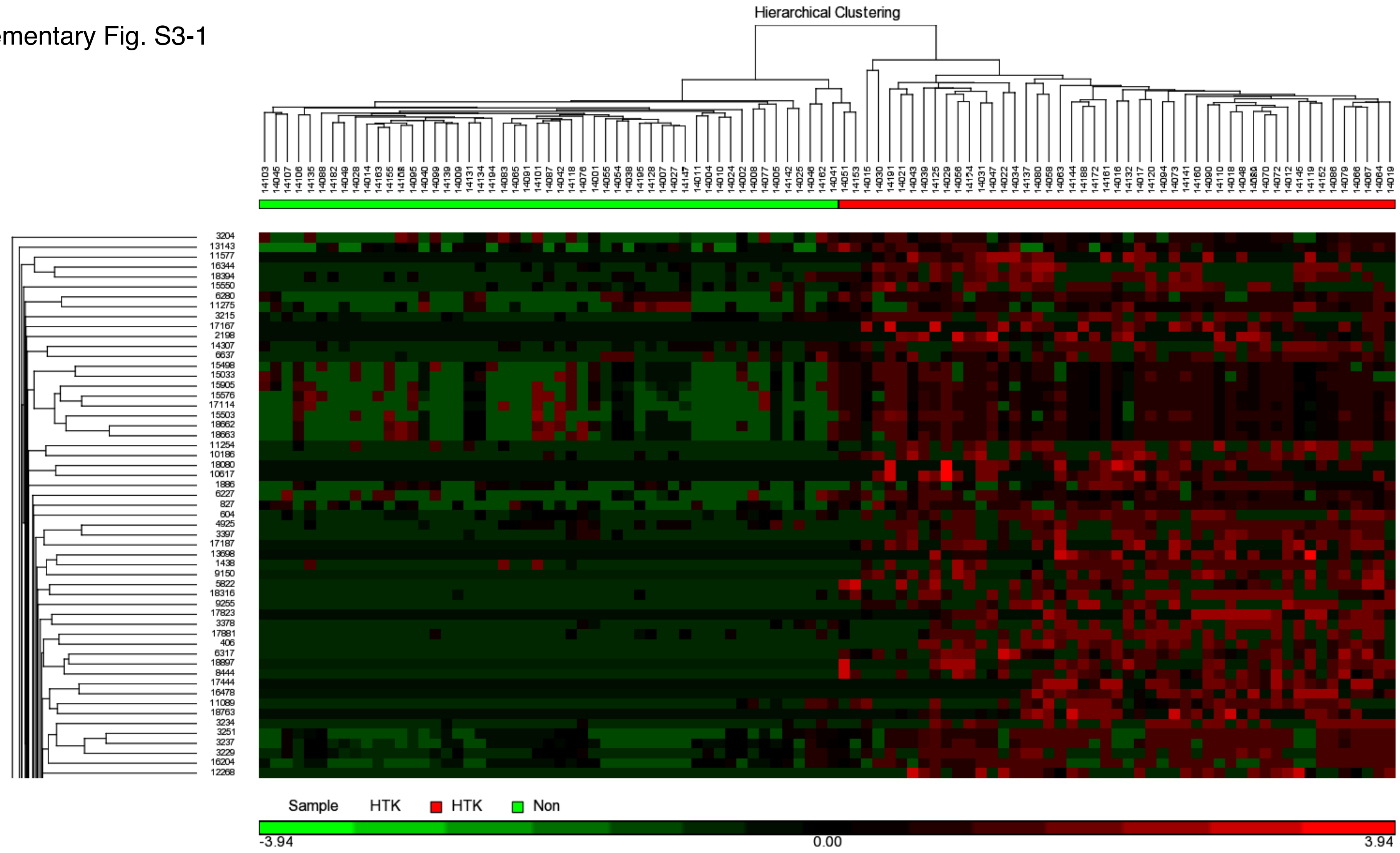

Supplementary Fig. S3-2

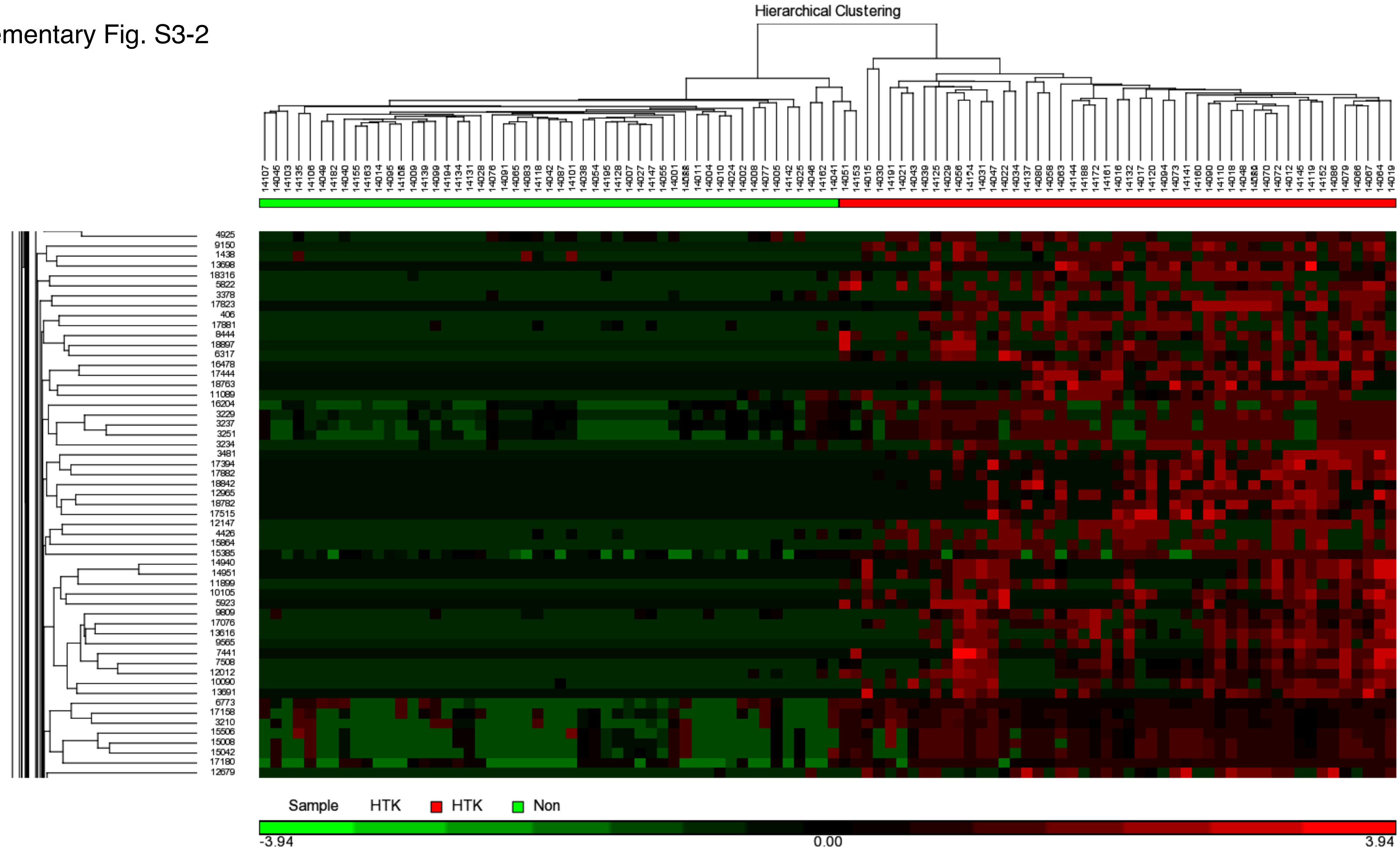

Supplementary Fig. S3-3

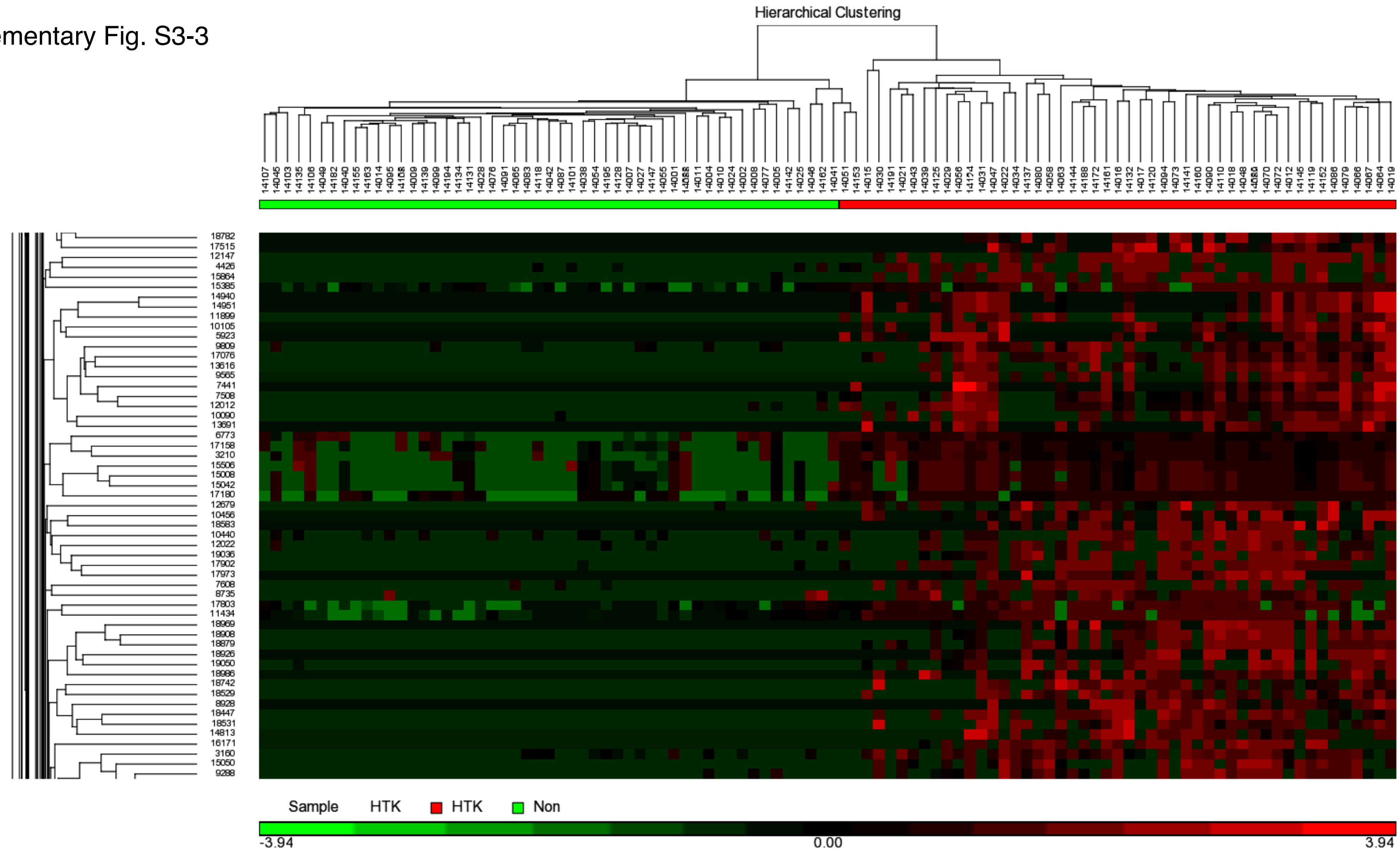

Supplementary Fig. S3-4

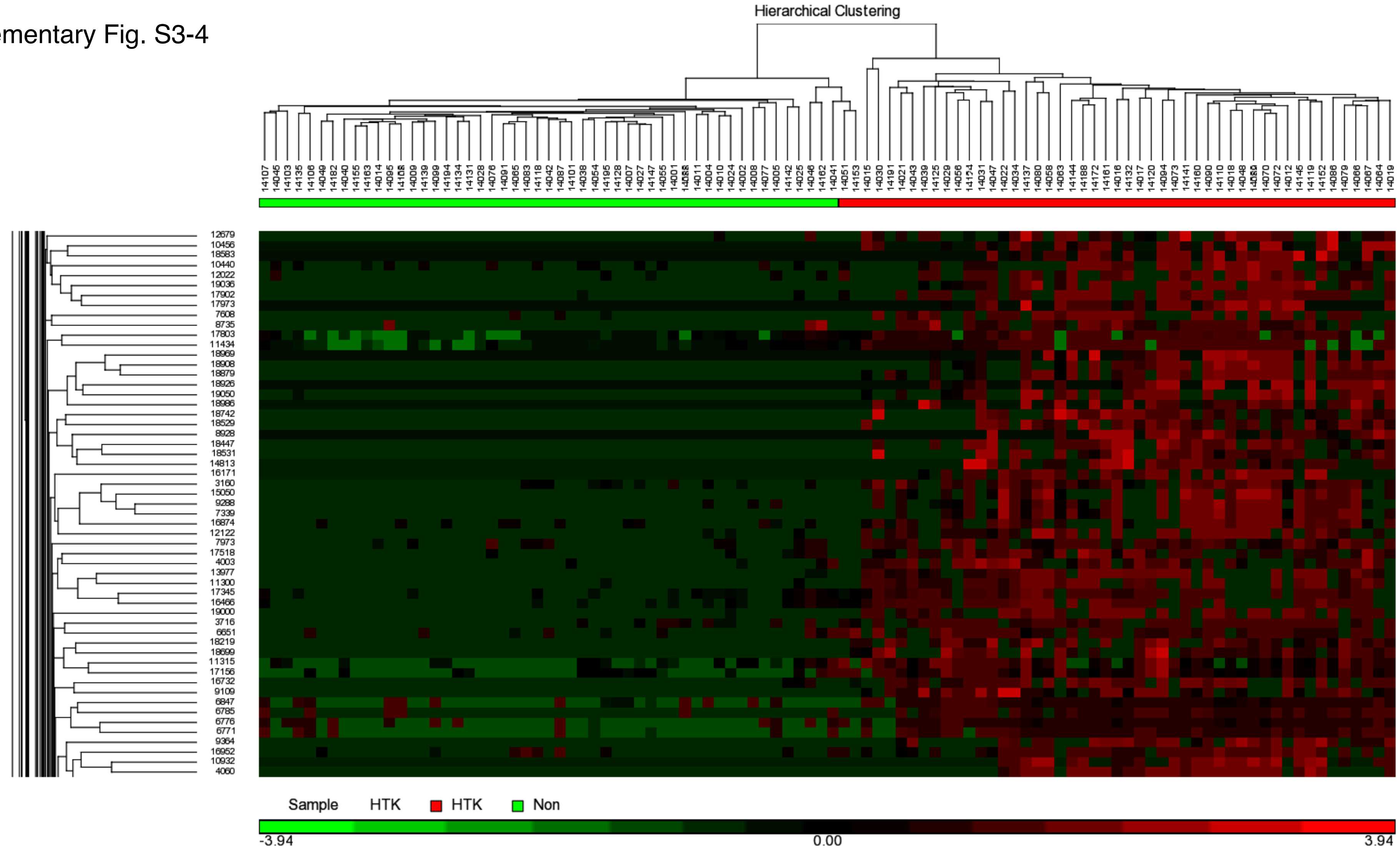

Supplementary Fig. S3-5

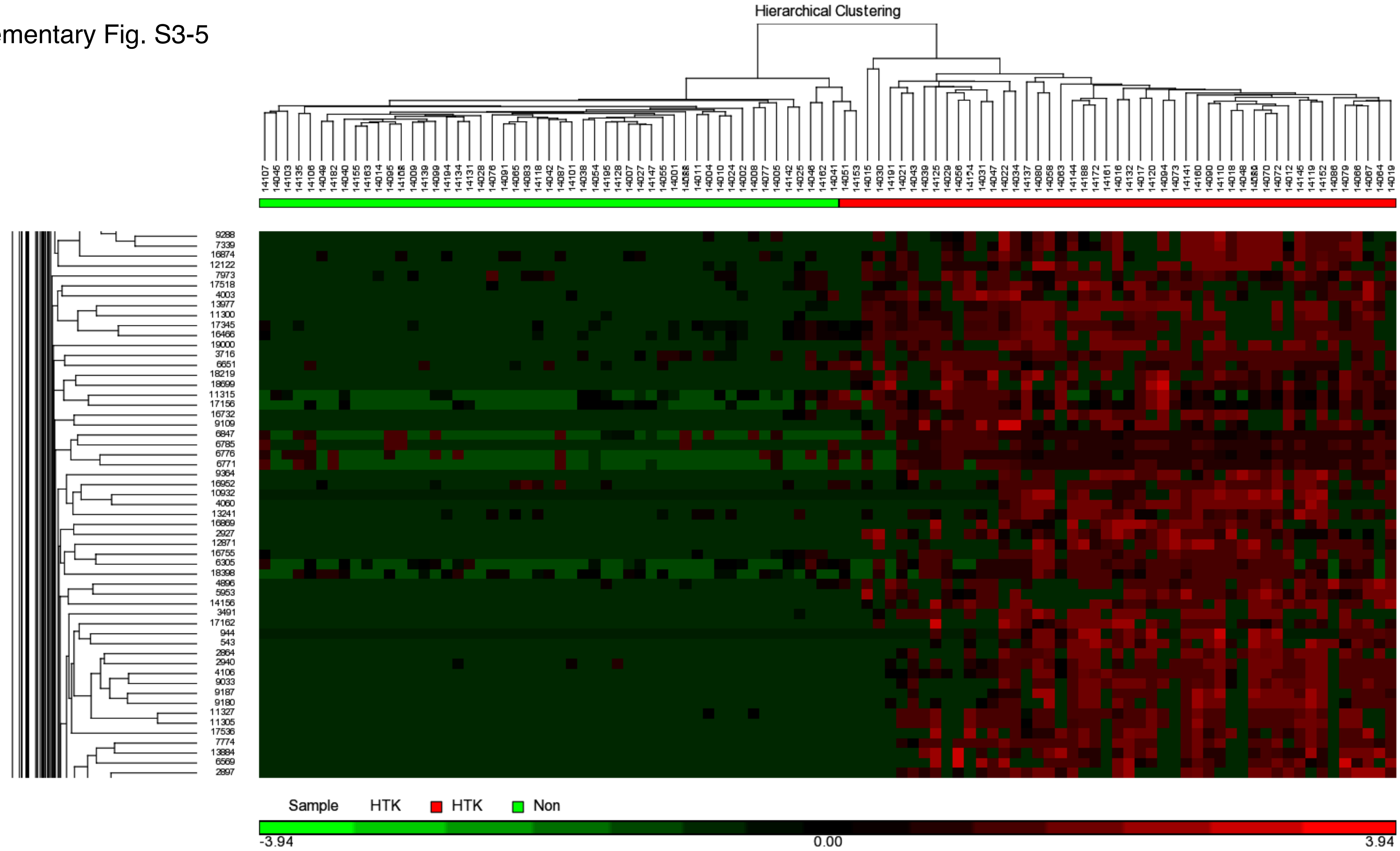

Supplementary Fig. S3-6

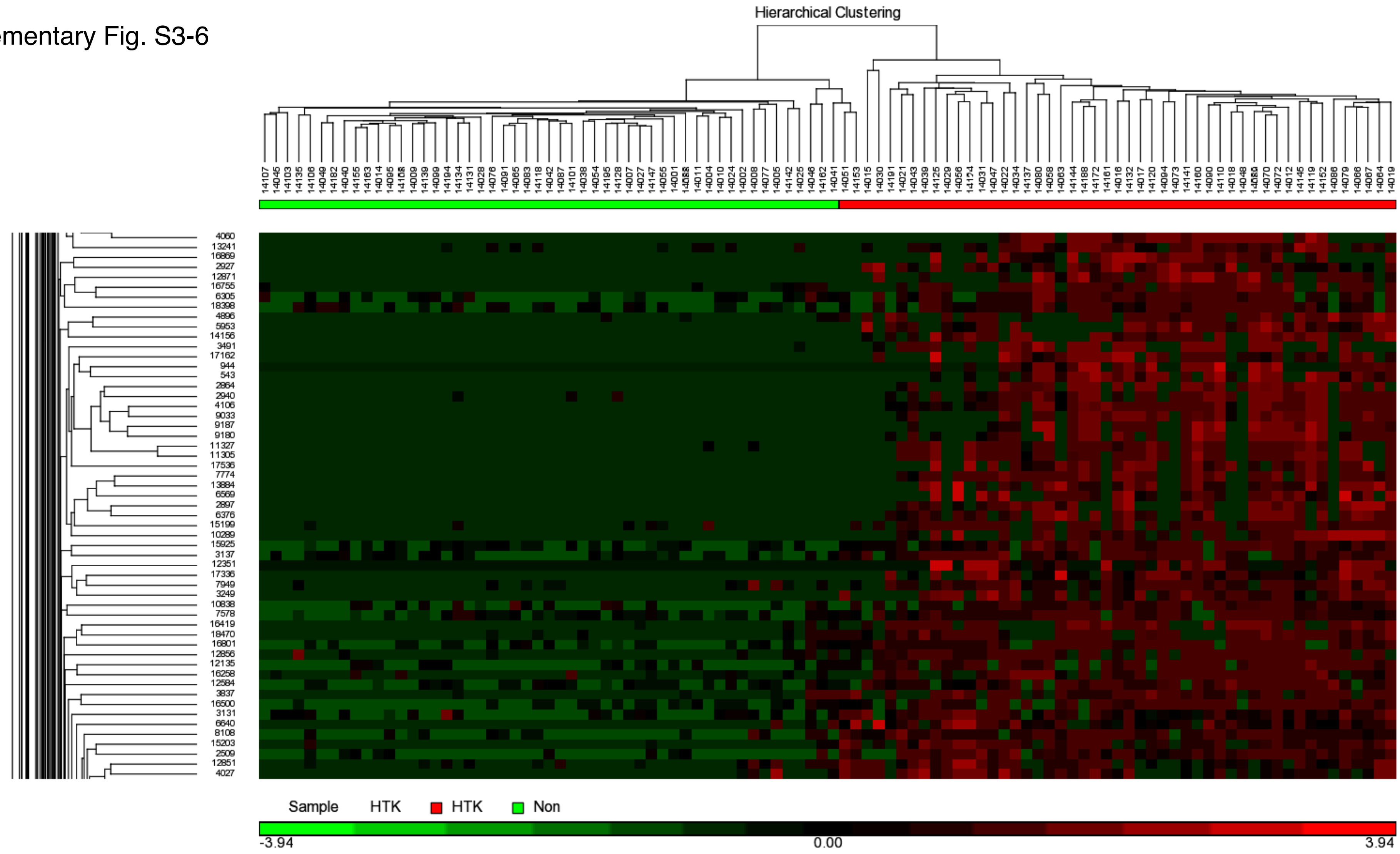

Supplementary Fig. S3-7

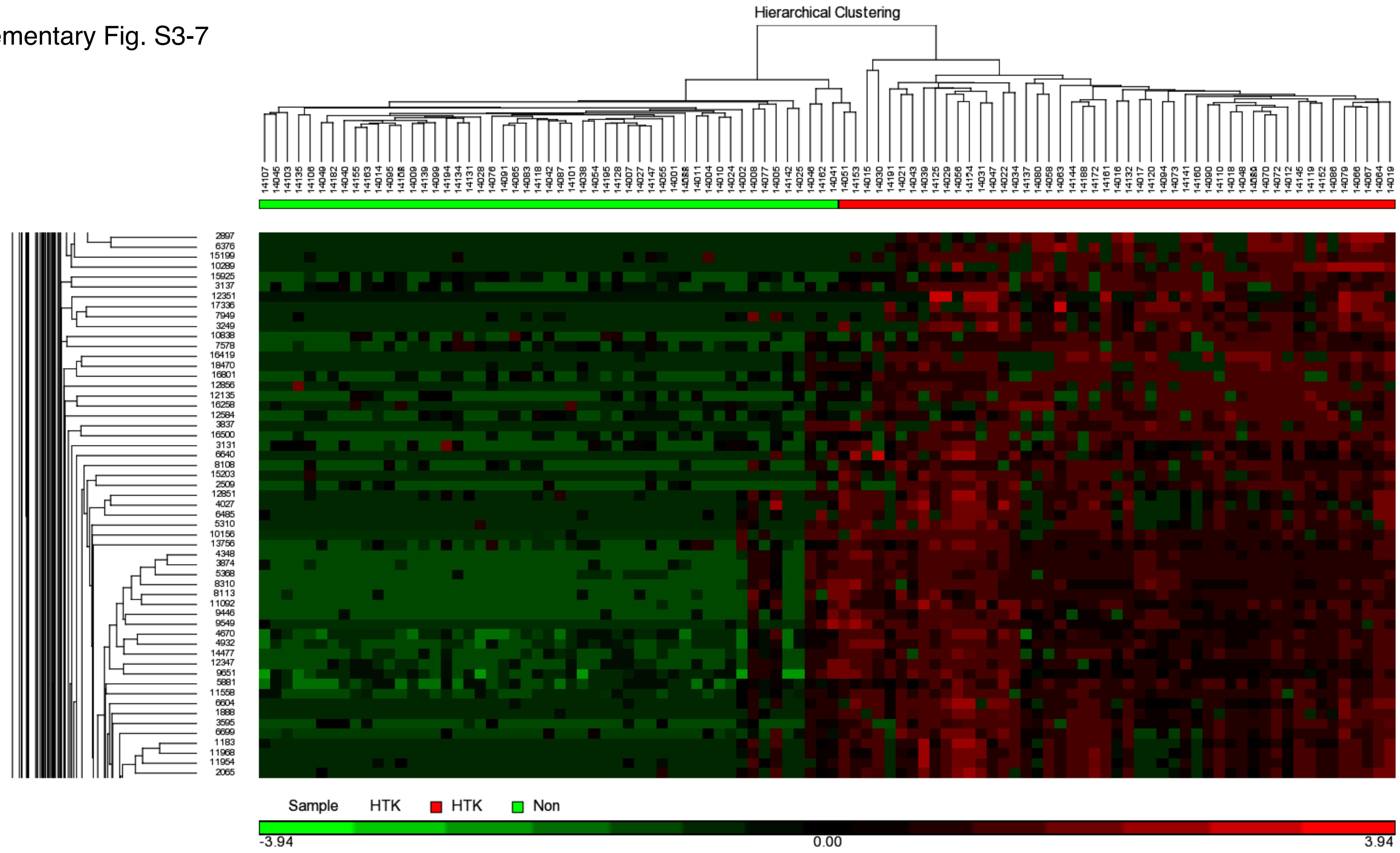

Supplementary Fig. S3-8

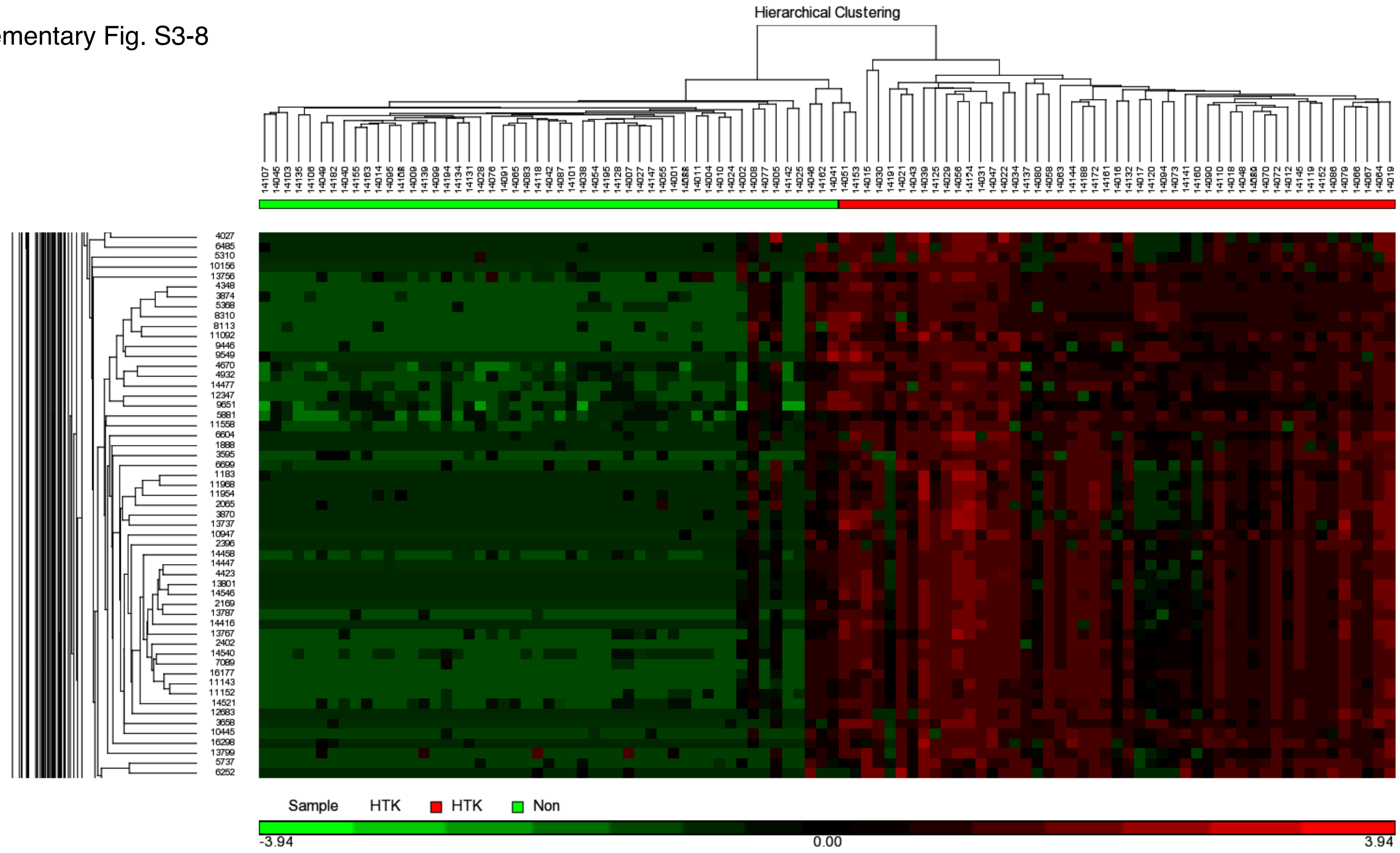

Supplementary Fig. S3-9

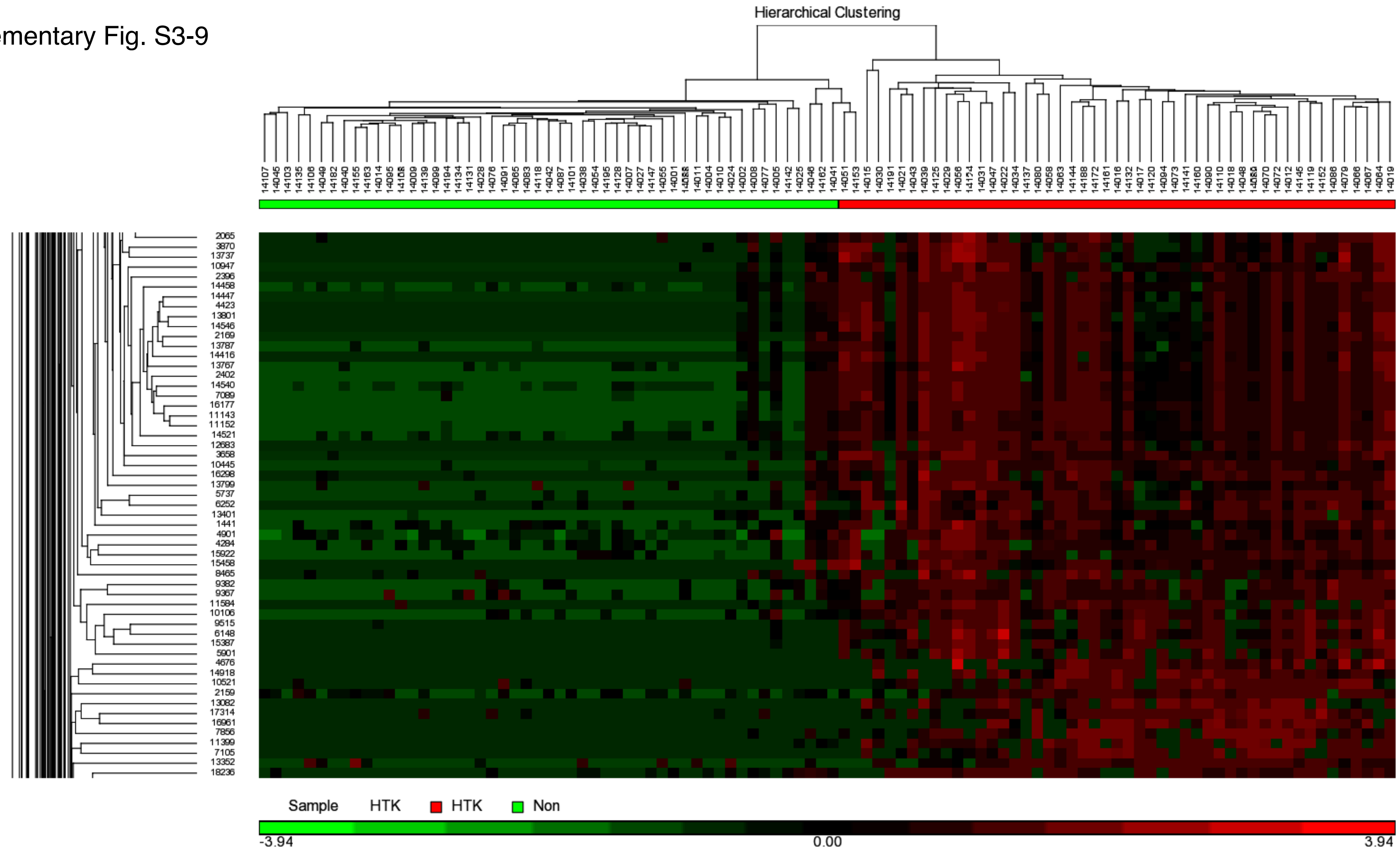

Supplementary Fig. S3-10

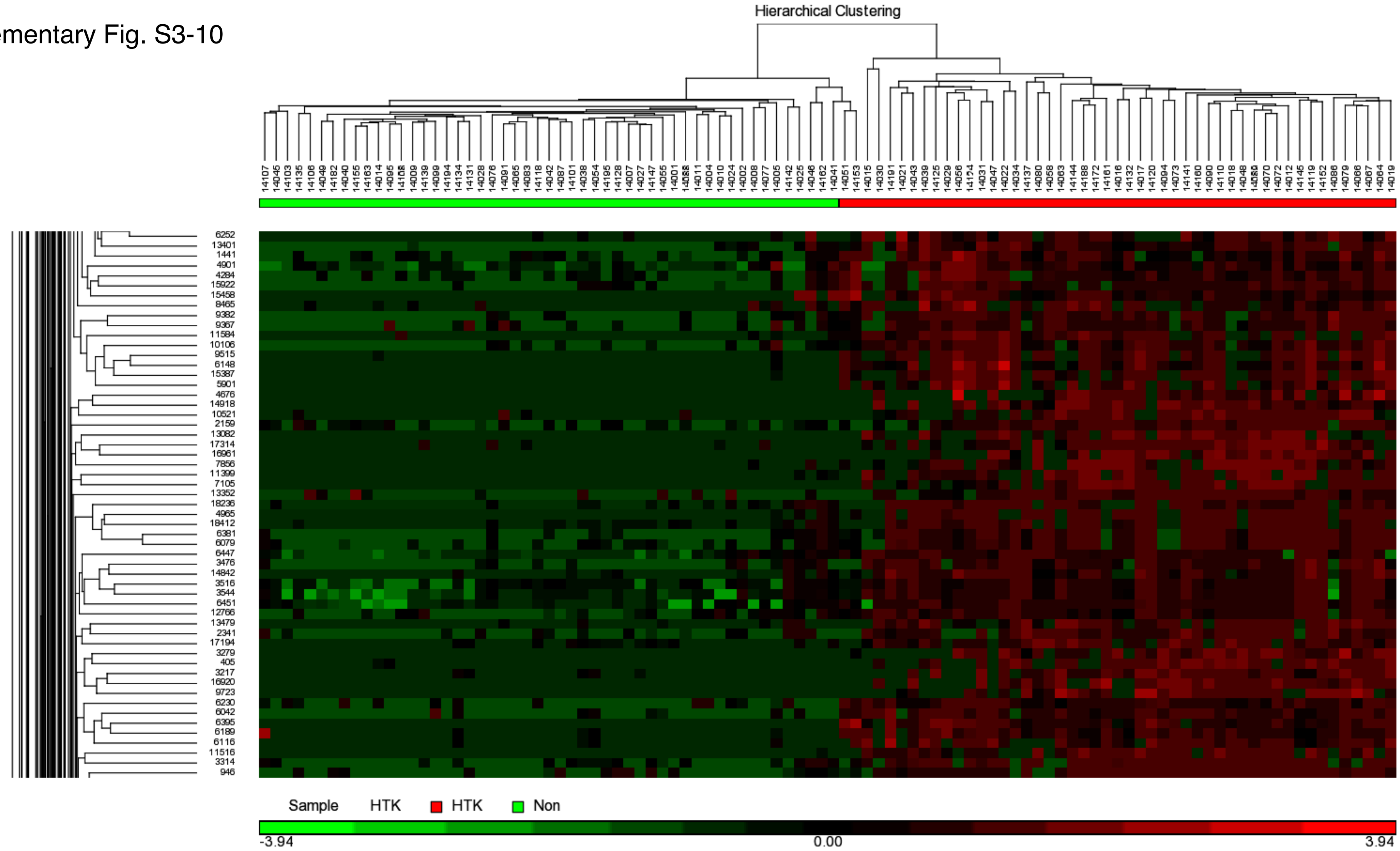

Supplementary Fig. S3-11

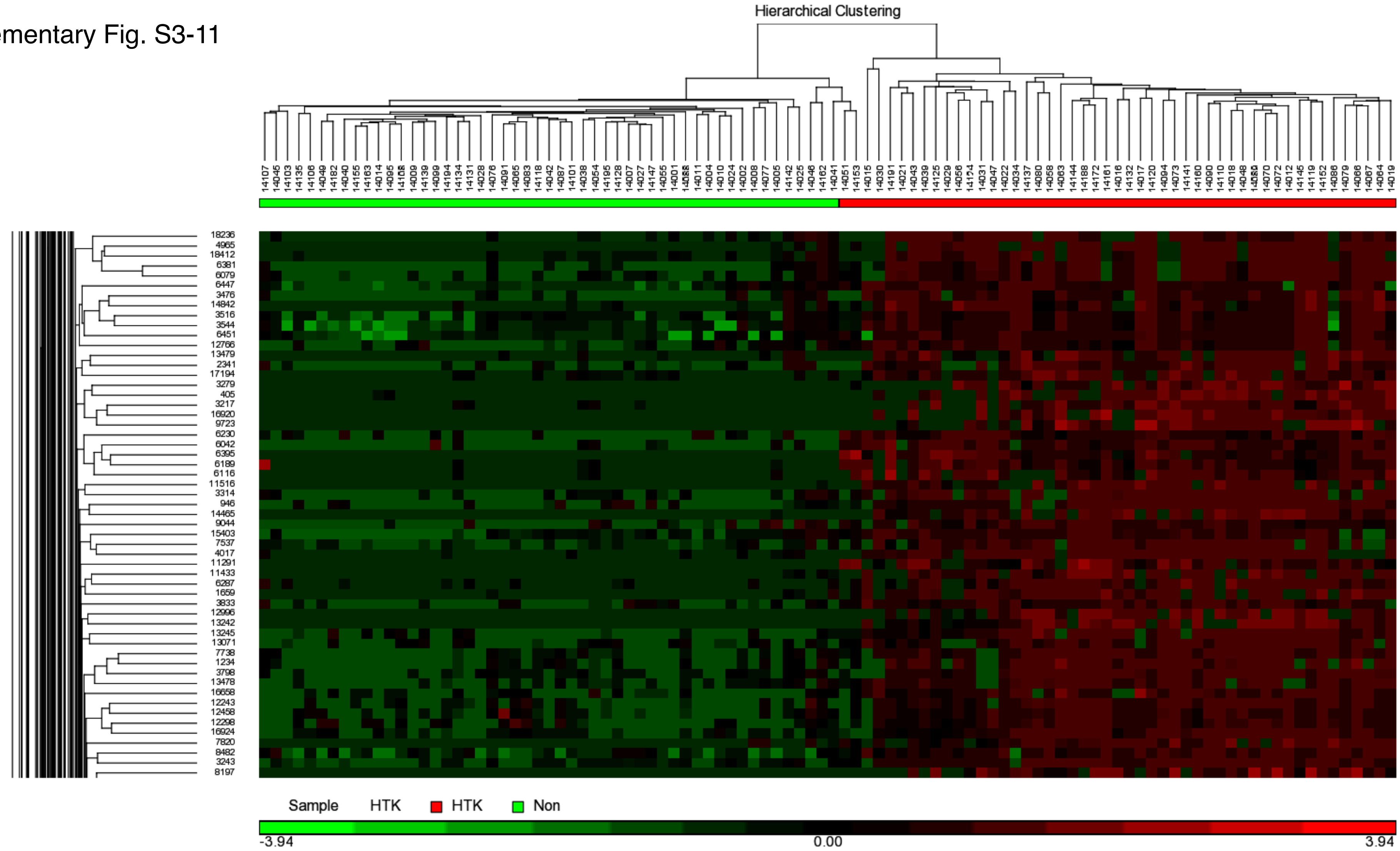

Supplementary Fig. S3-12

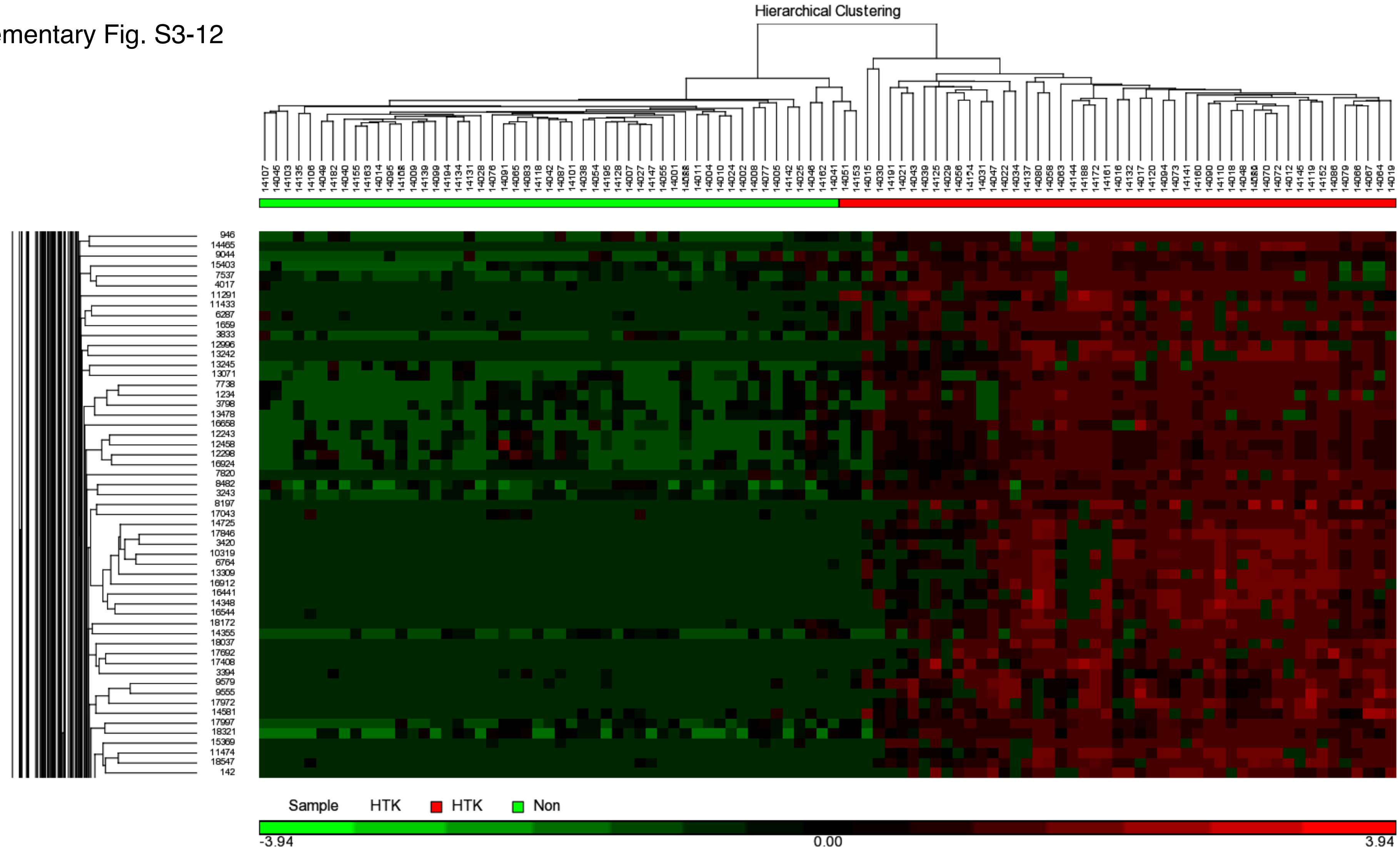

Supplementary Fig. S3-13

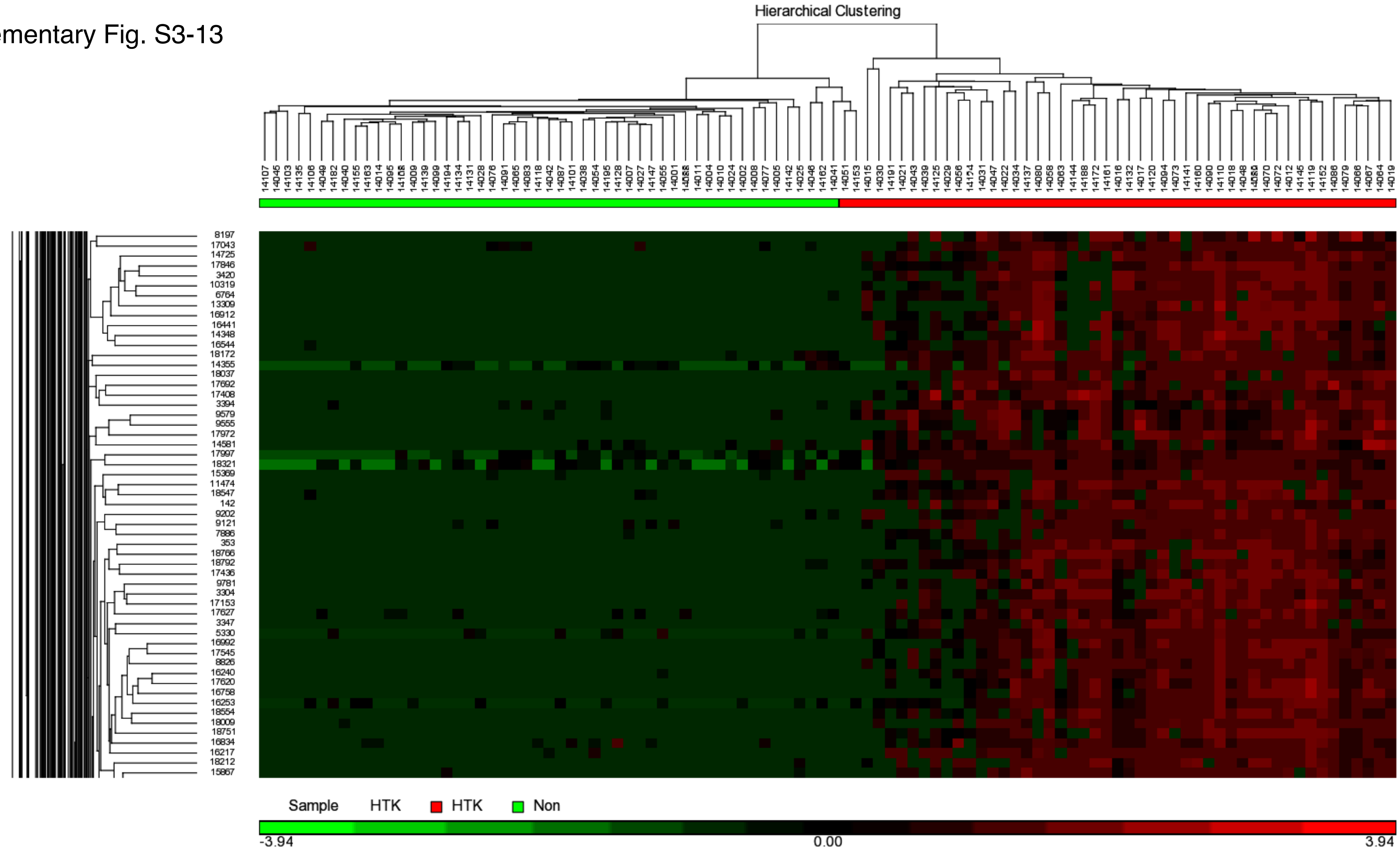

Supplementary Fig. S3-14

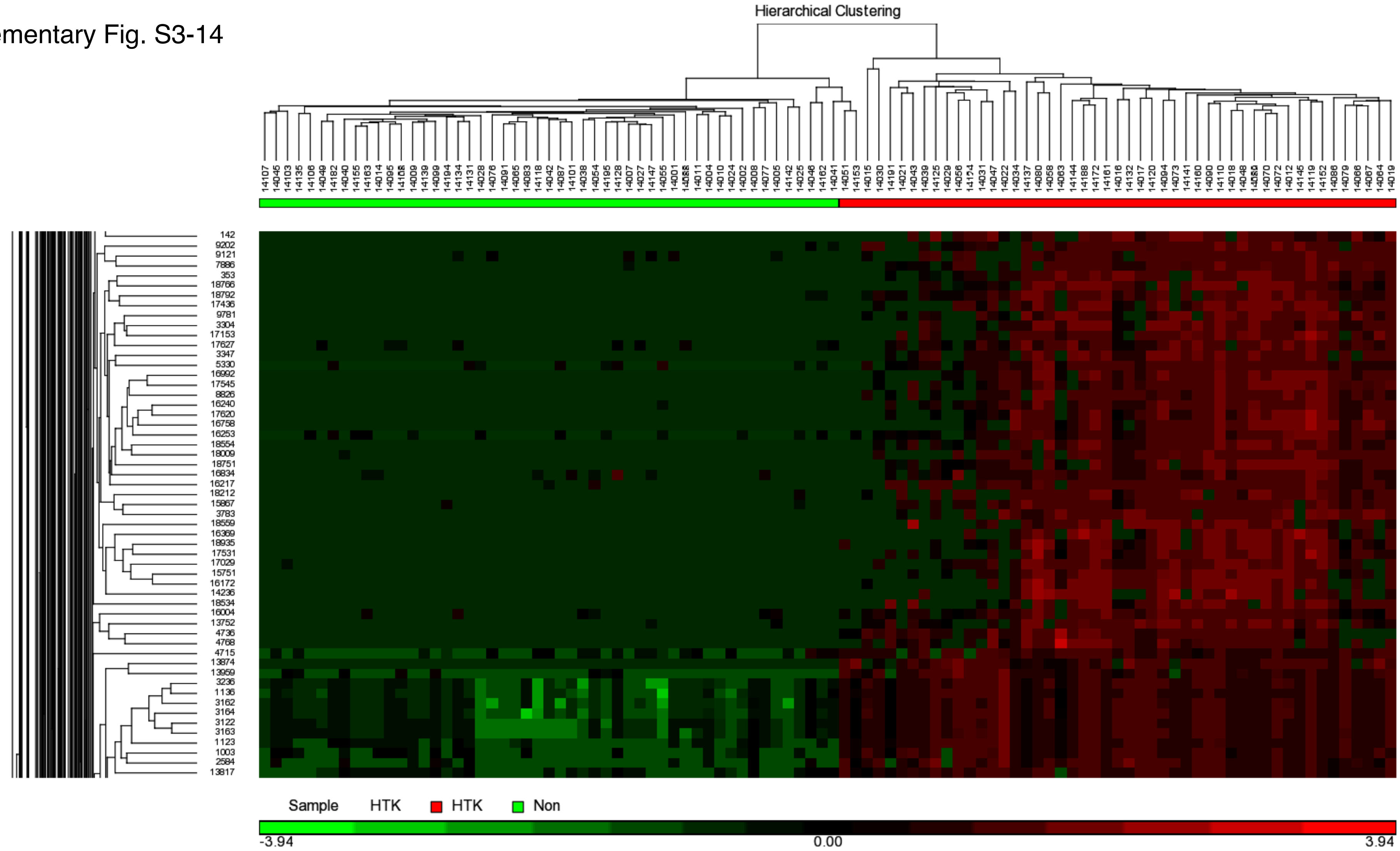

Supplementary Fig. S3-15

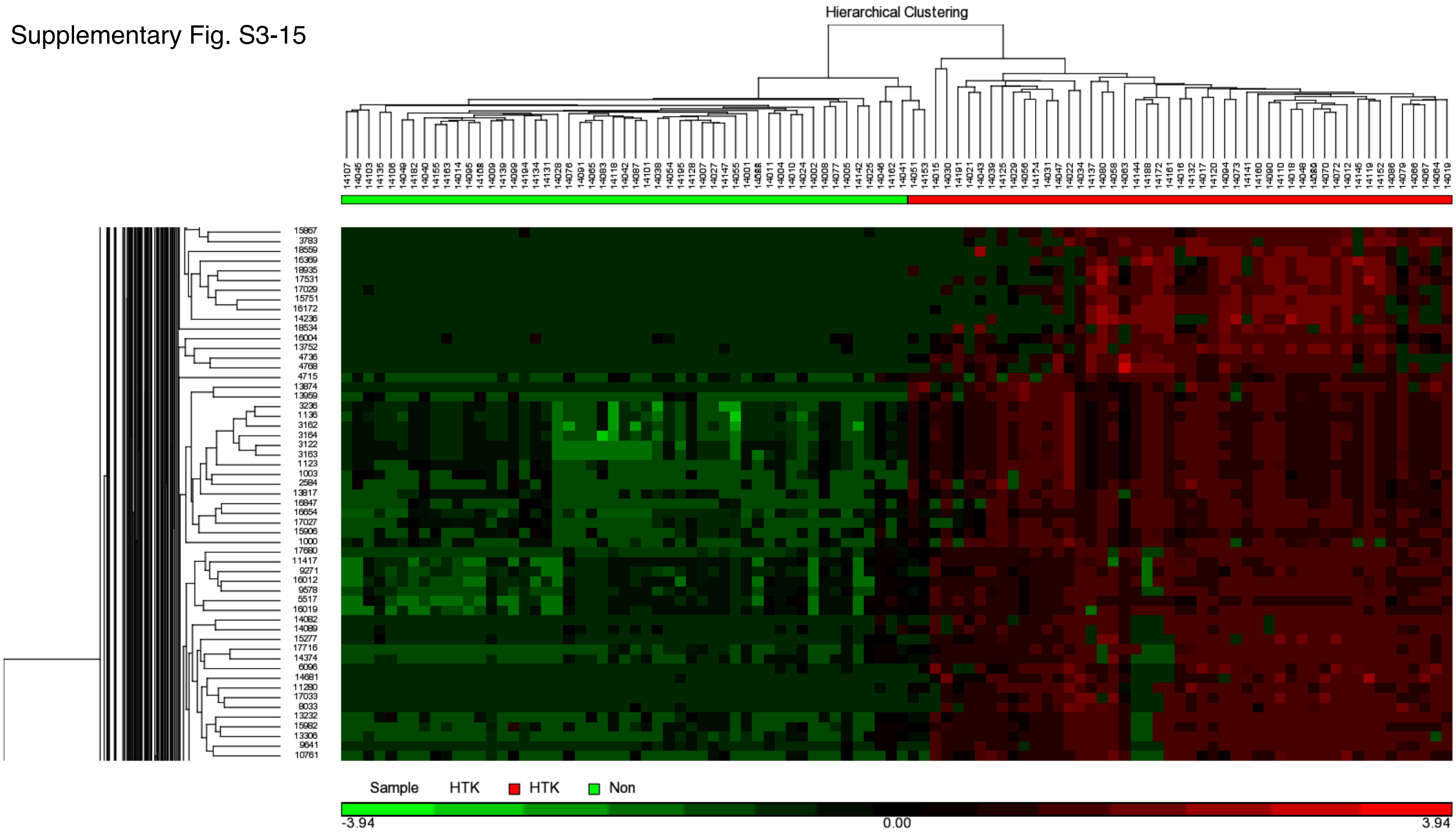

Supplementary Fig. S3-16

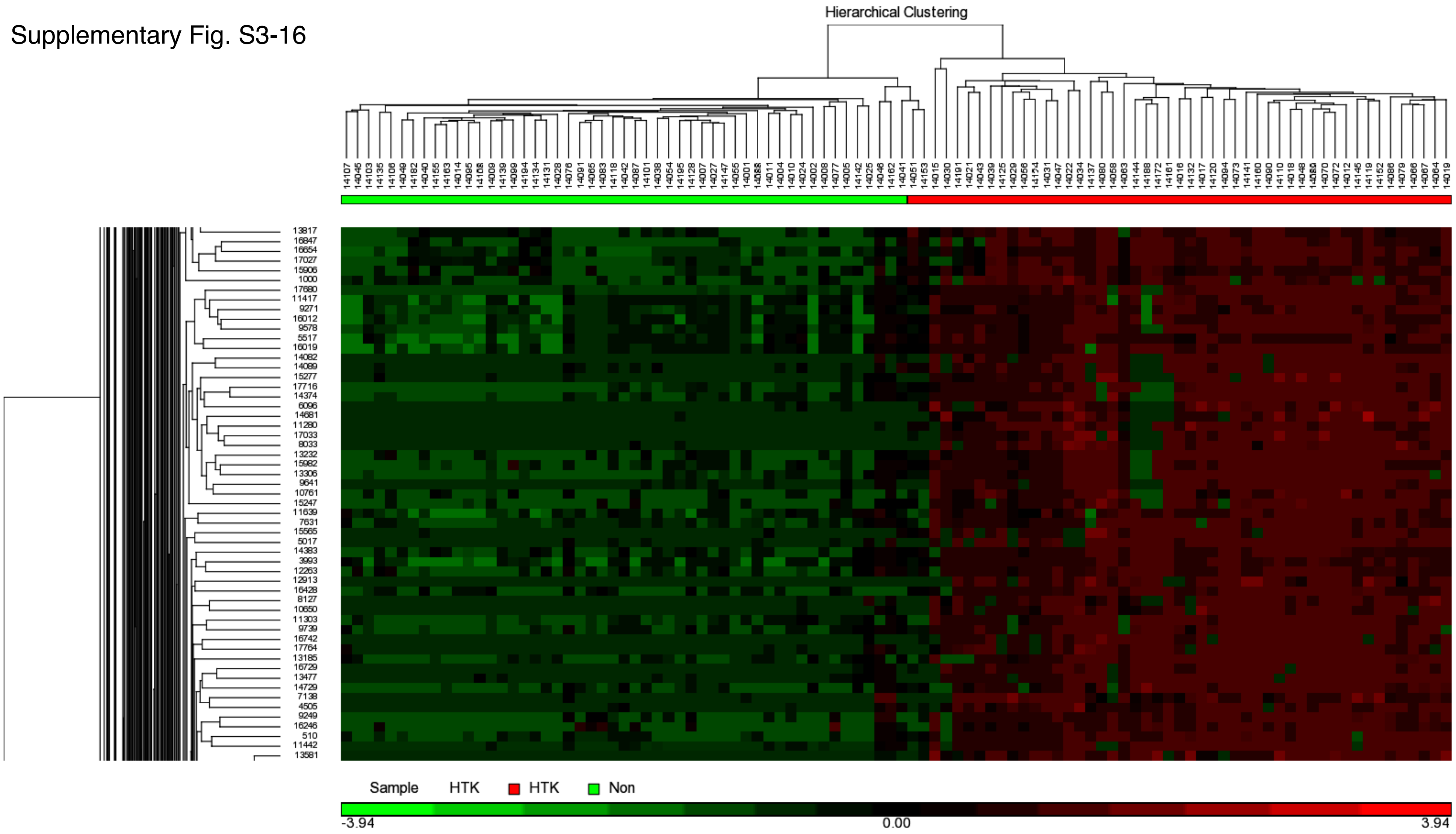

Supplementary Fig. S3-17

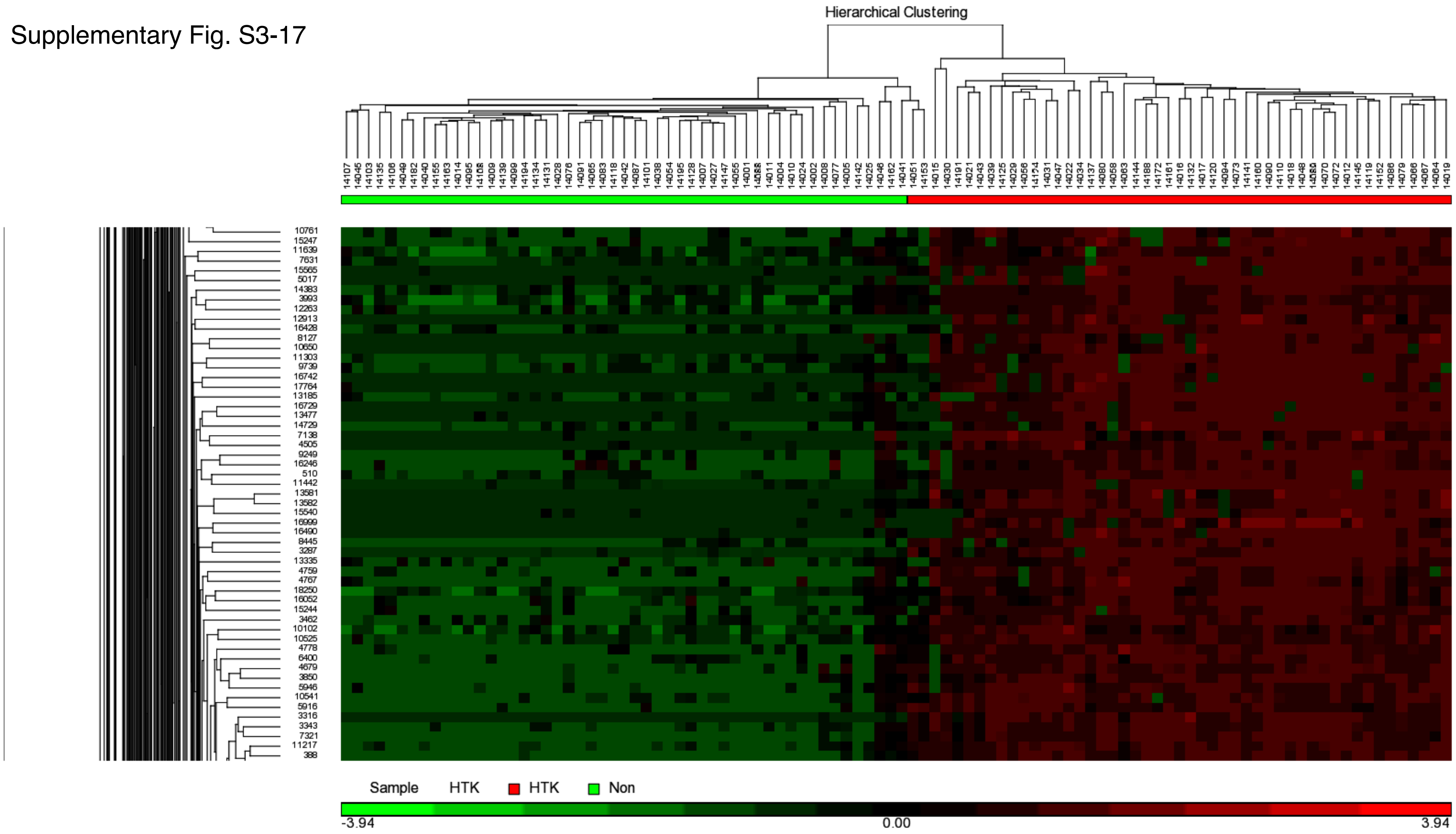

Supplementary Fig. S3-18

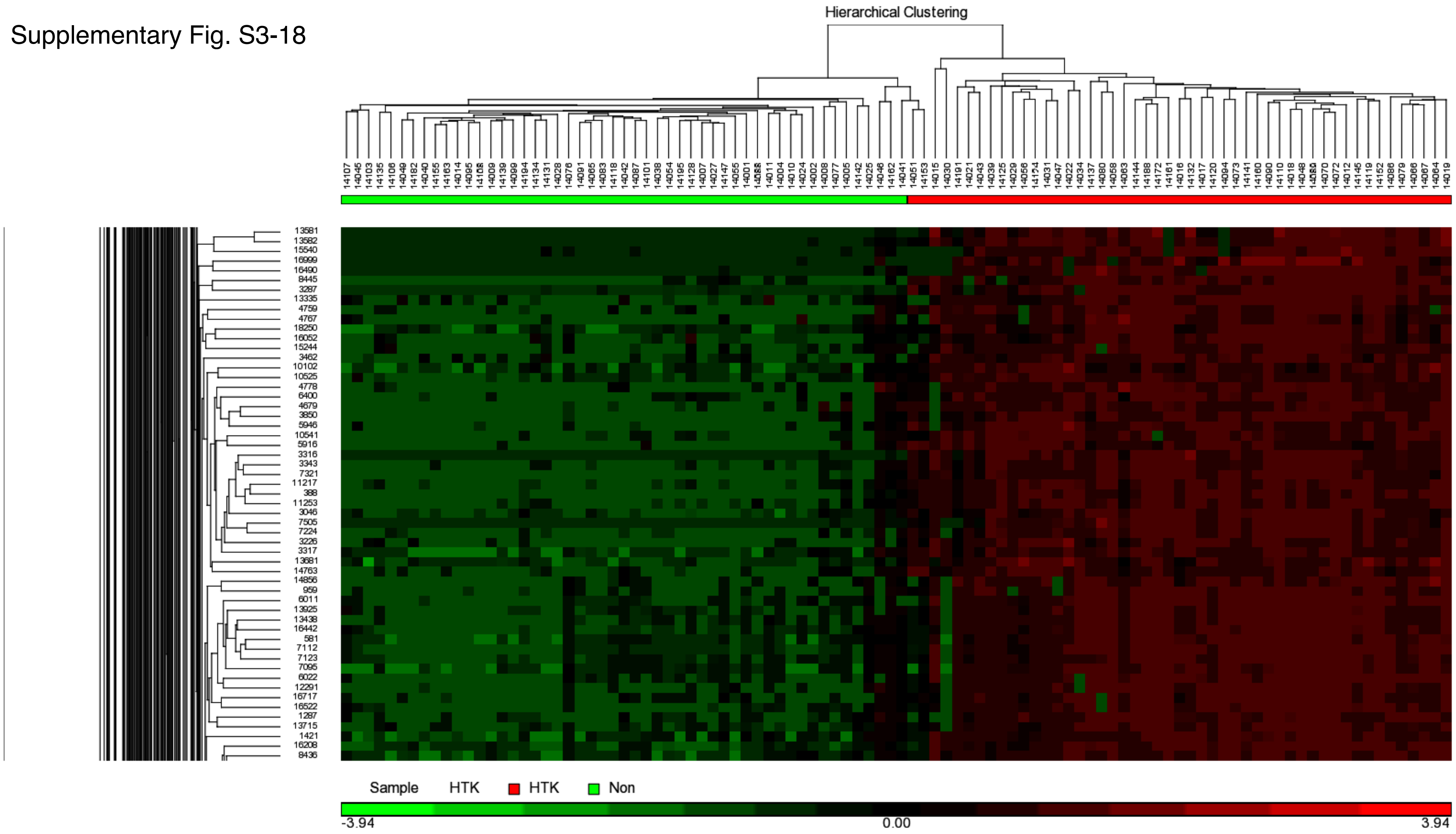

Supplementary Fig. S3-19

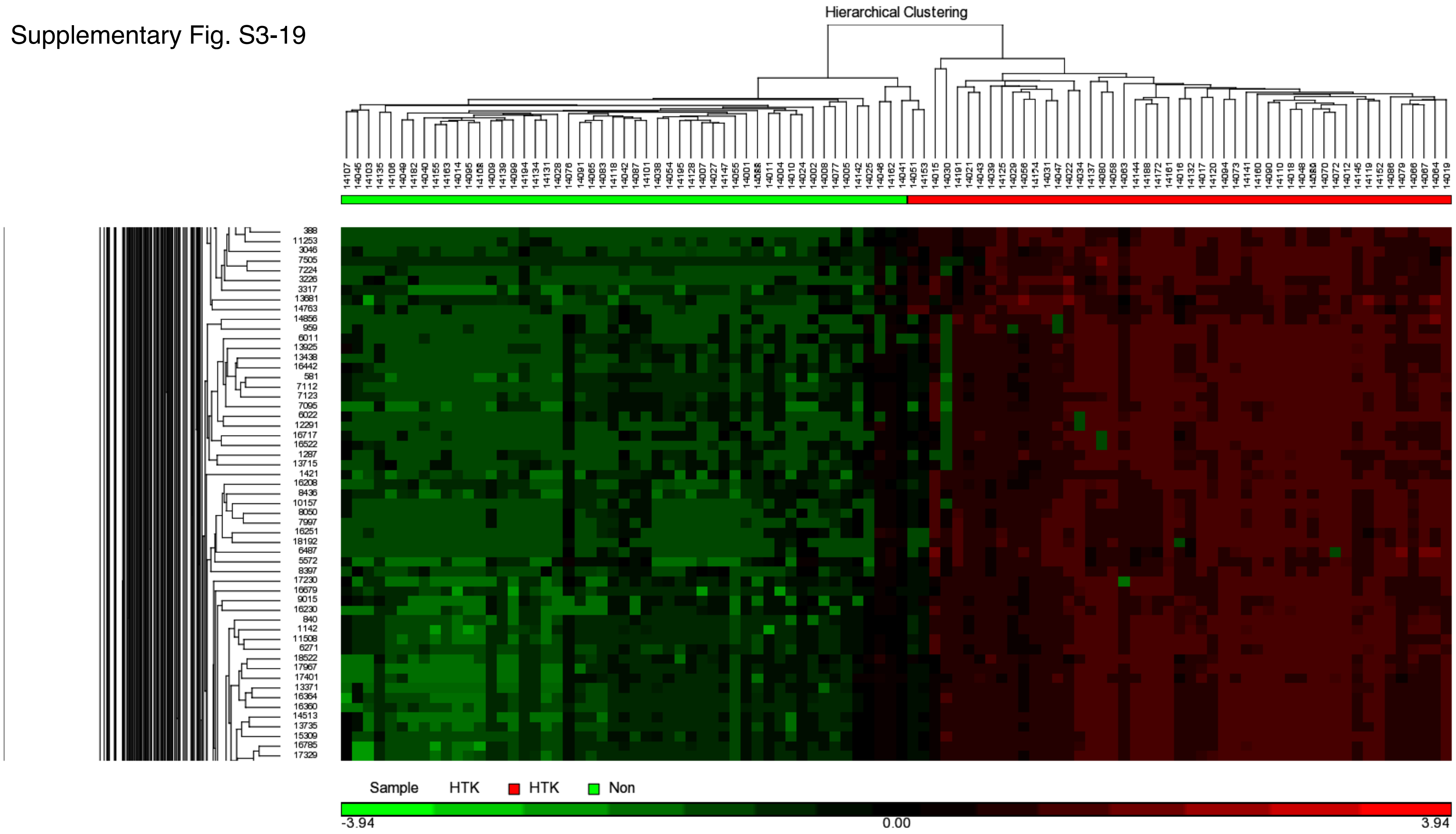

Supplementary Fig. S3-20

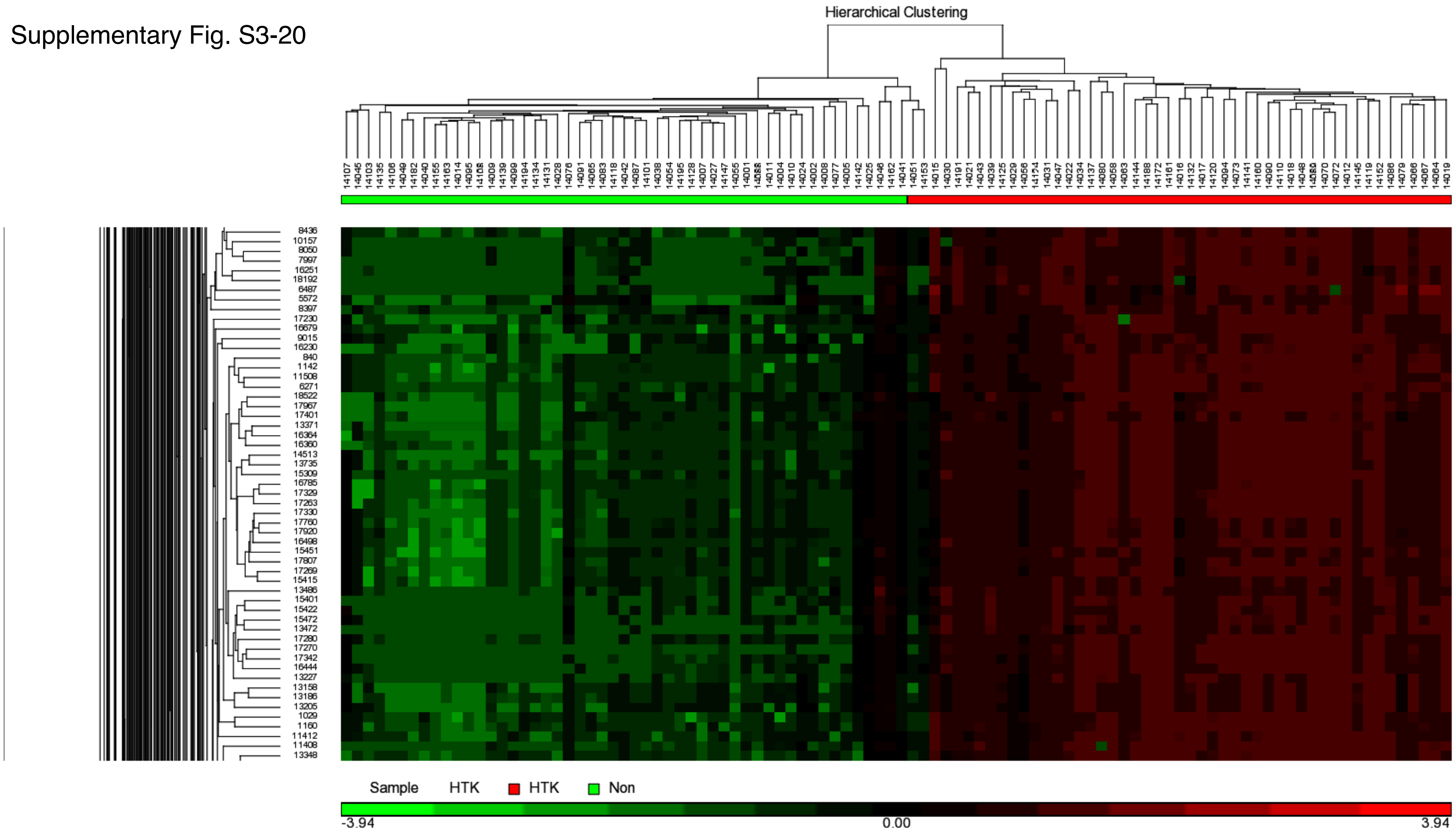

Supplementary Fig. S3-21

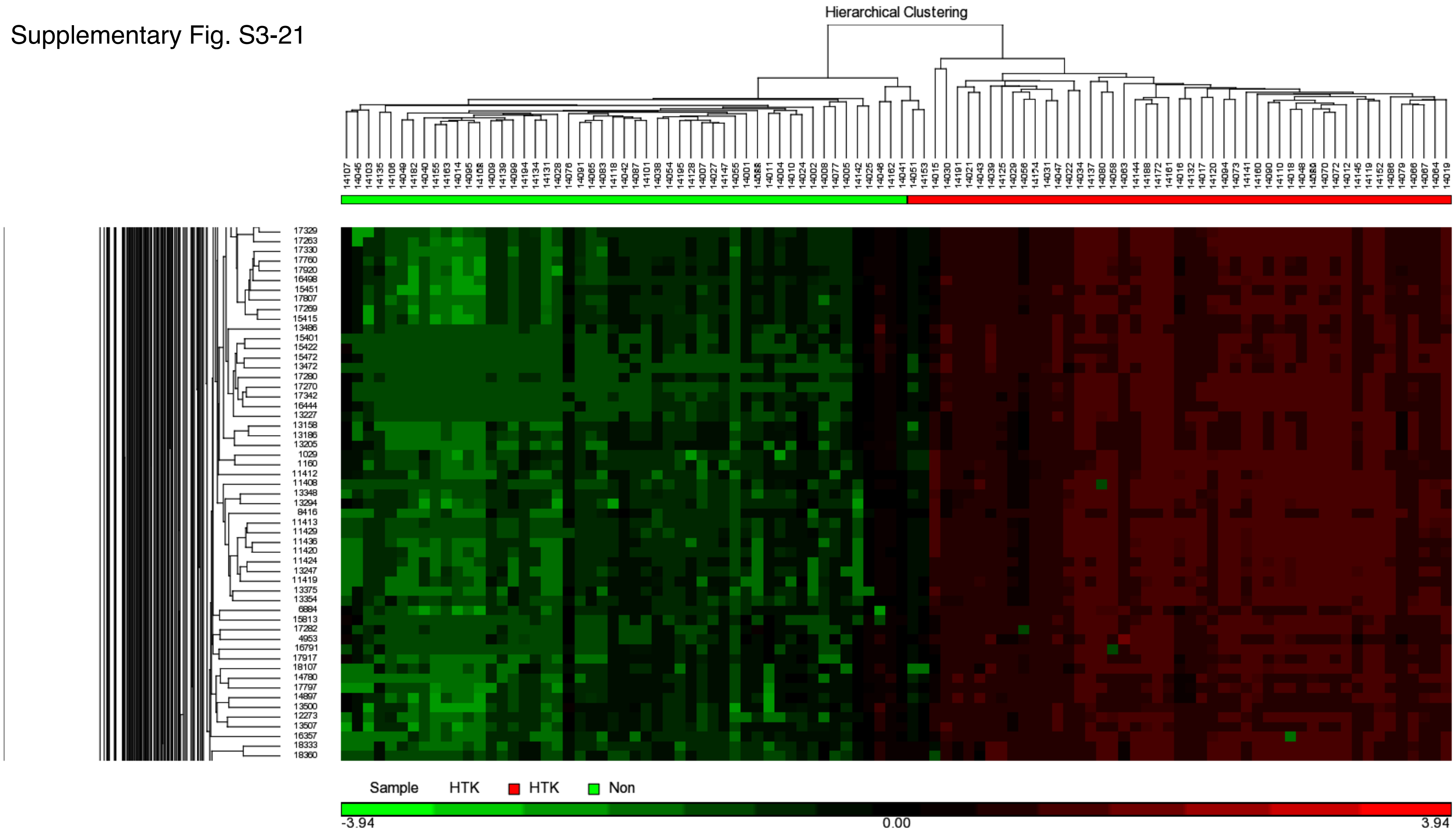

Supplementary Fig. S3-22

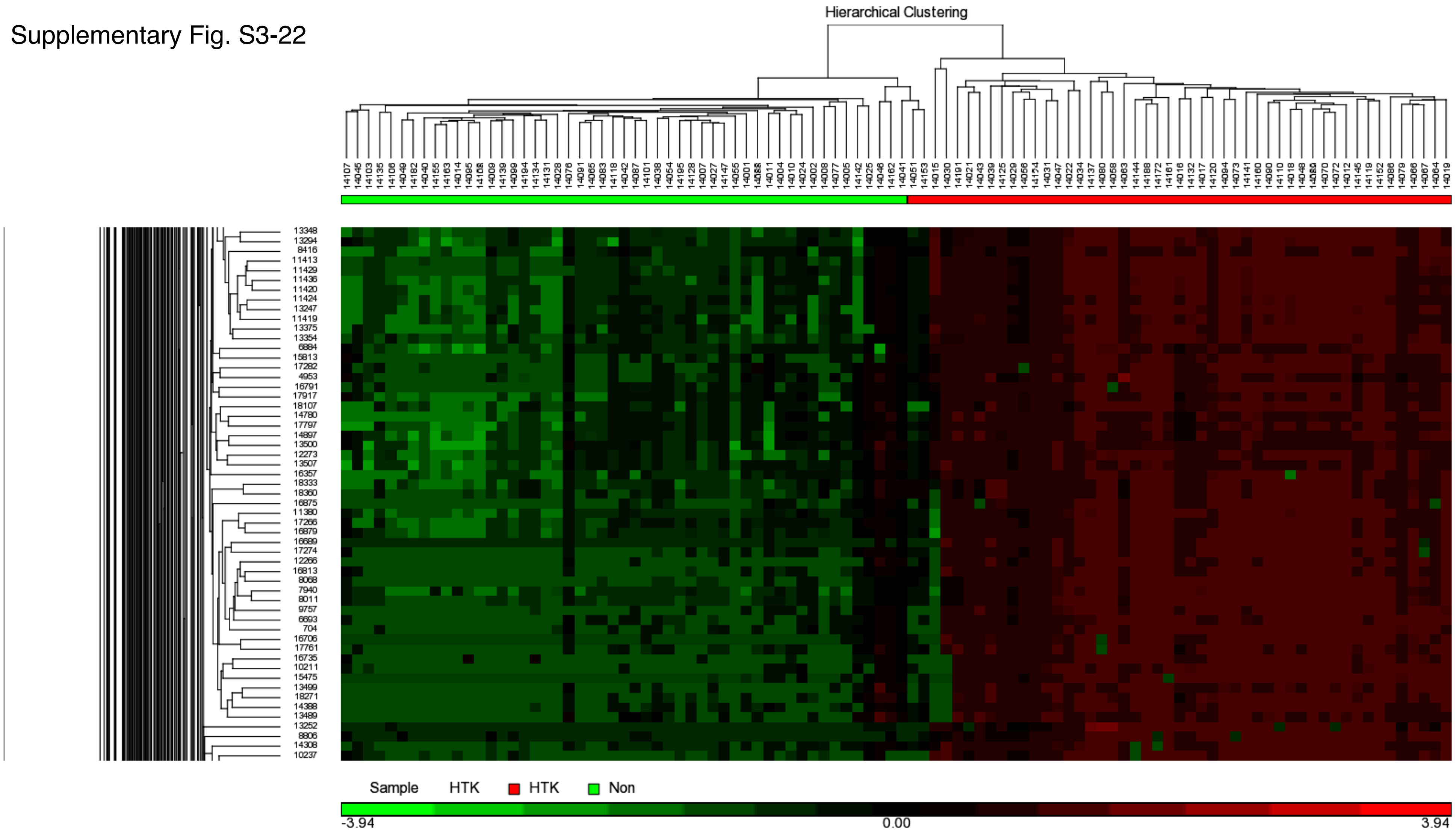

Supplementary Fig. S3-23

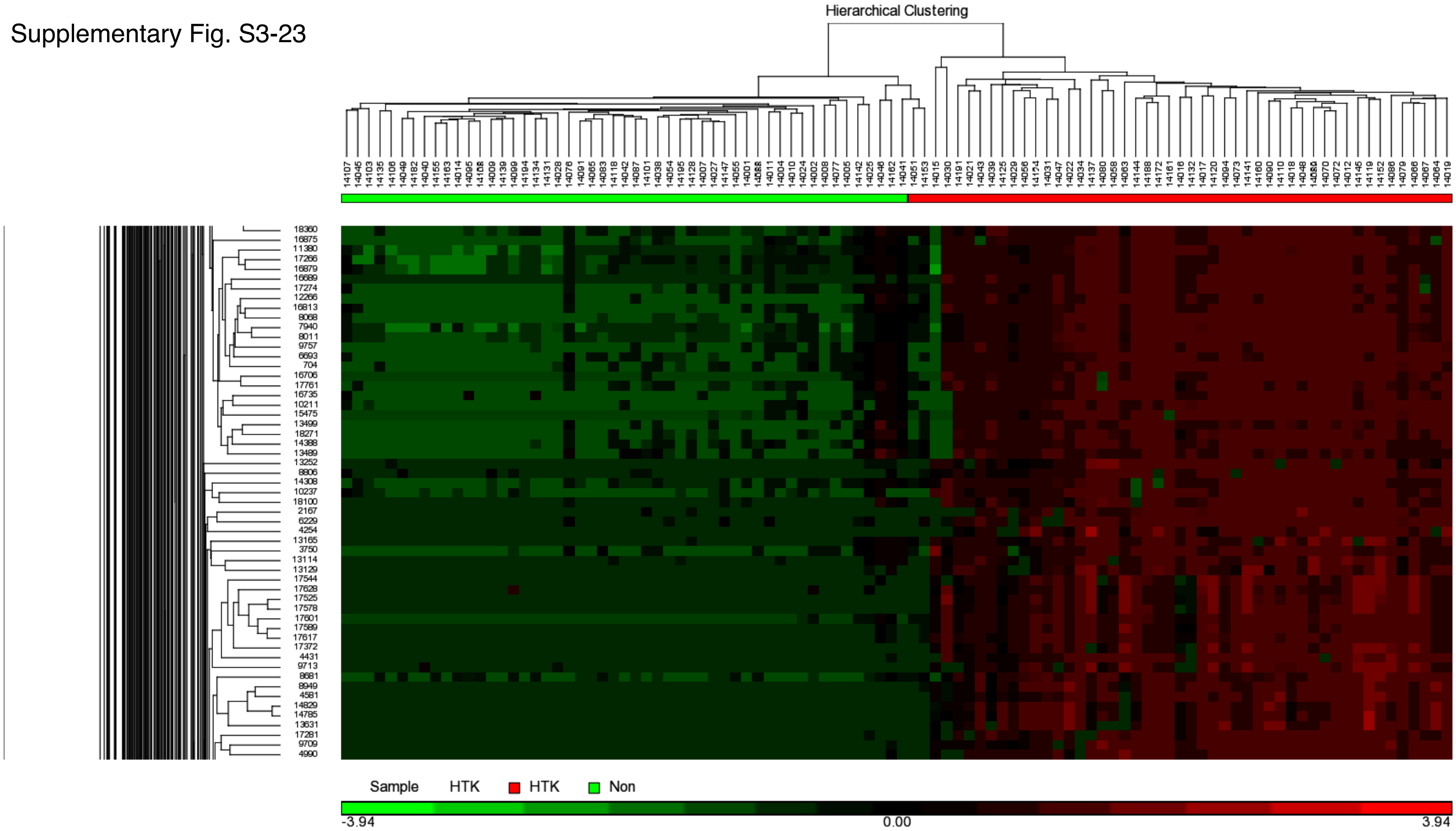

Supplementary Fig. S3-24

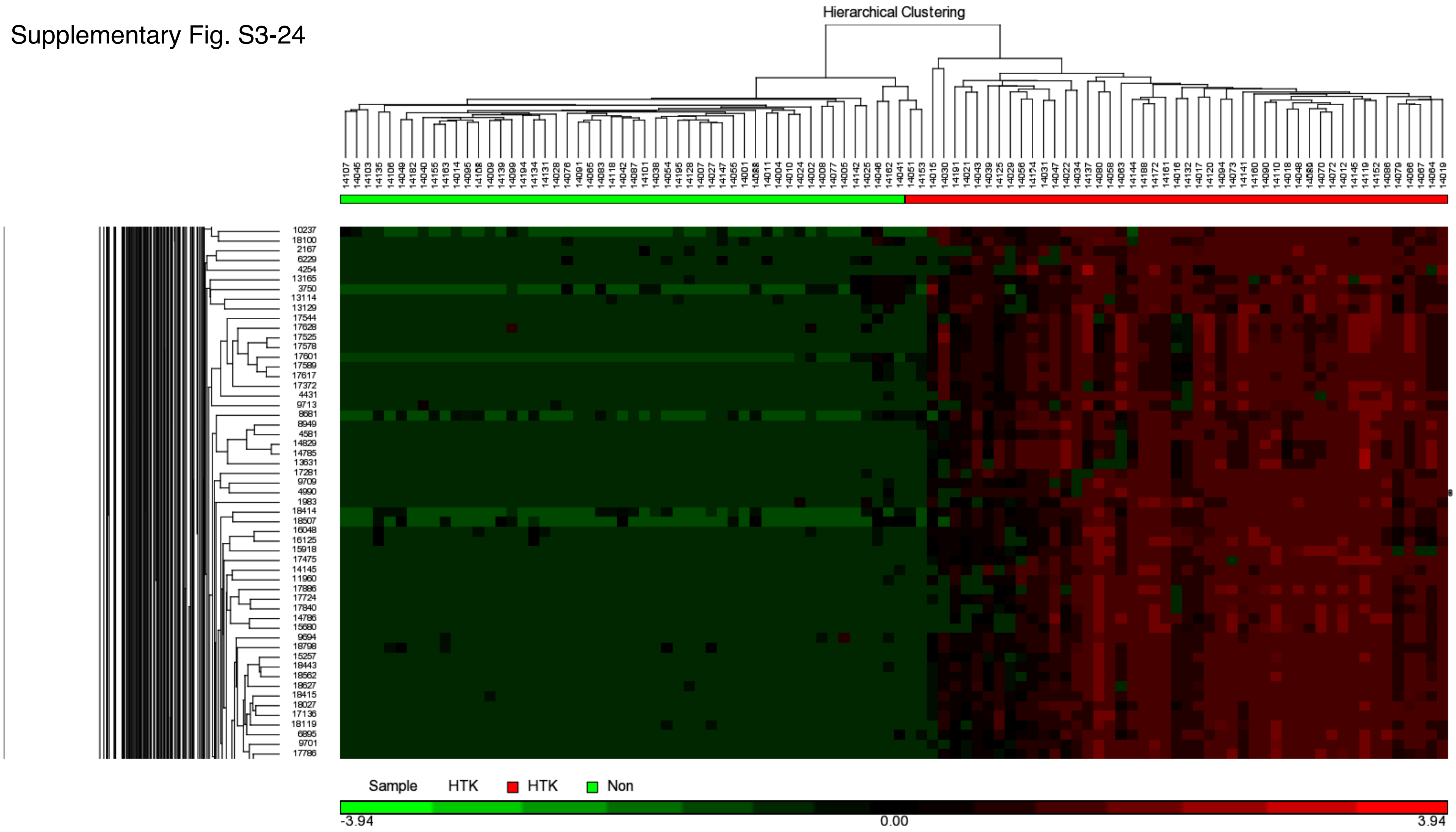

Supplementary Fig. S3-25

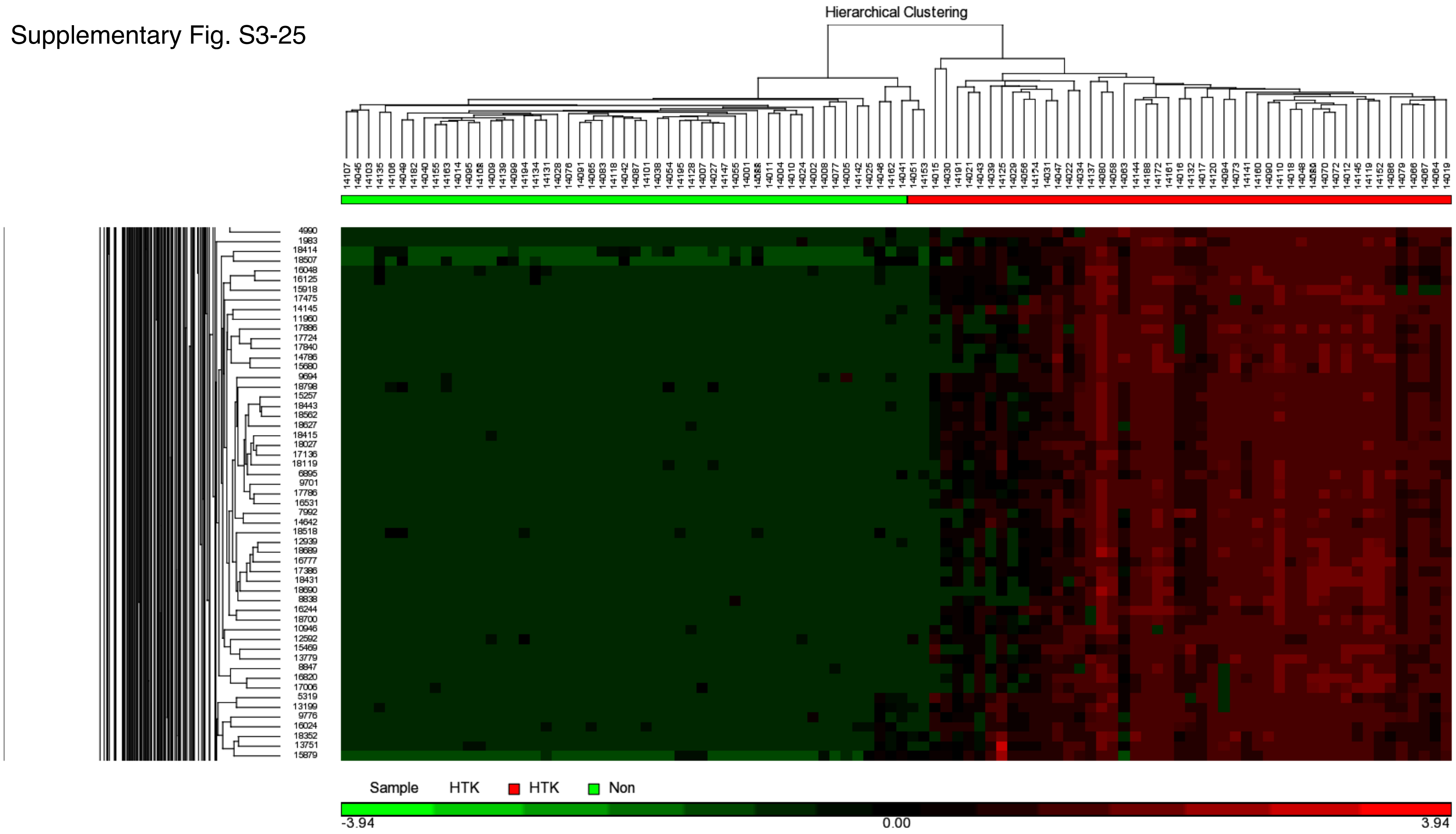

Supplementary Fig. S3-26

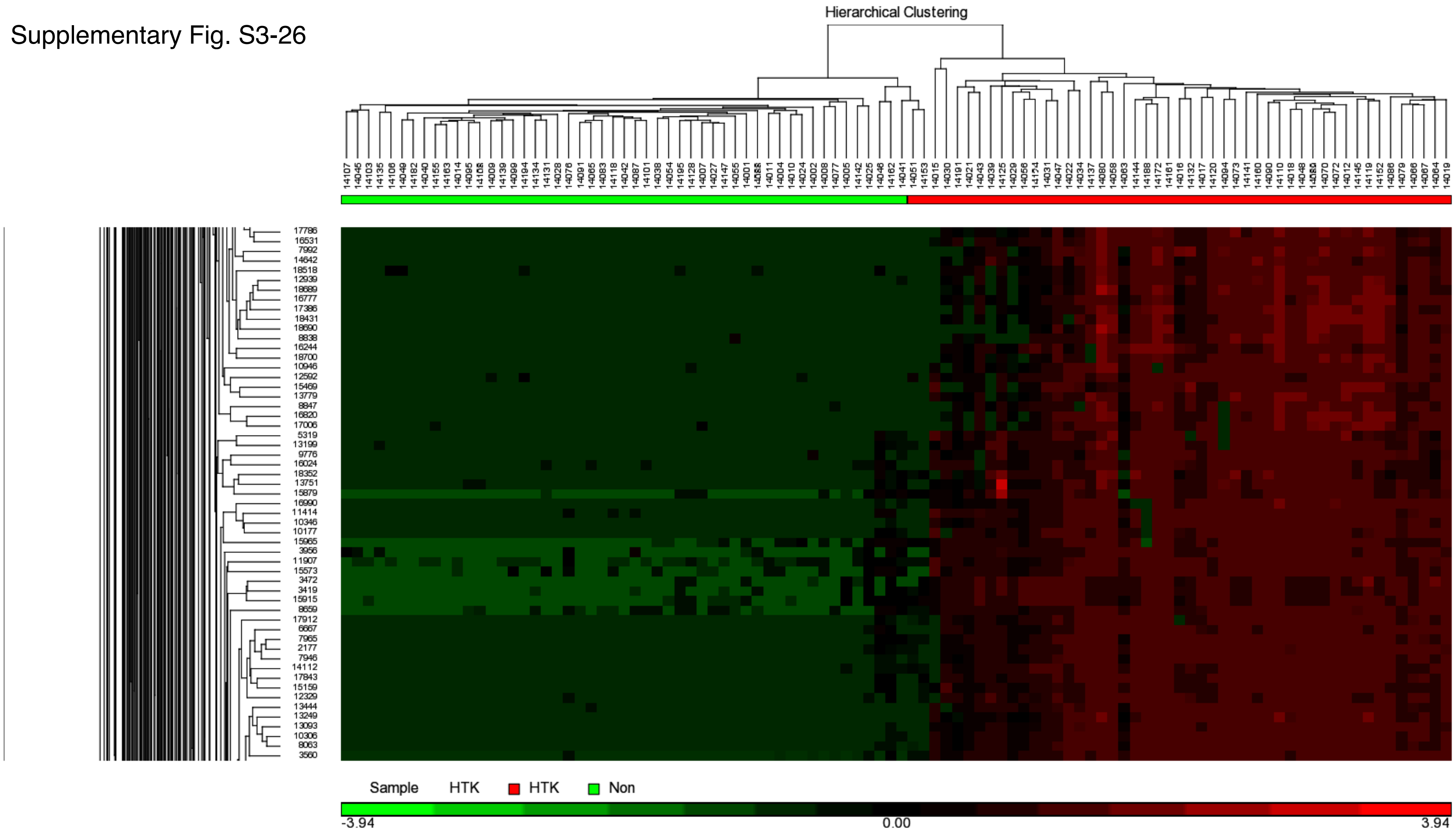

Supplementary Fig. S3-27

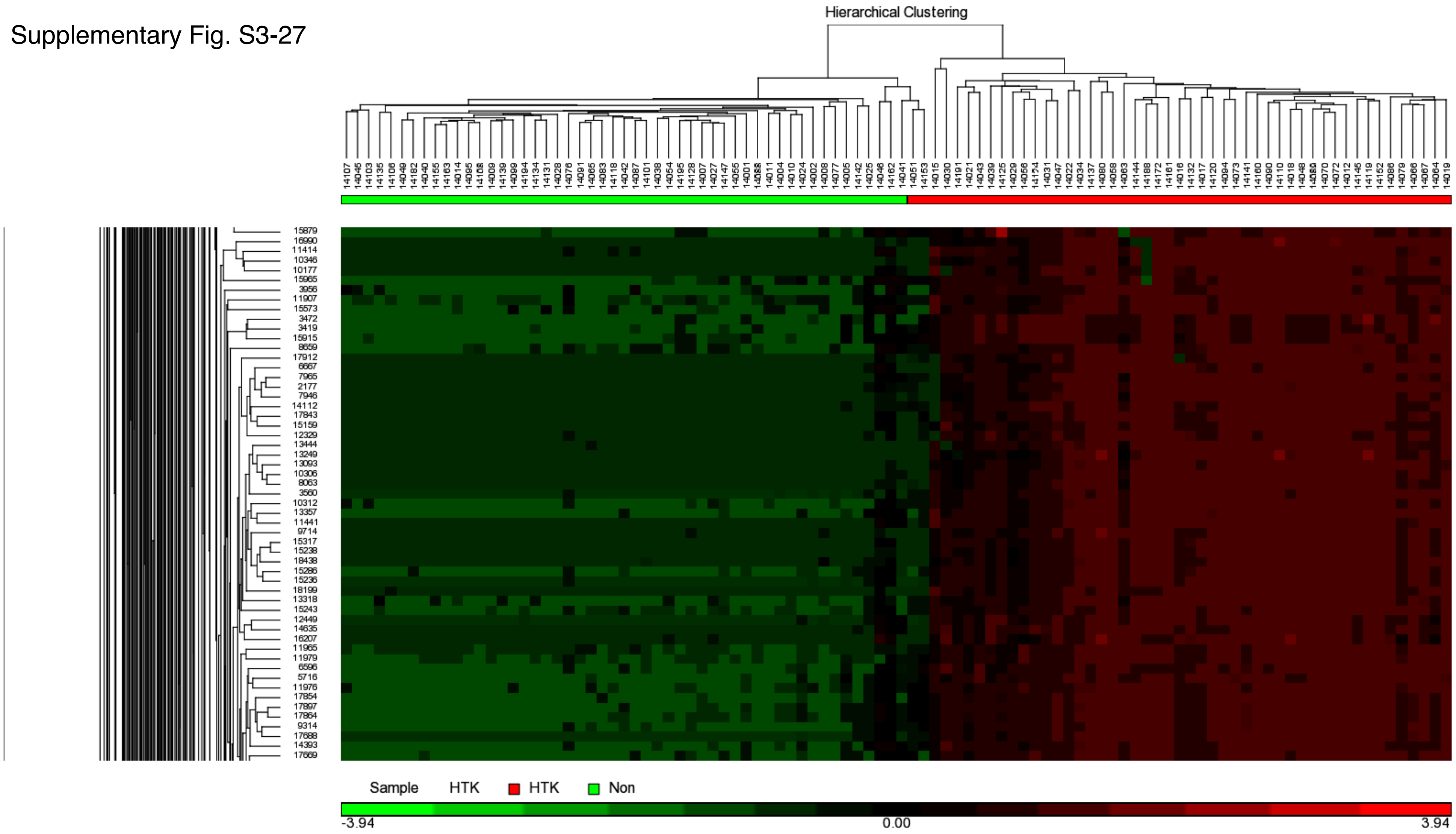

Supplementary Fig. S3-28

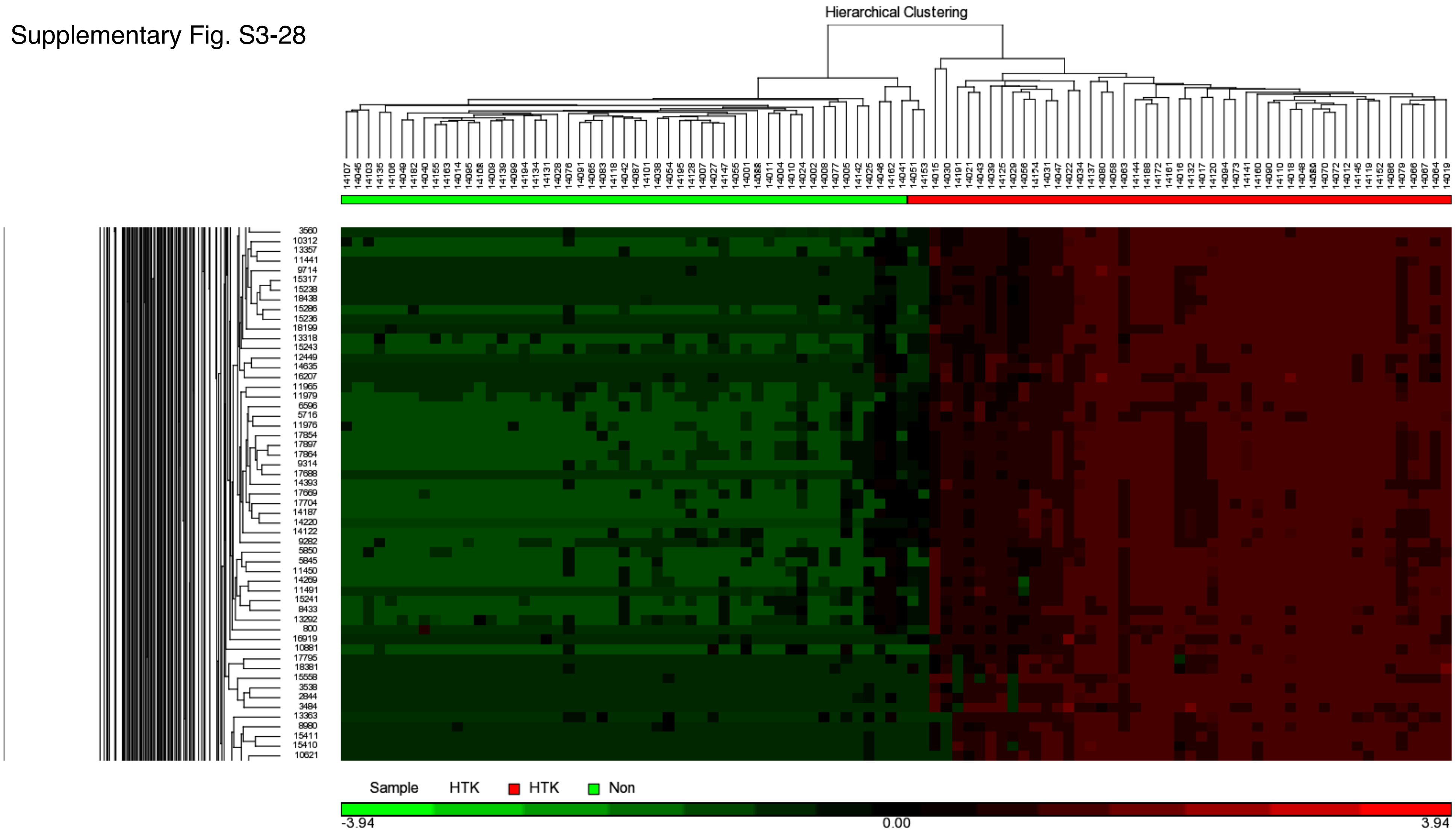

Supplementary Fig. S3-29

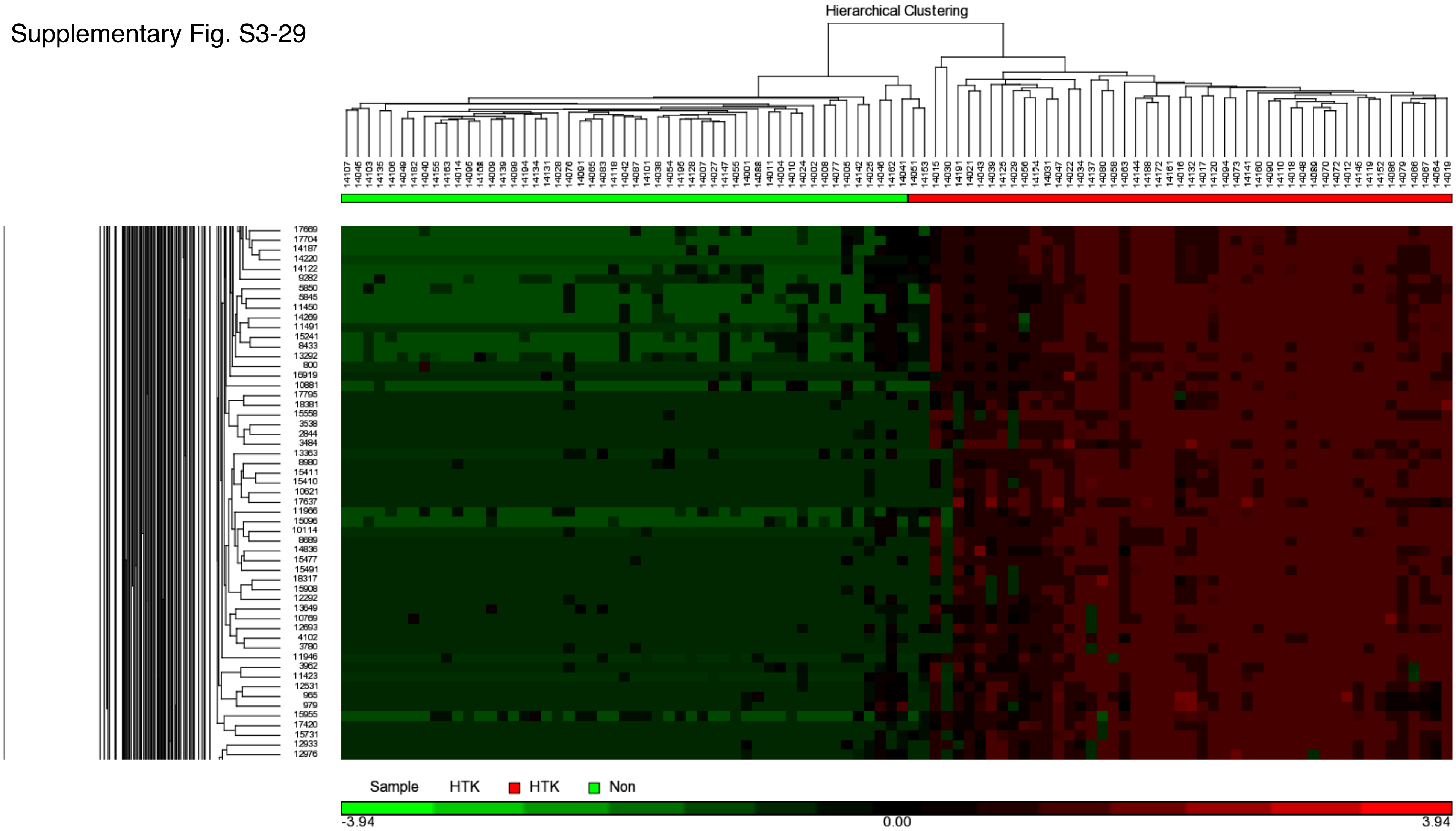

Supplementary Fig. S3-30

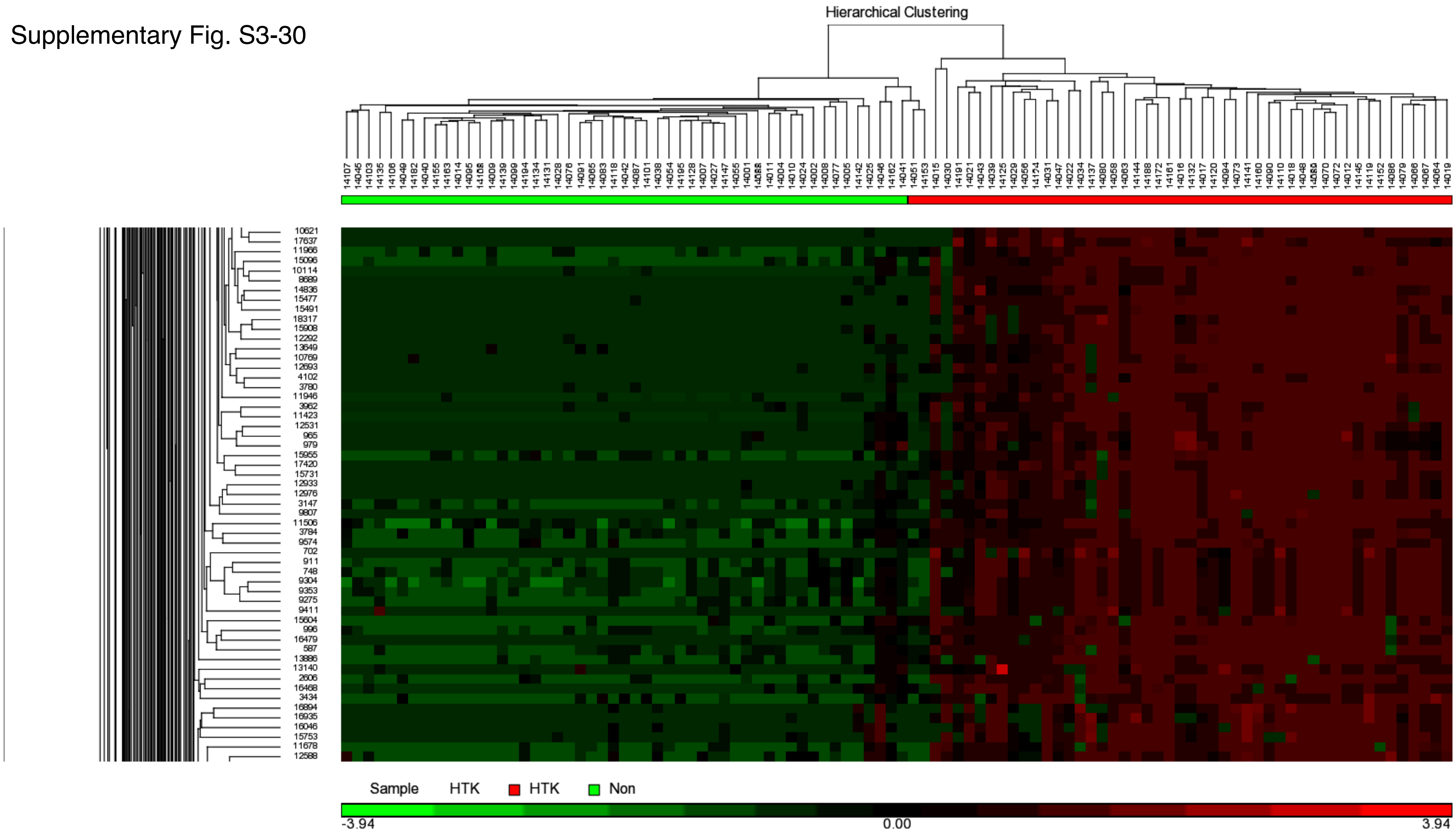

Supplementary Fig. S3-31

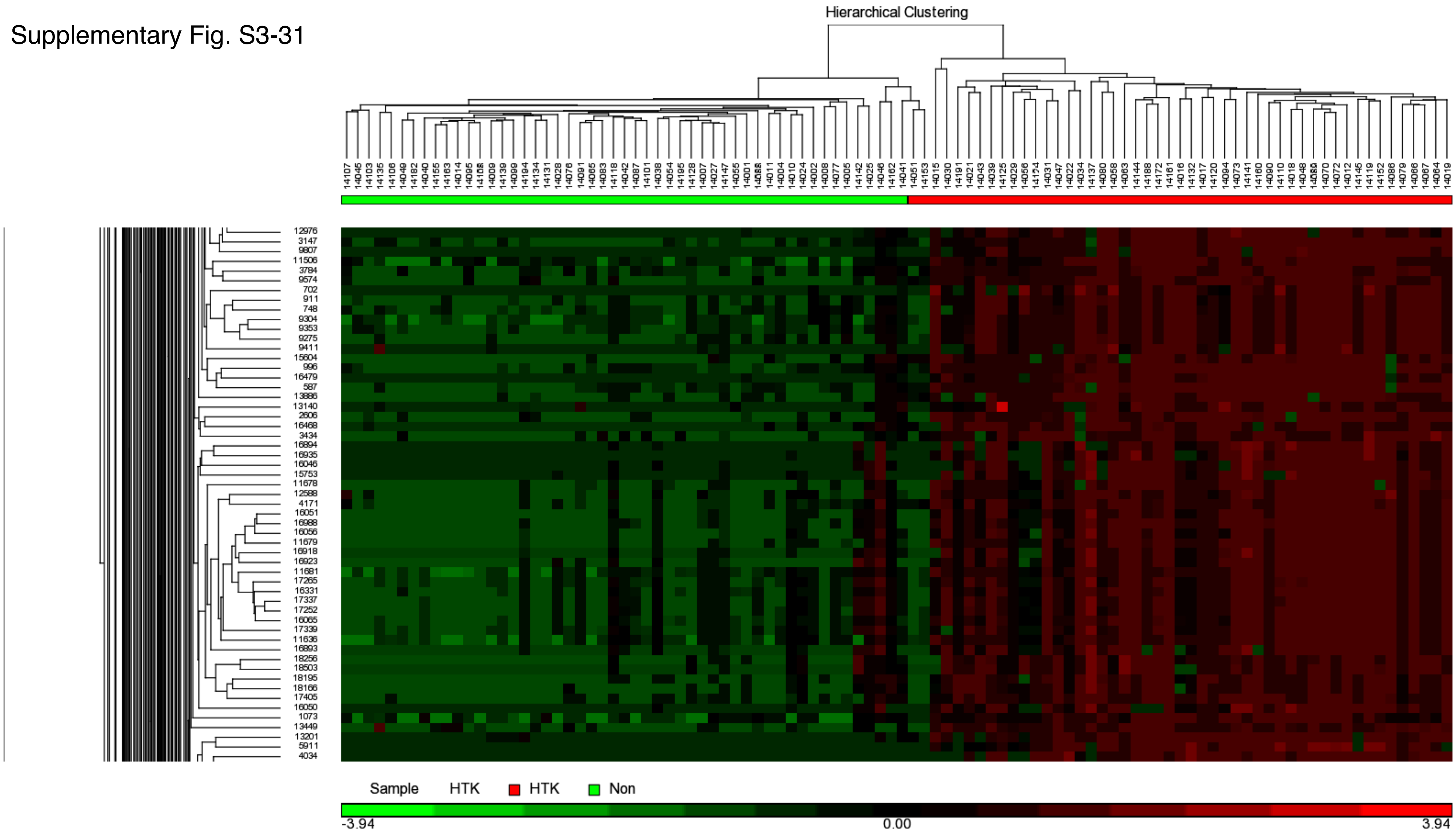

Supplementary Fig. S3-32

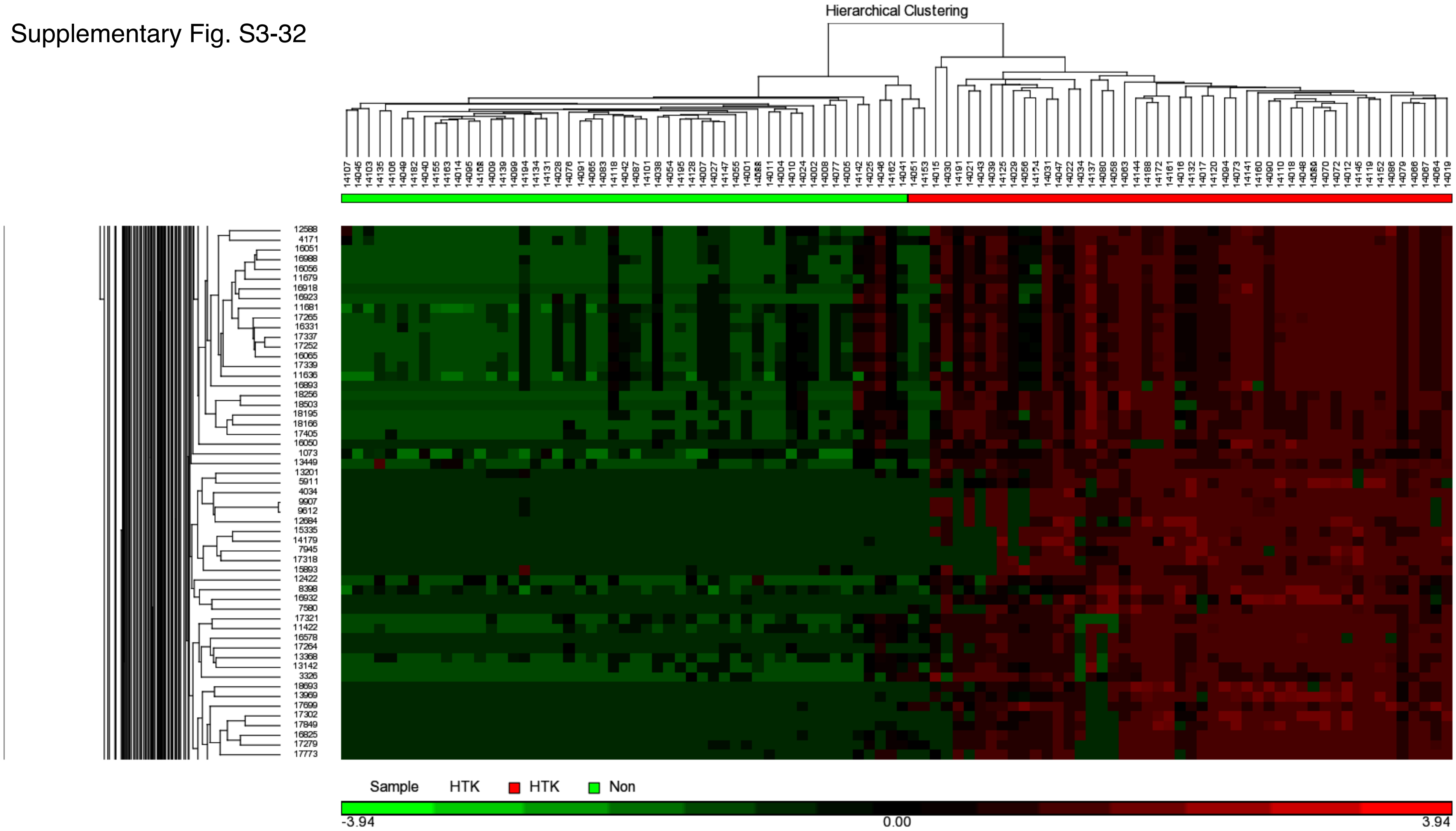

Supplementary Fig. S3-33

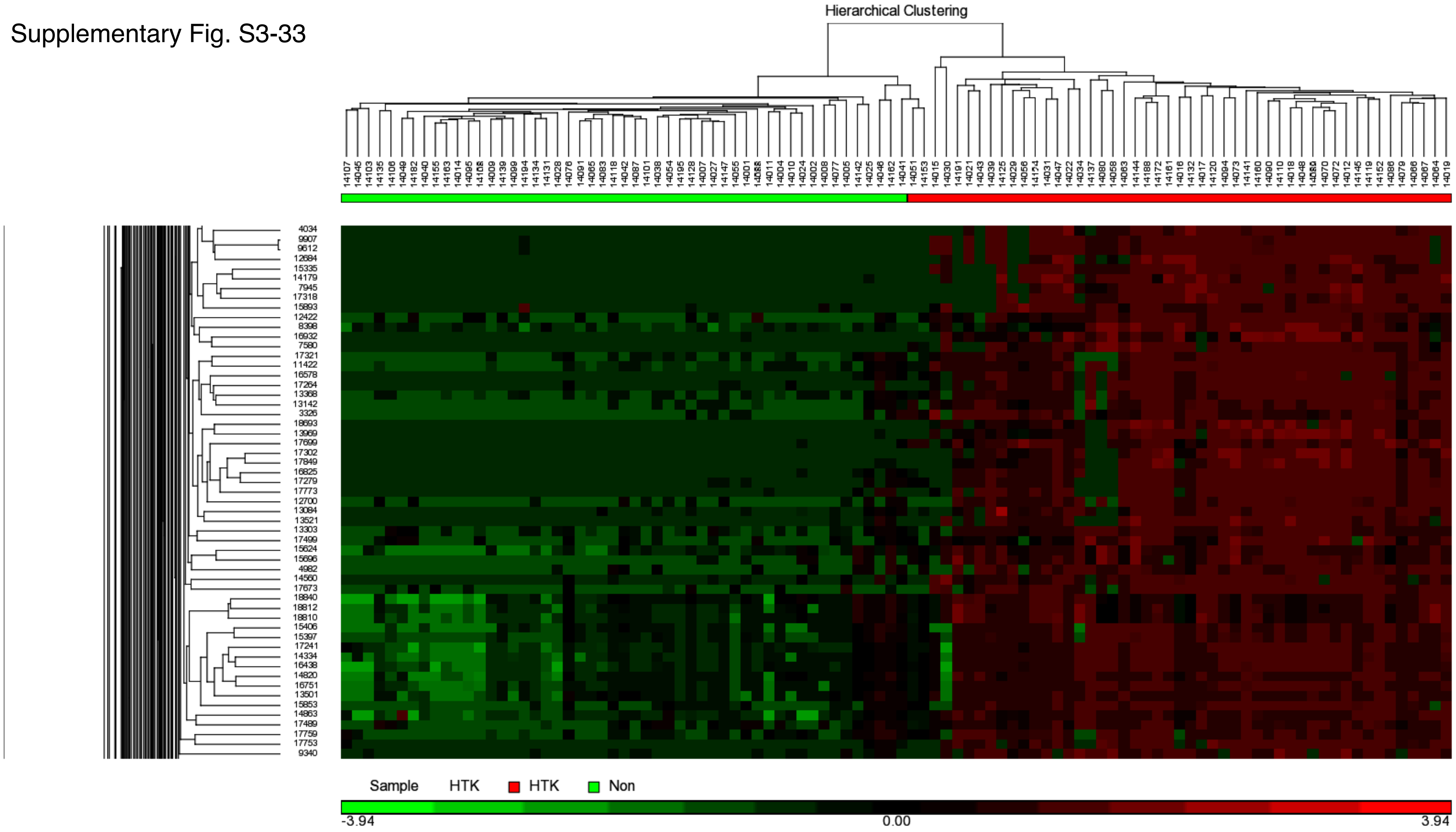

Supplementary Fig. S3-34

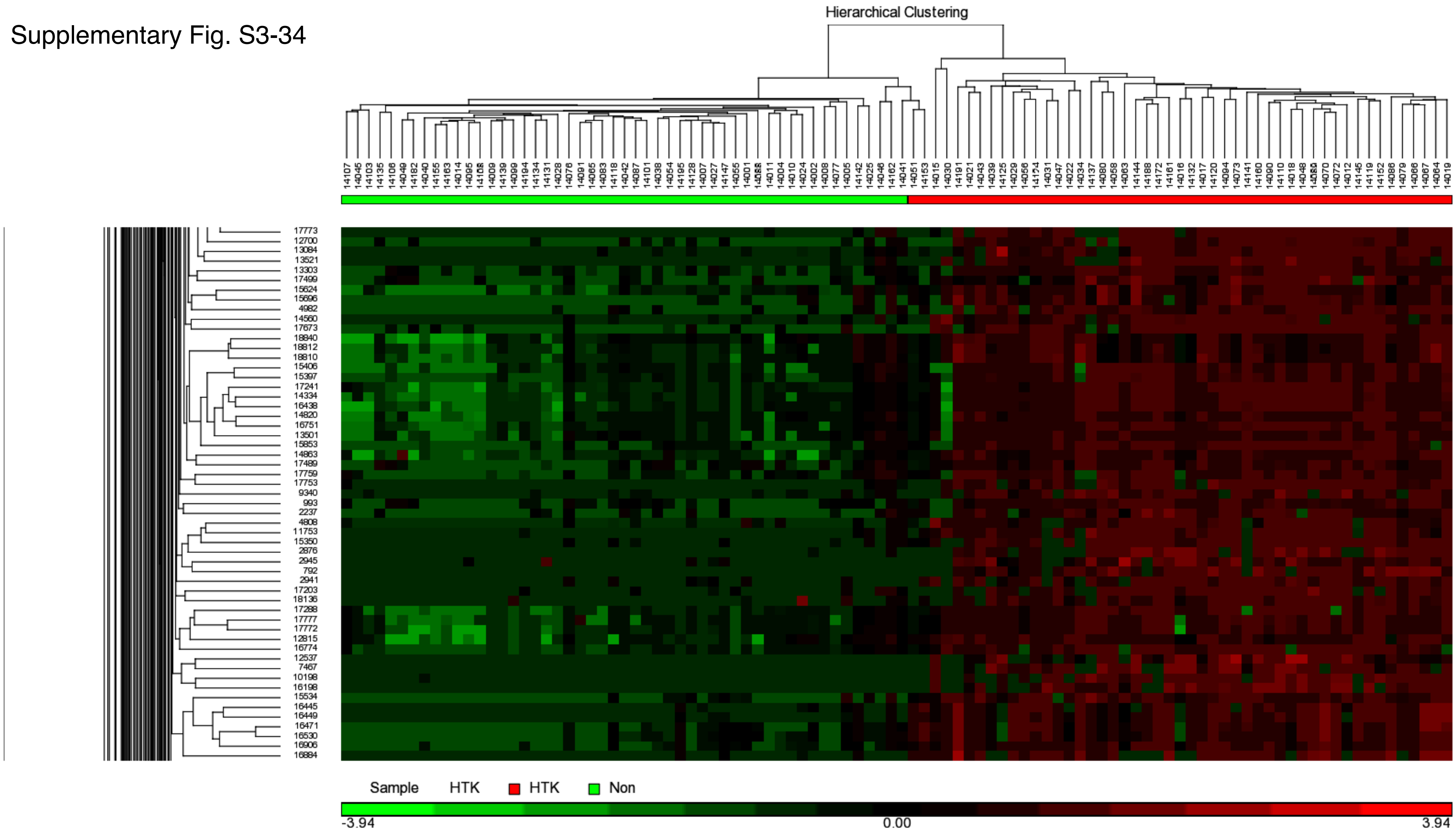

Supplementary Fig. S3-35

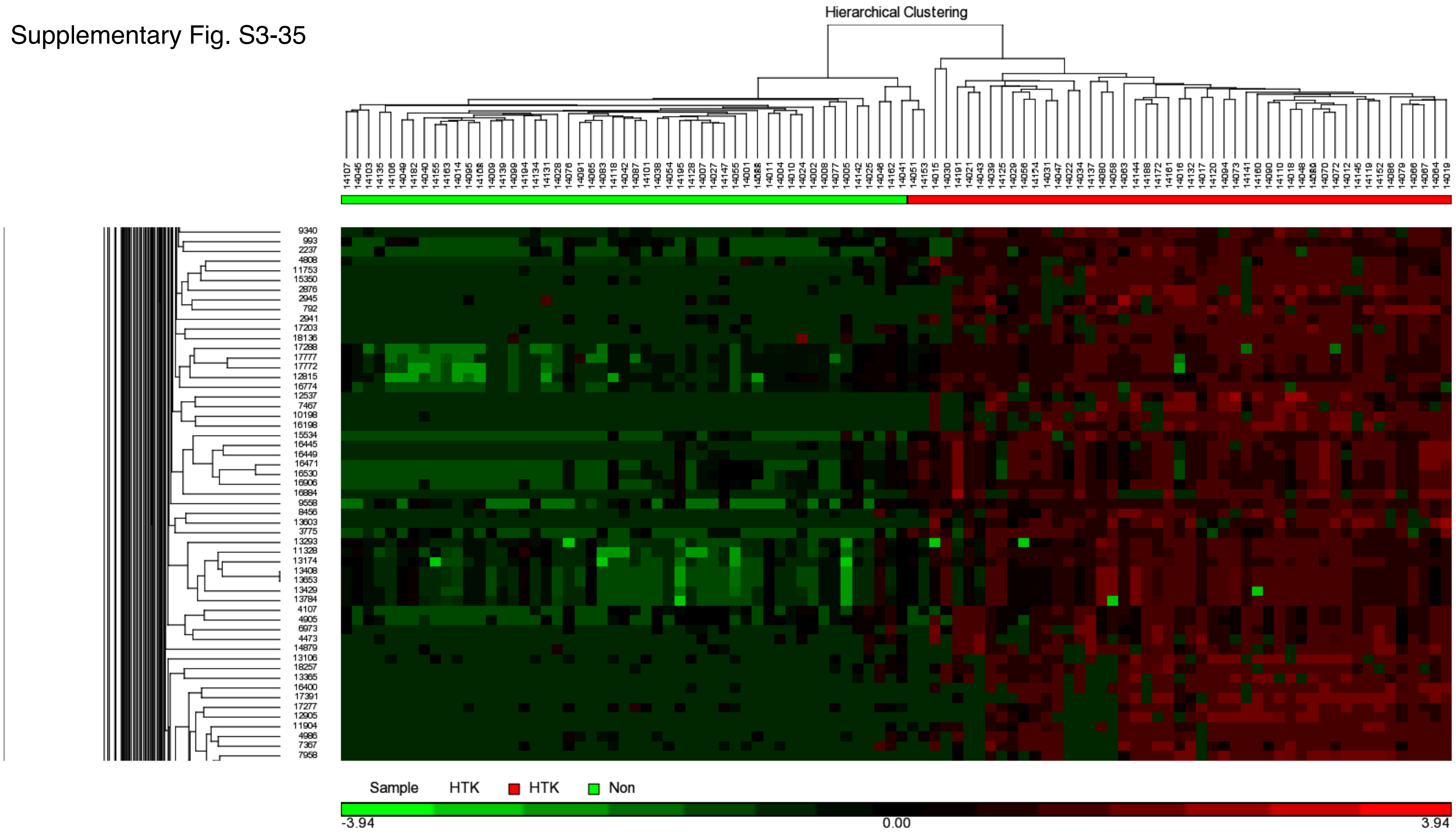

Supplementary Fig. S3-36

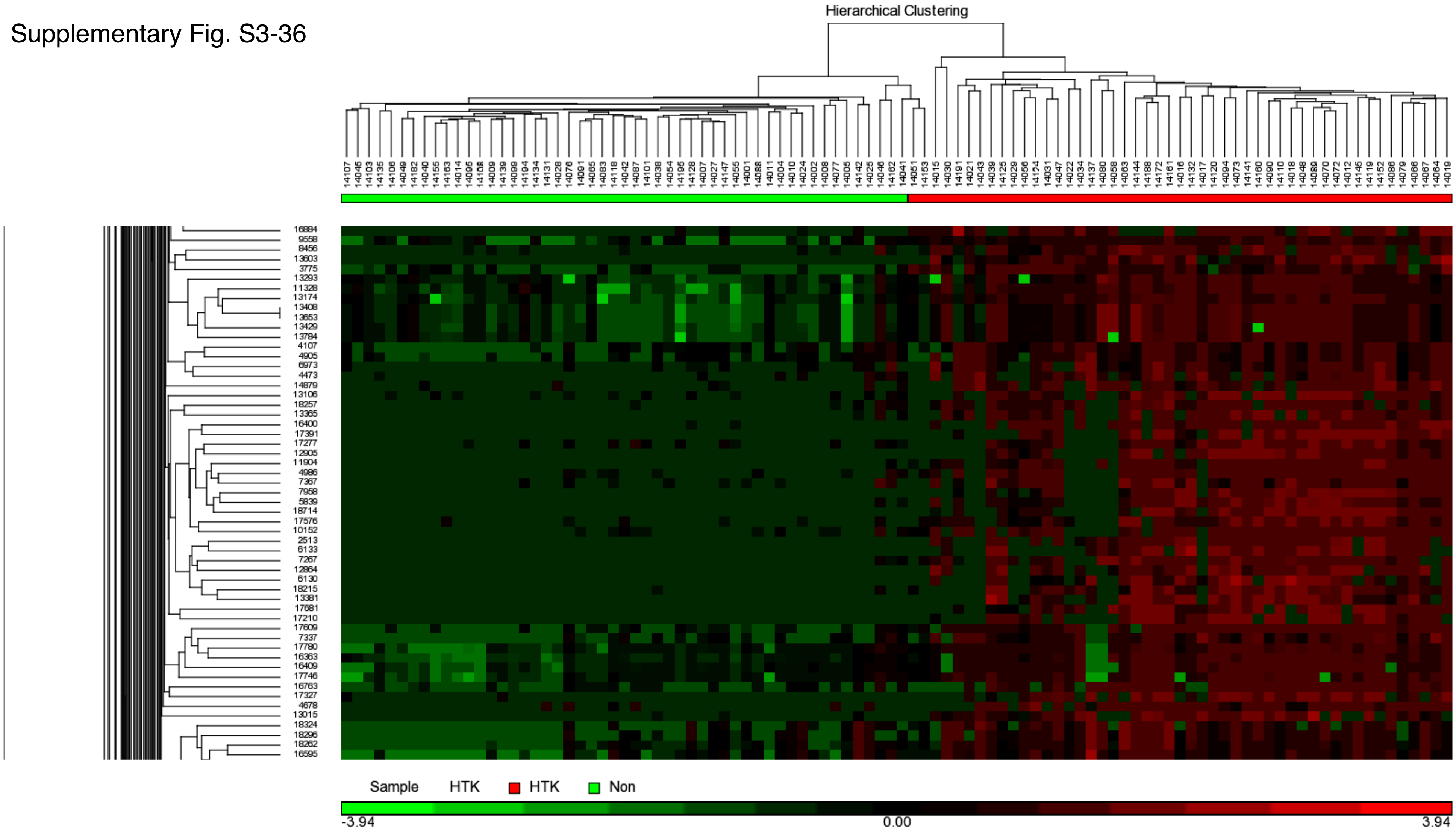

Supplementary Fig. S3-37

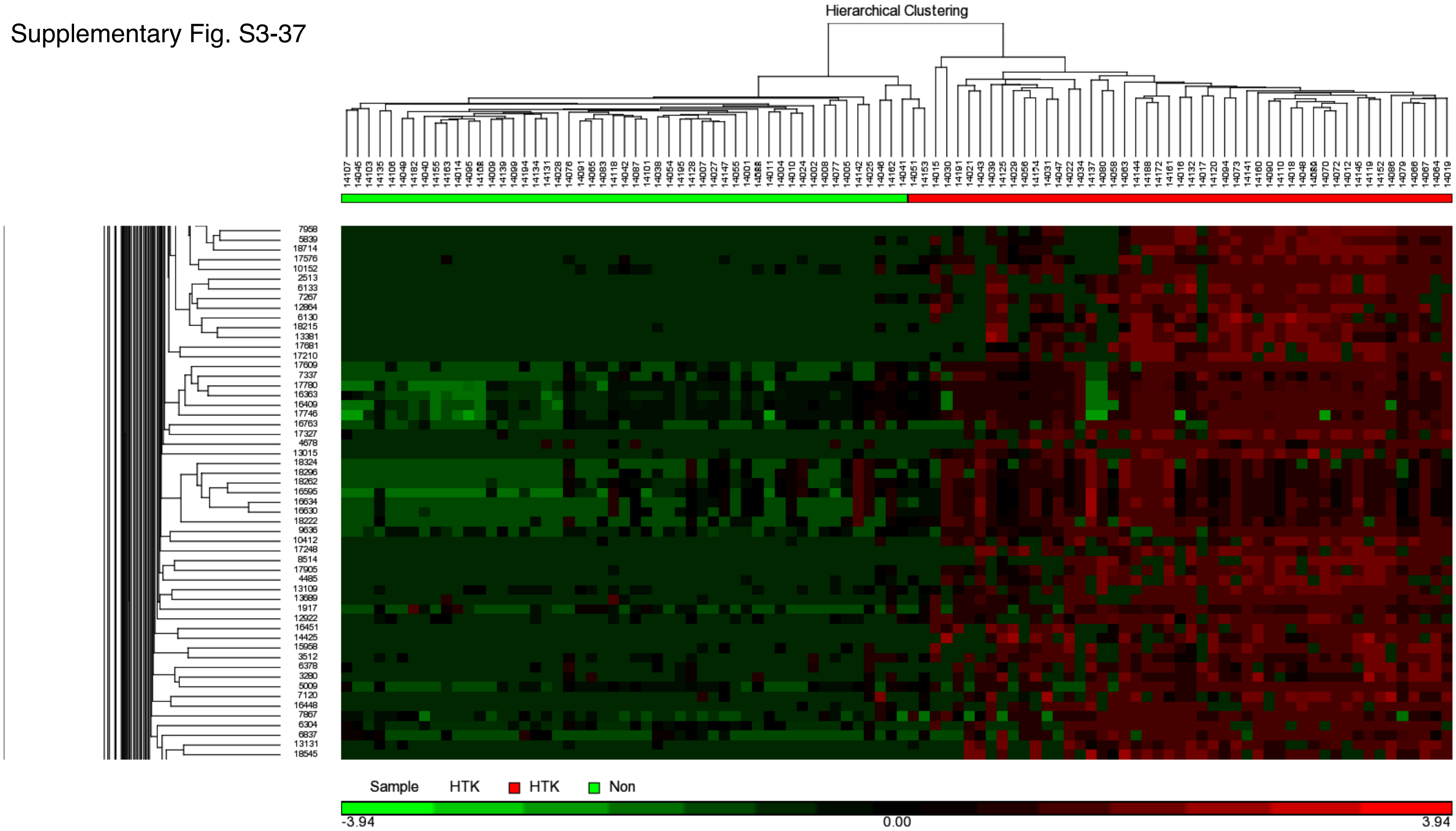

Supplementary Fig. S3-38

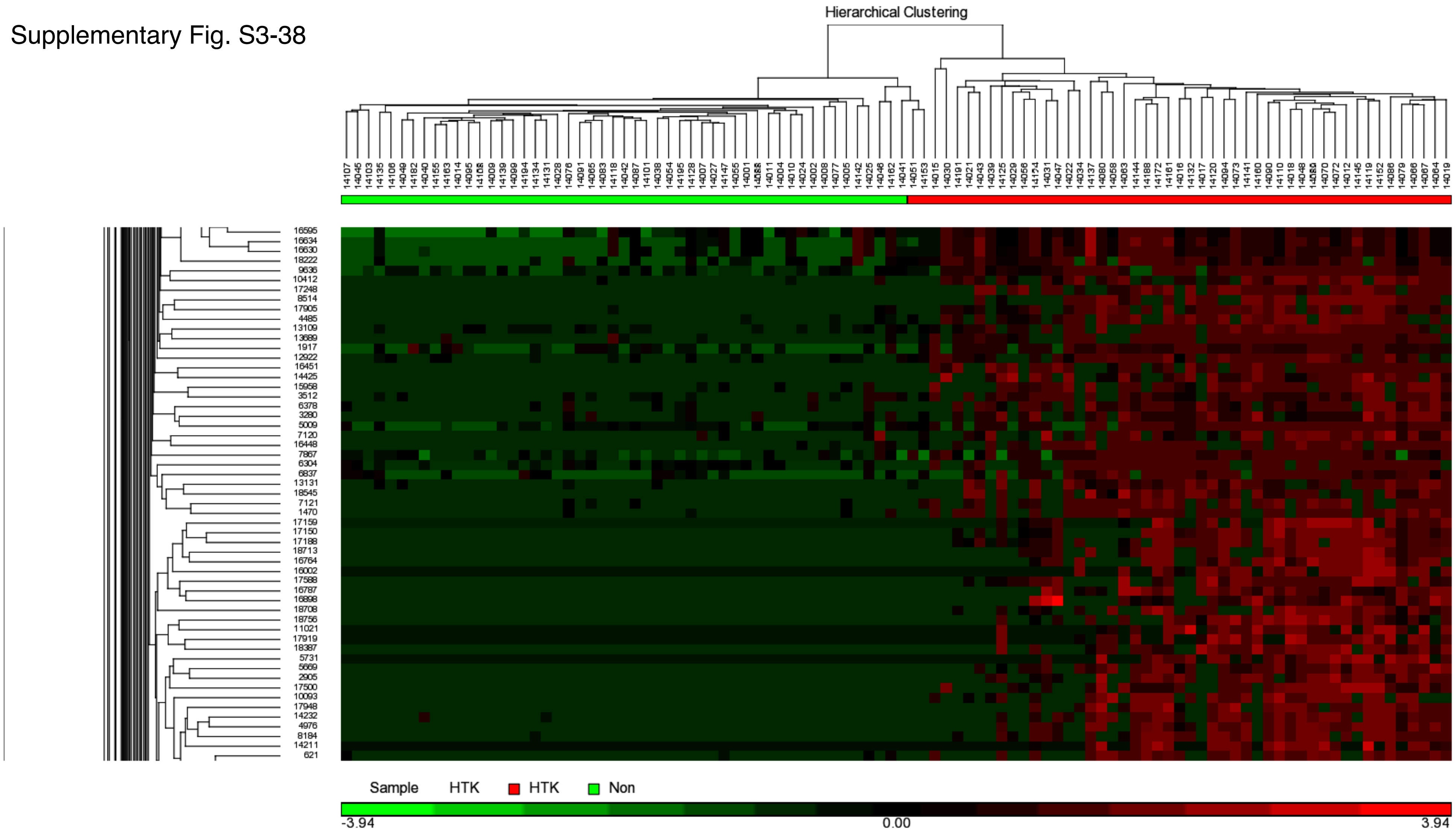

Supplementary Fig. S3-39

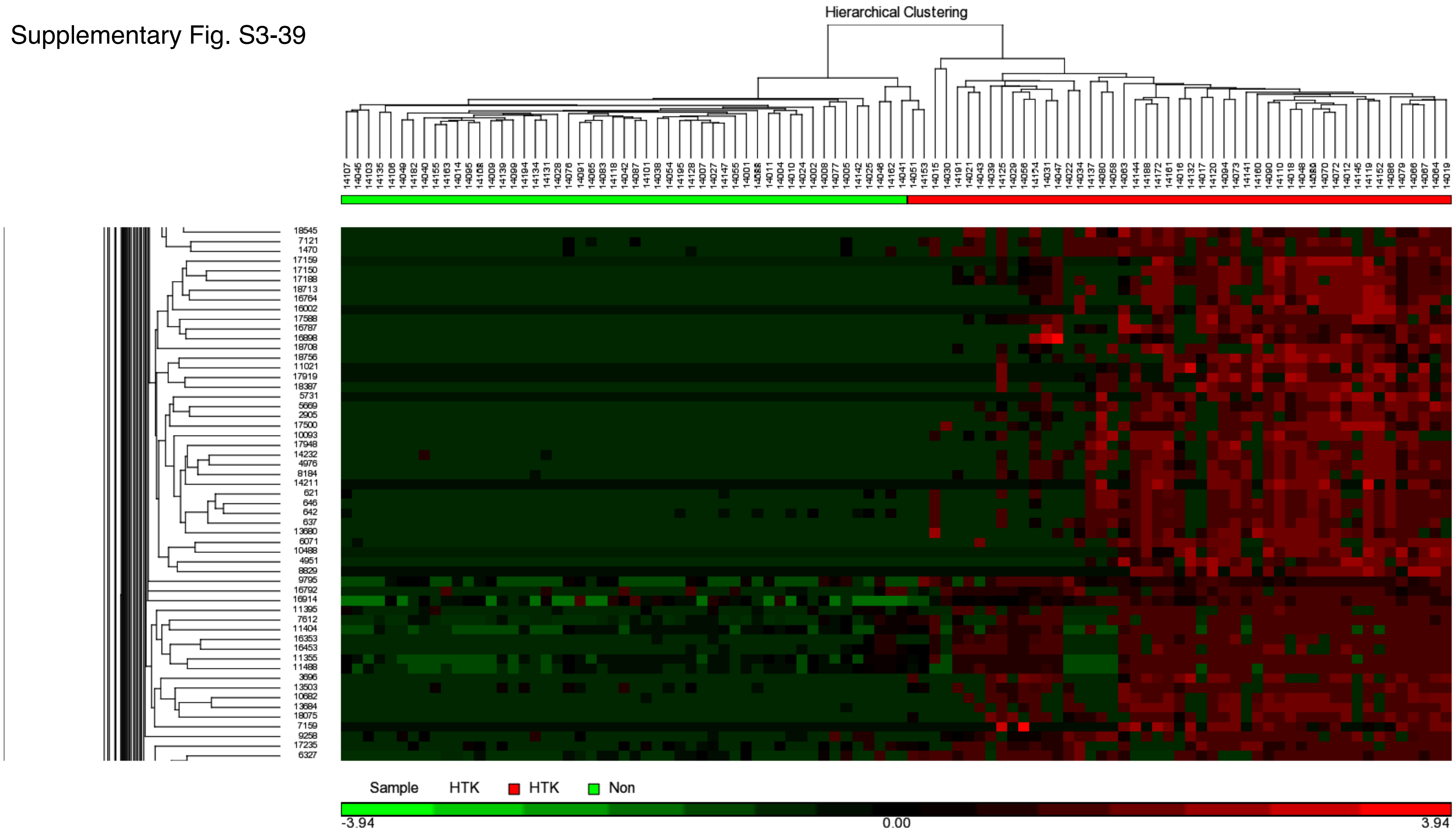

Supplementary Fig. S3-40

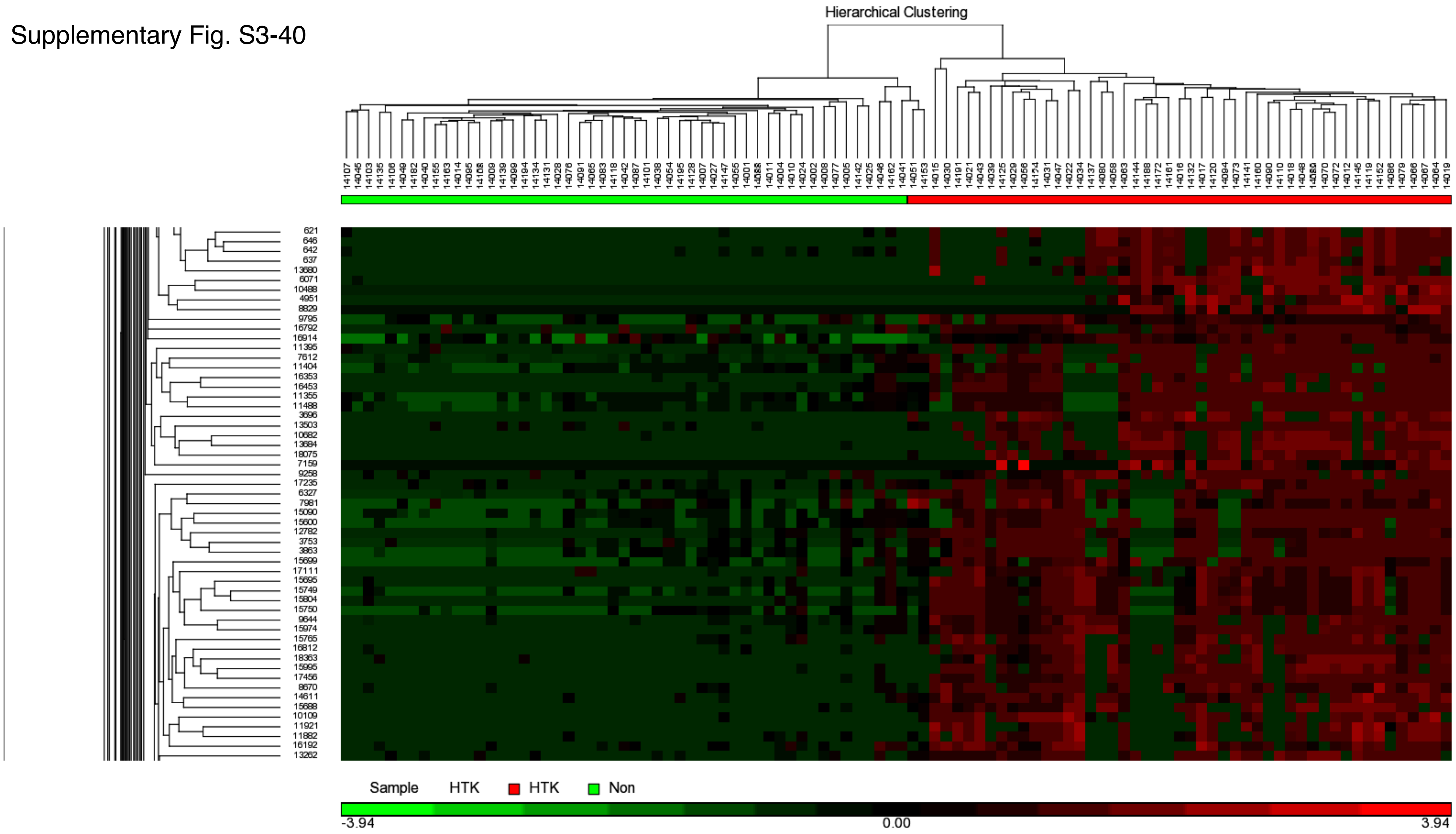

Supplementary Fig. S3-41

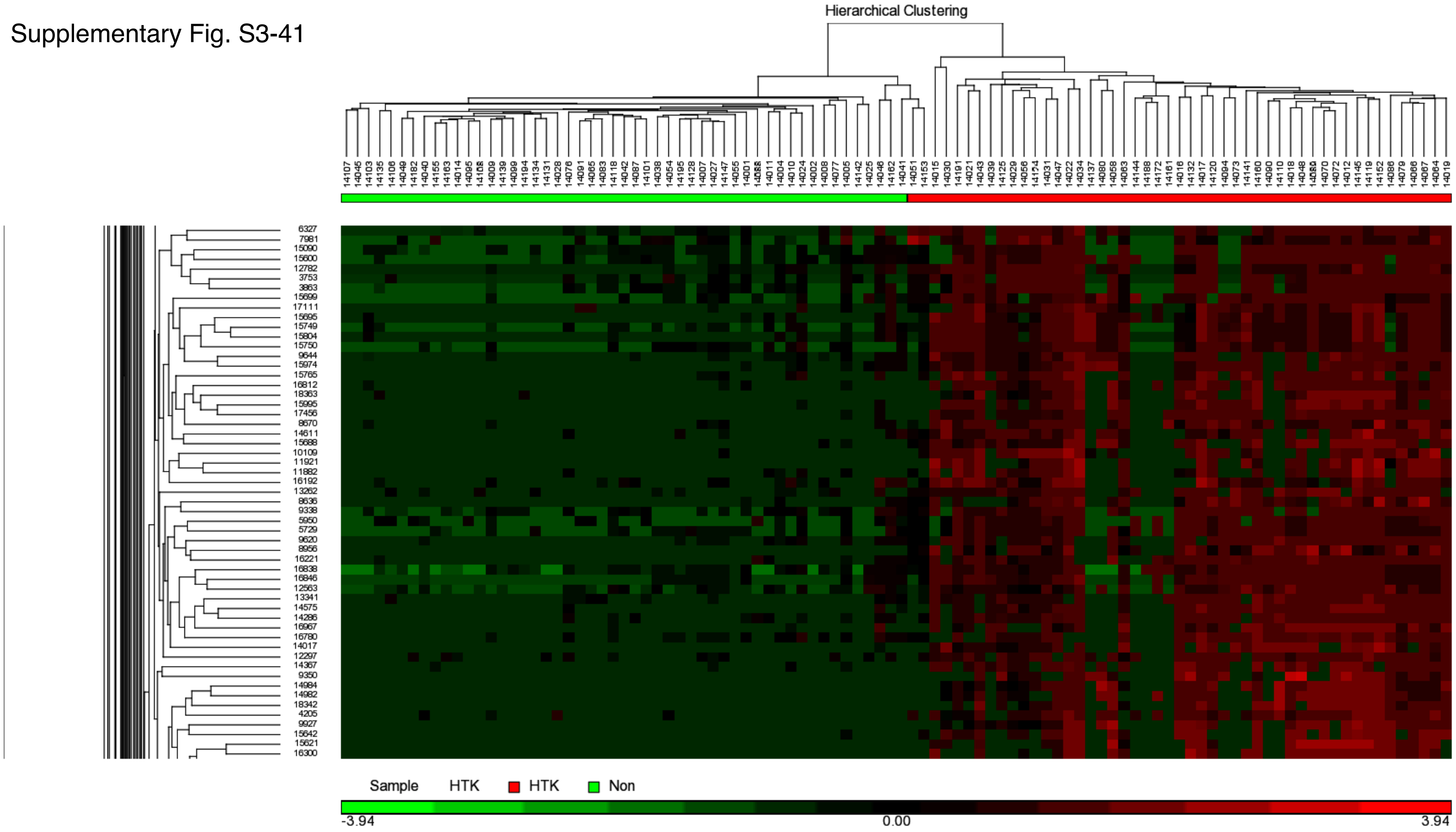

Supplementary Fig. S3-42

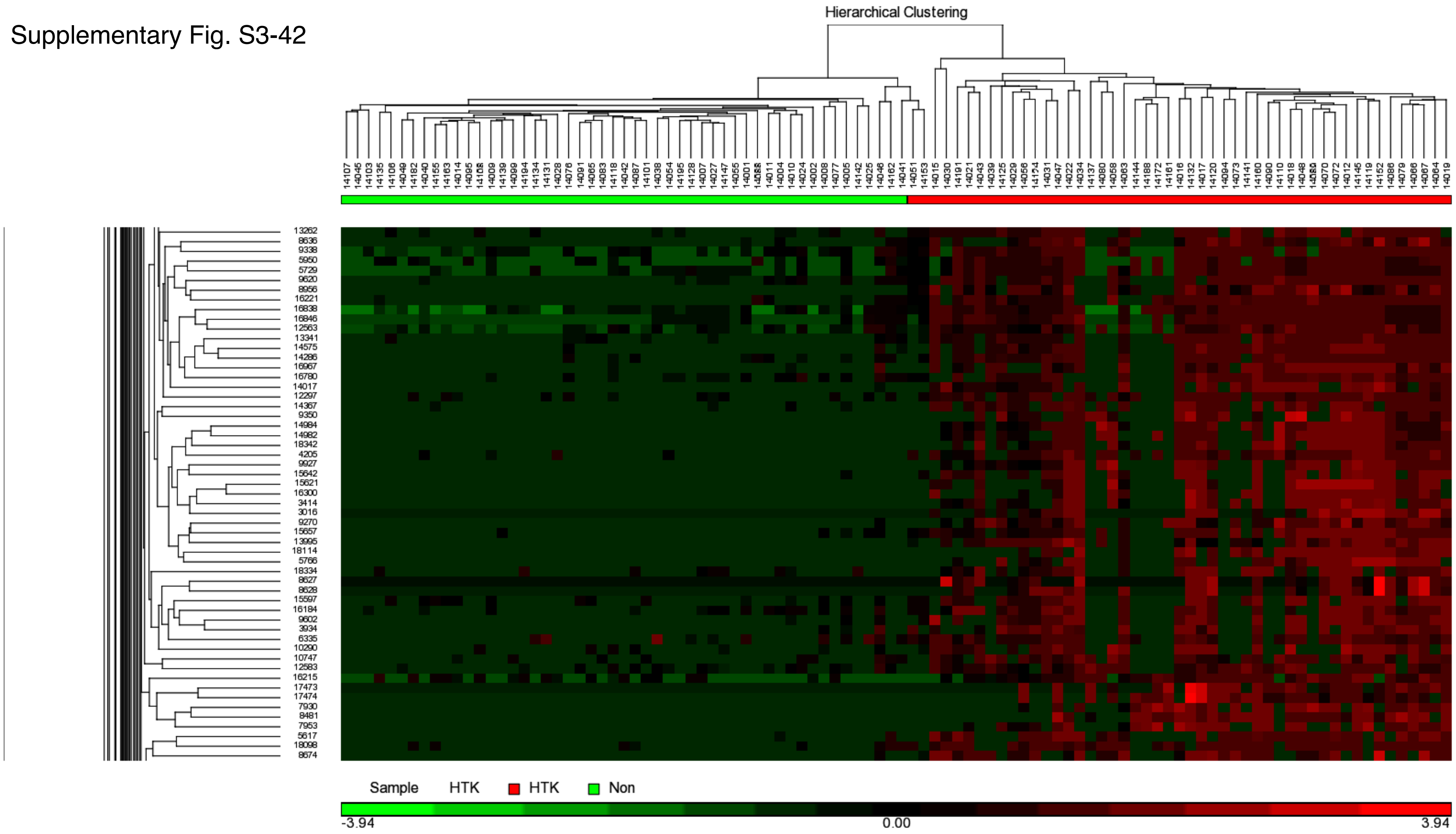

Supplementary Fig. S3-43

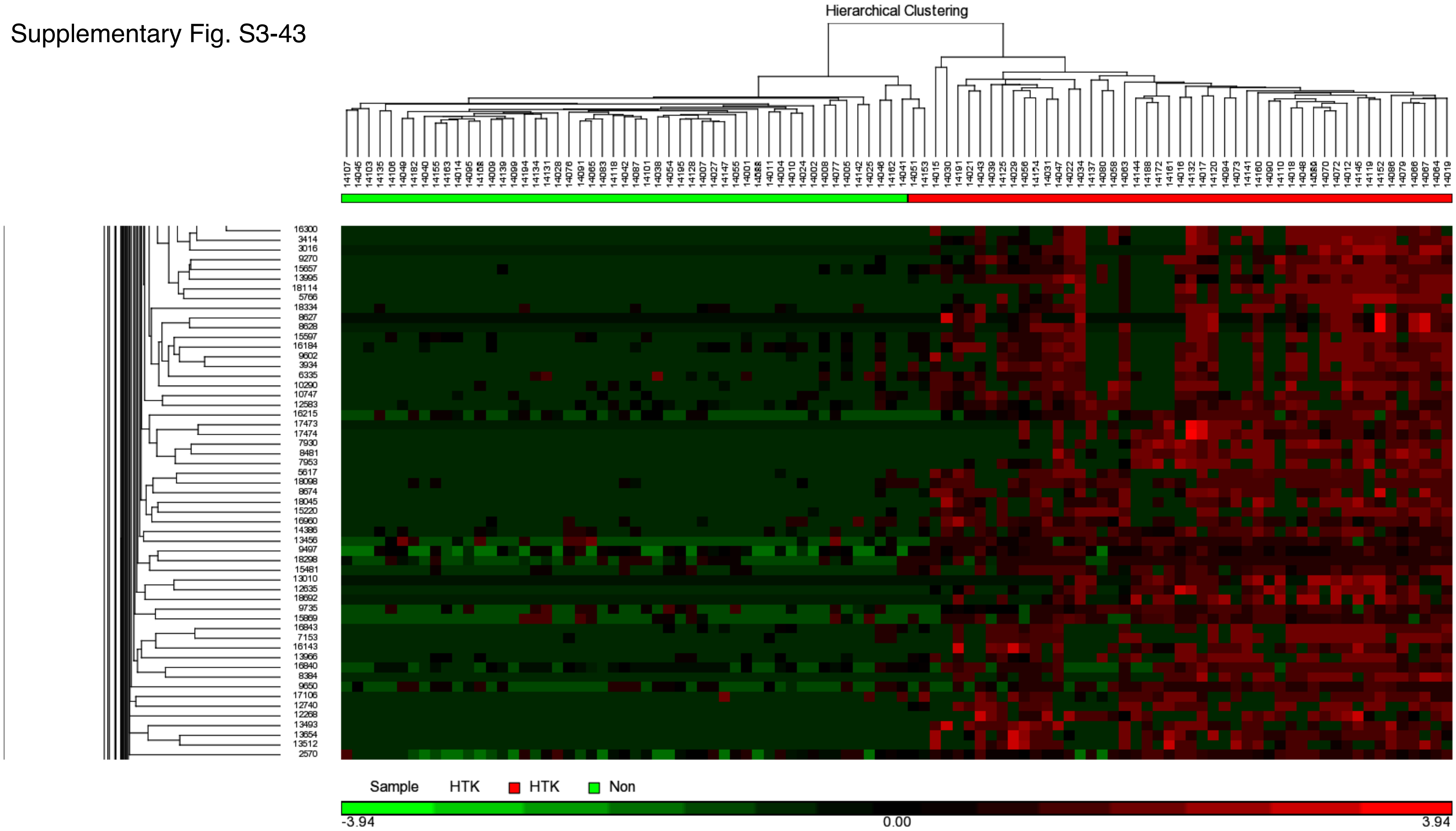

Supplementary Fig. S3-44

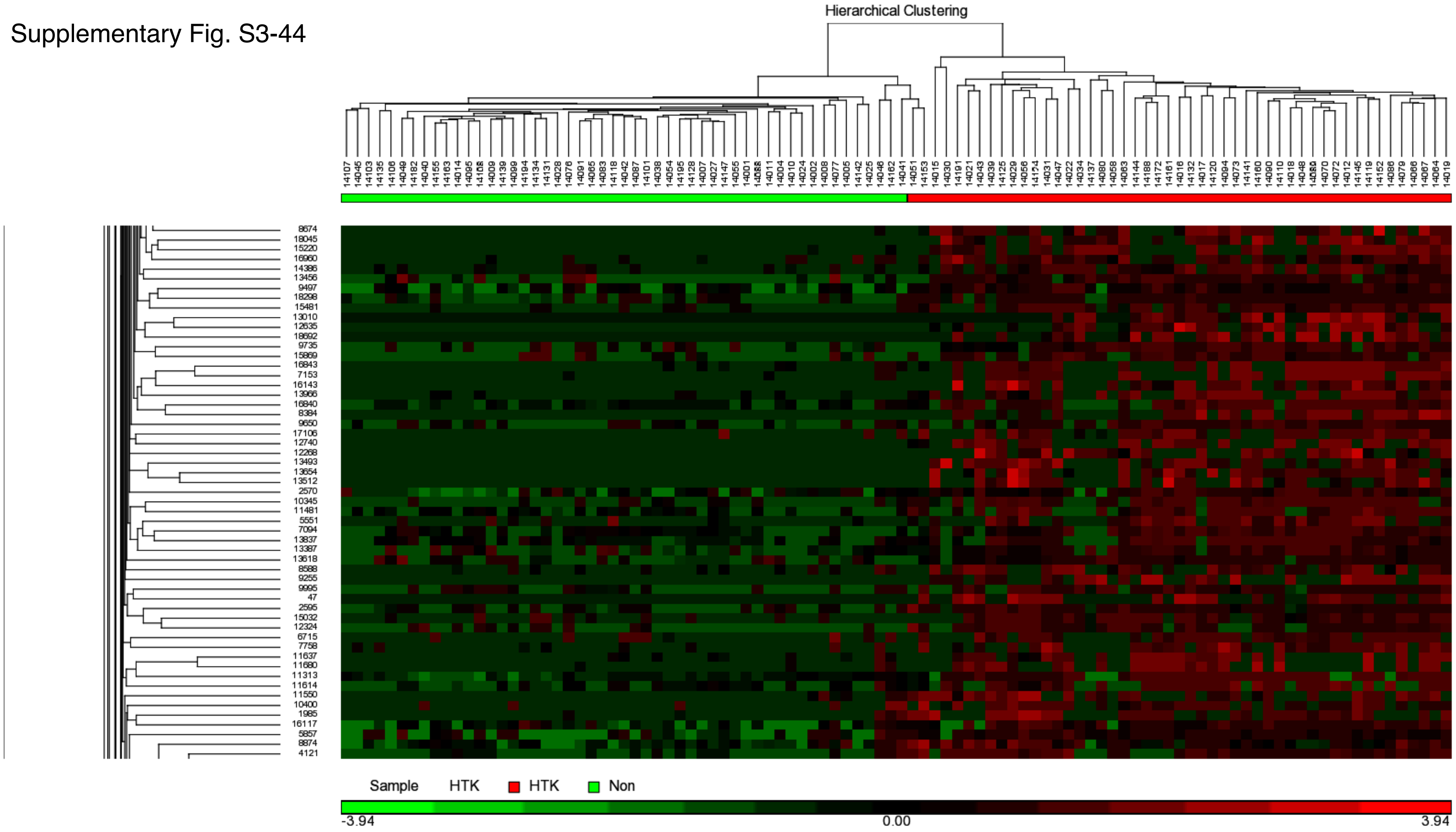

Supplementary Fig. S3-45

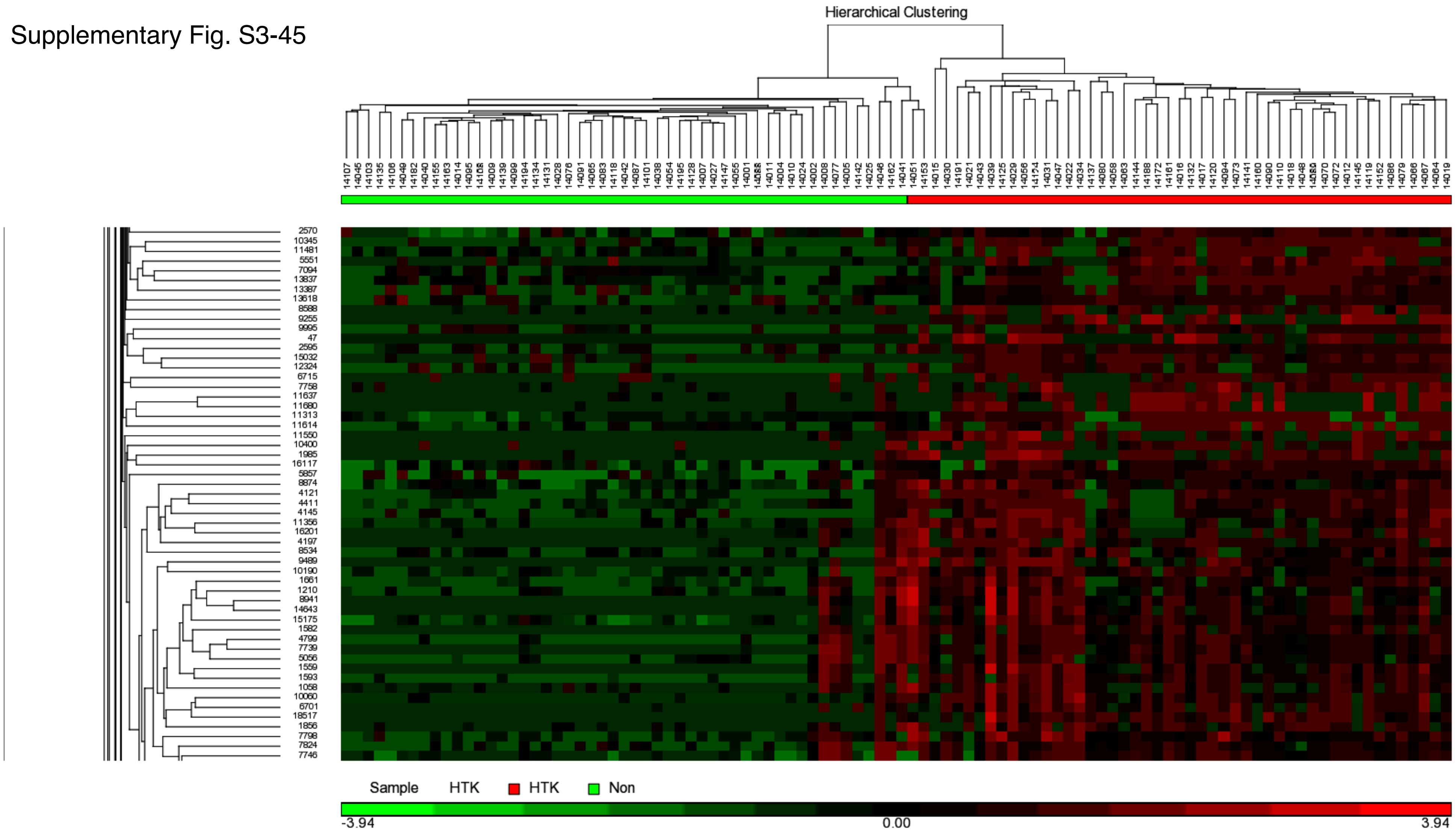

Supplementary Fig. S3-46

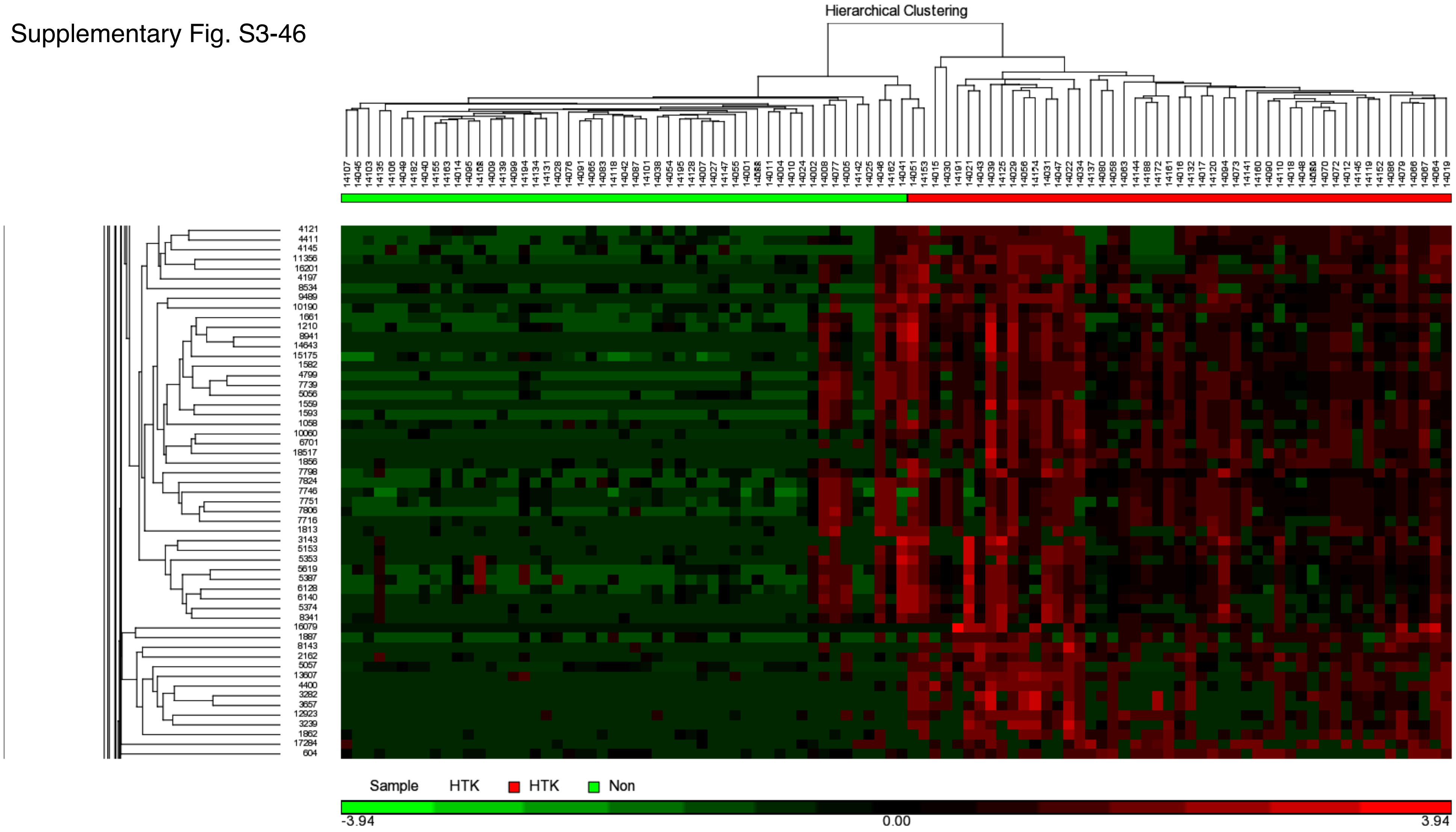

Supplementary Fig. S3-47

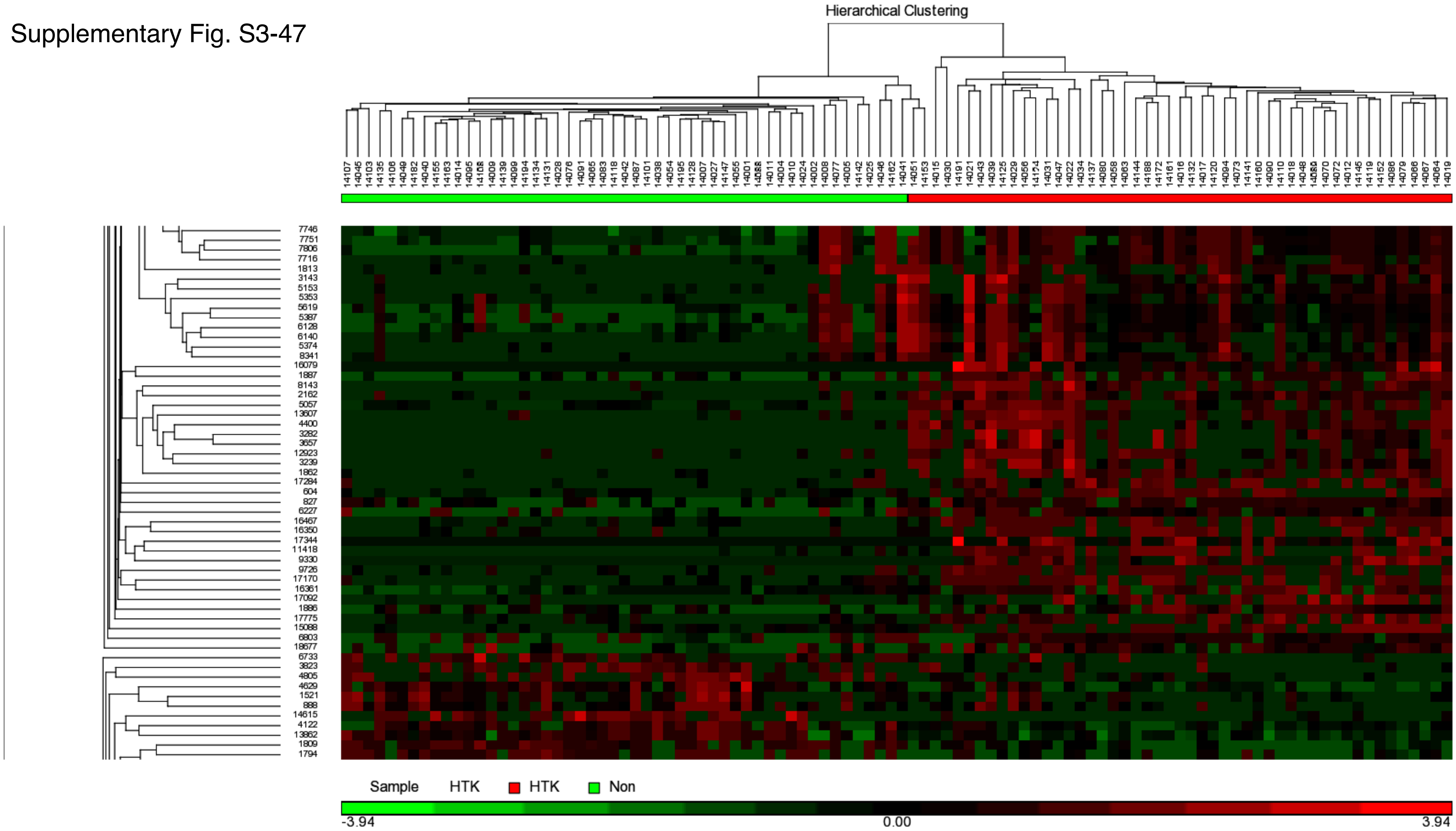

Supplementary Fig. S3-48

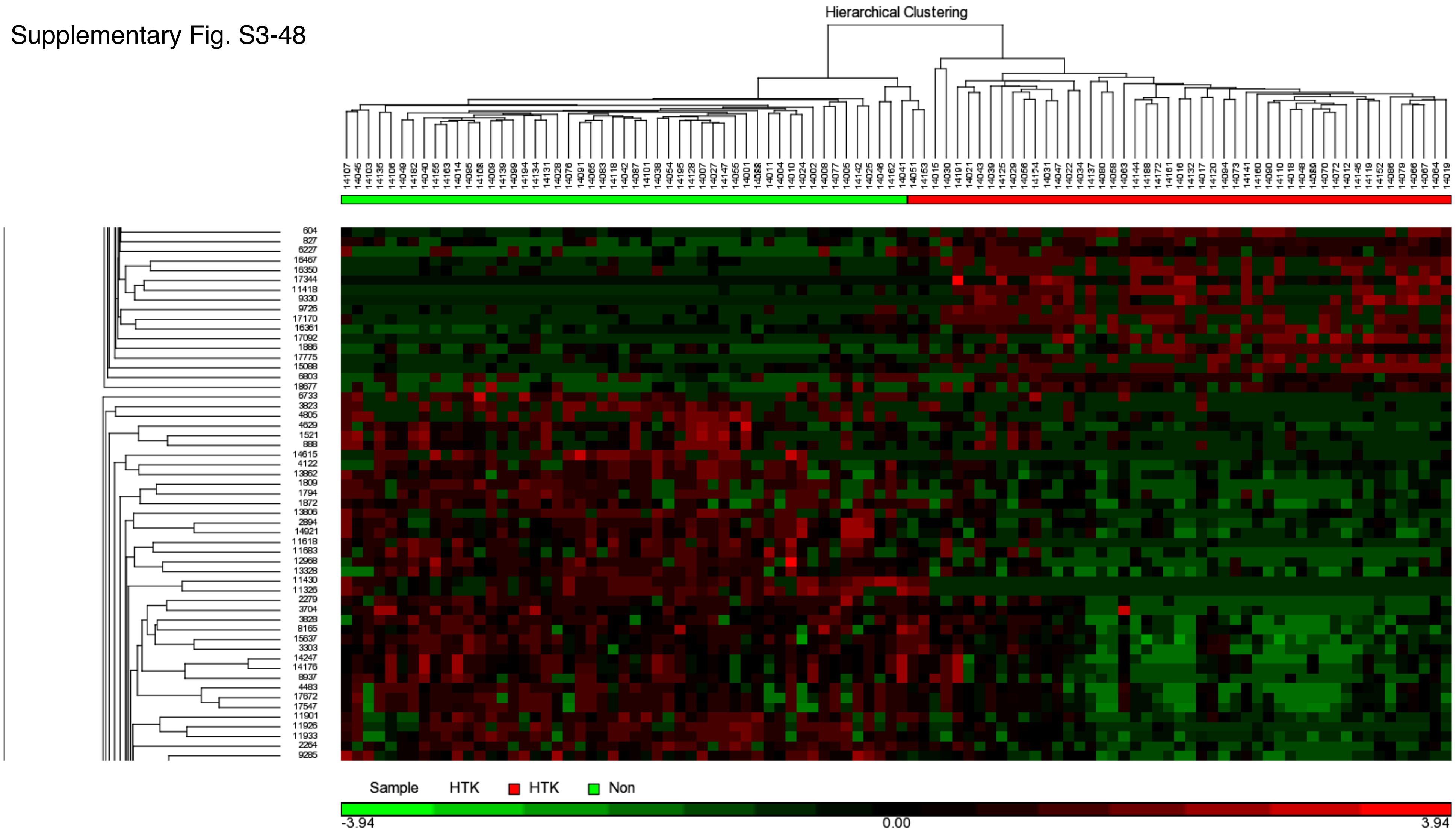

Supplementary Fig. S3-49

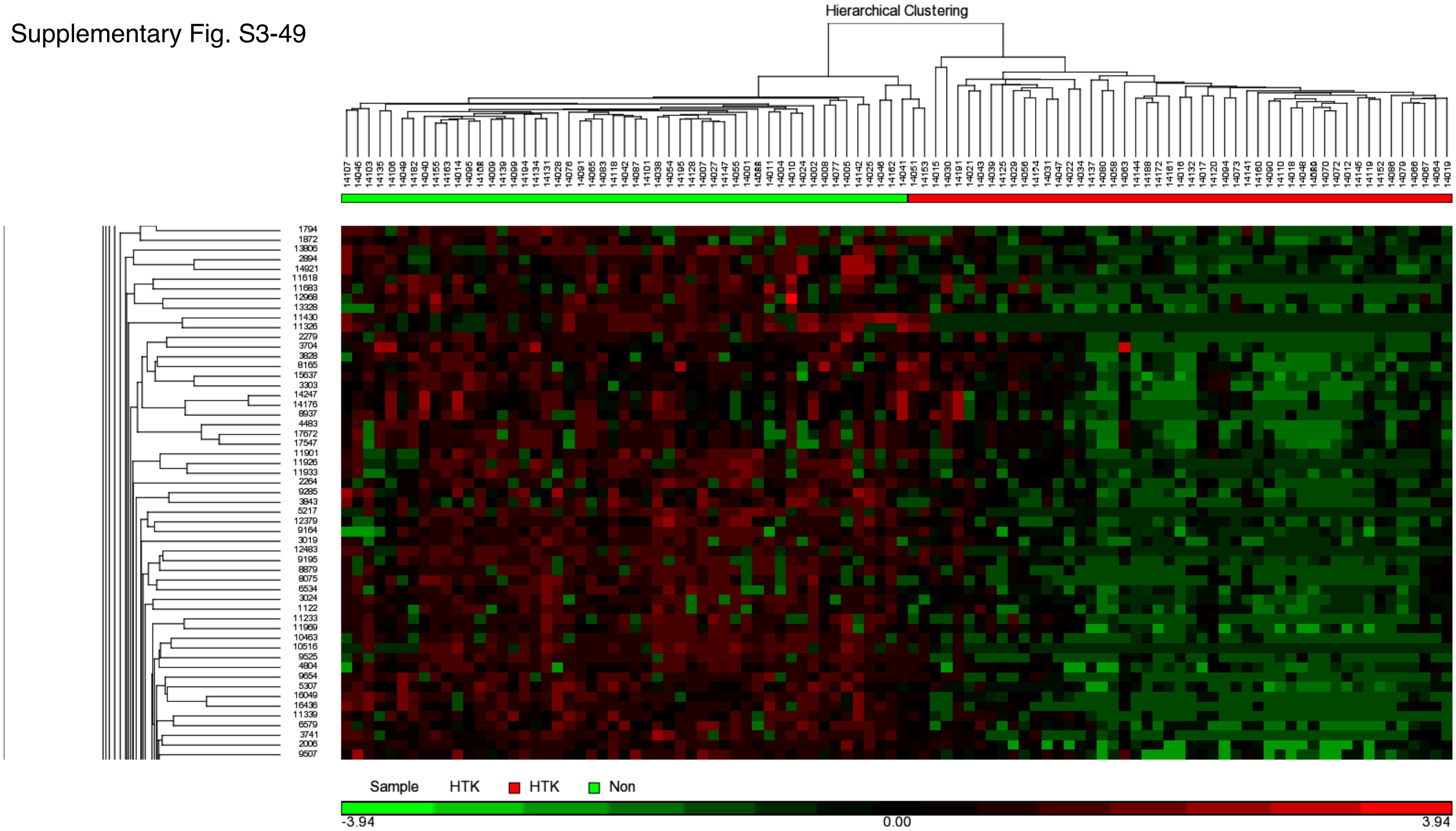

Supplementary Fig. S3-50

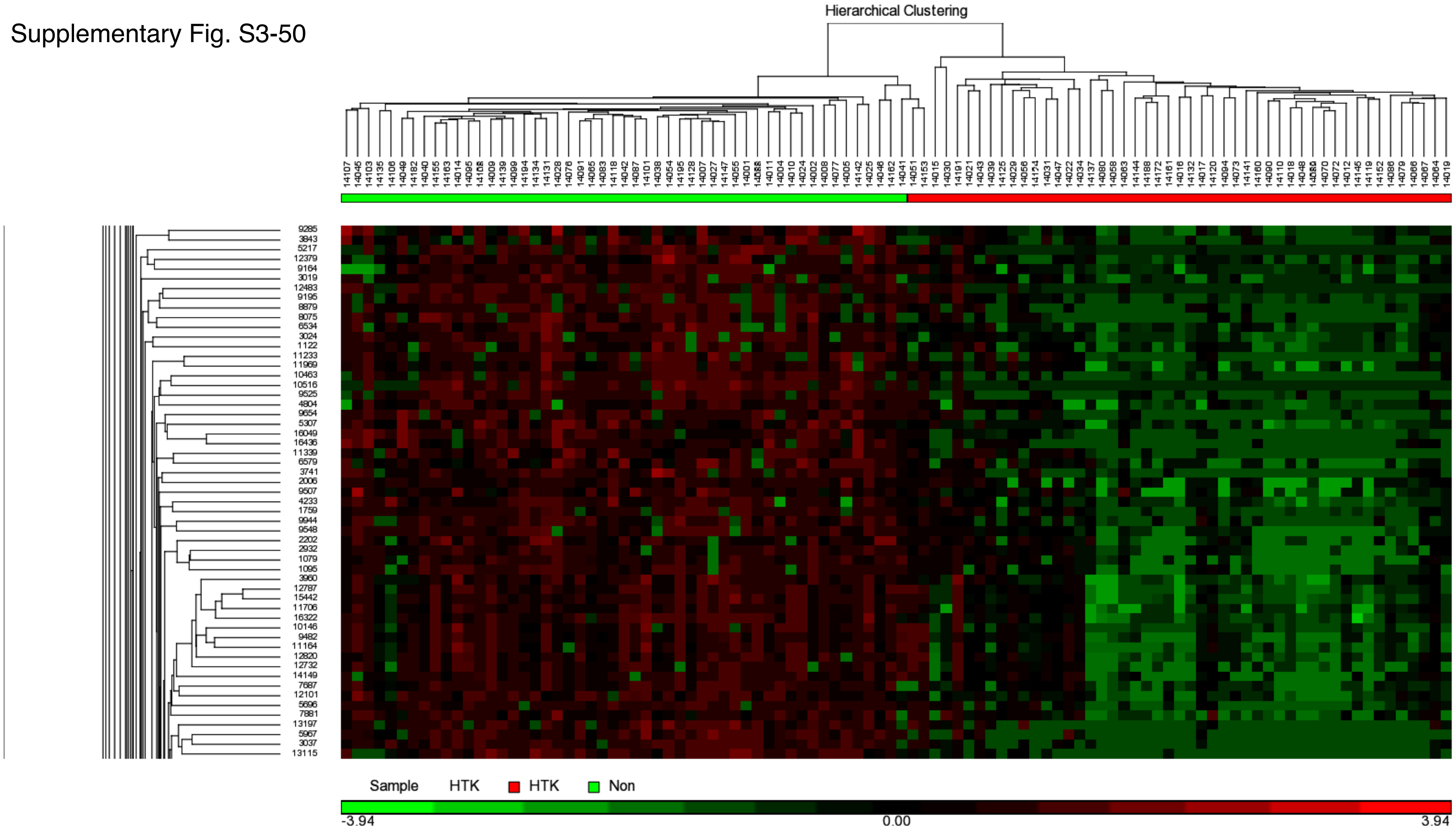

Supplementary Fig. S3-51

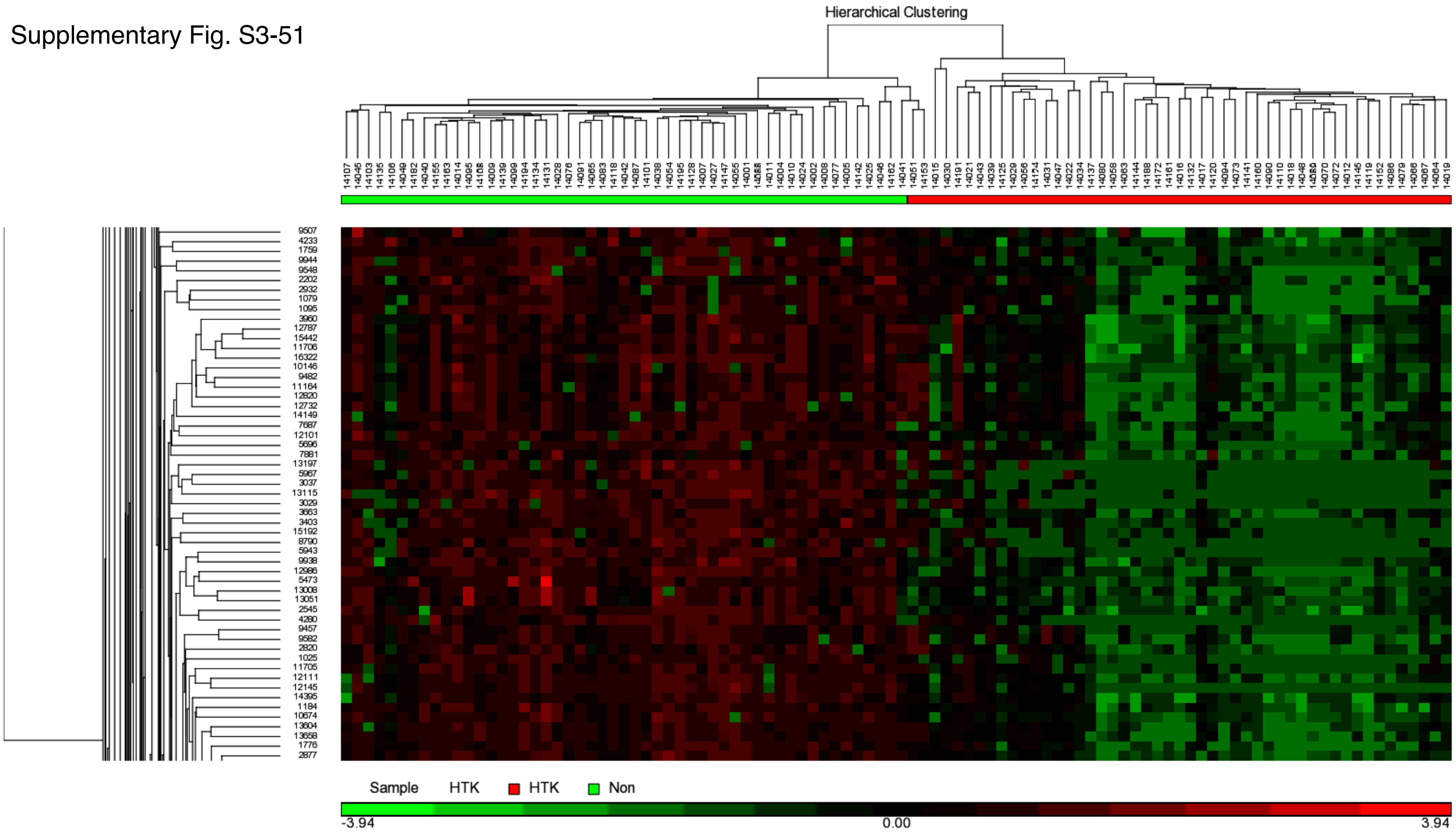

Supplementary Fig. S3-52

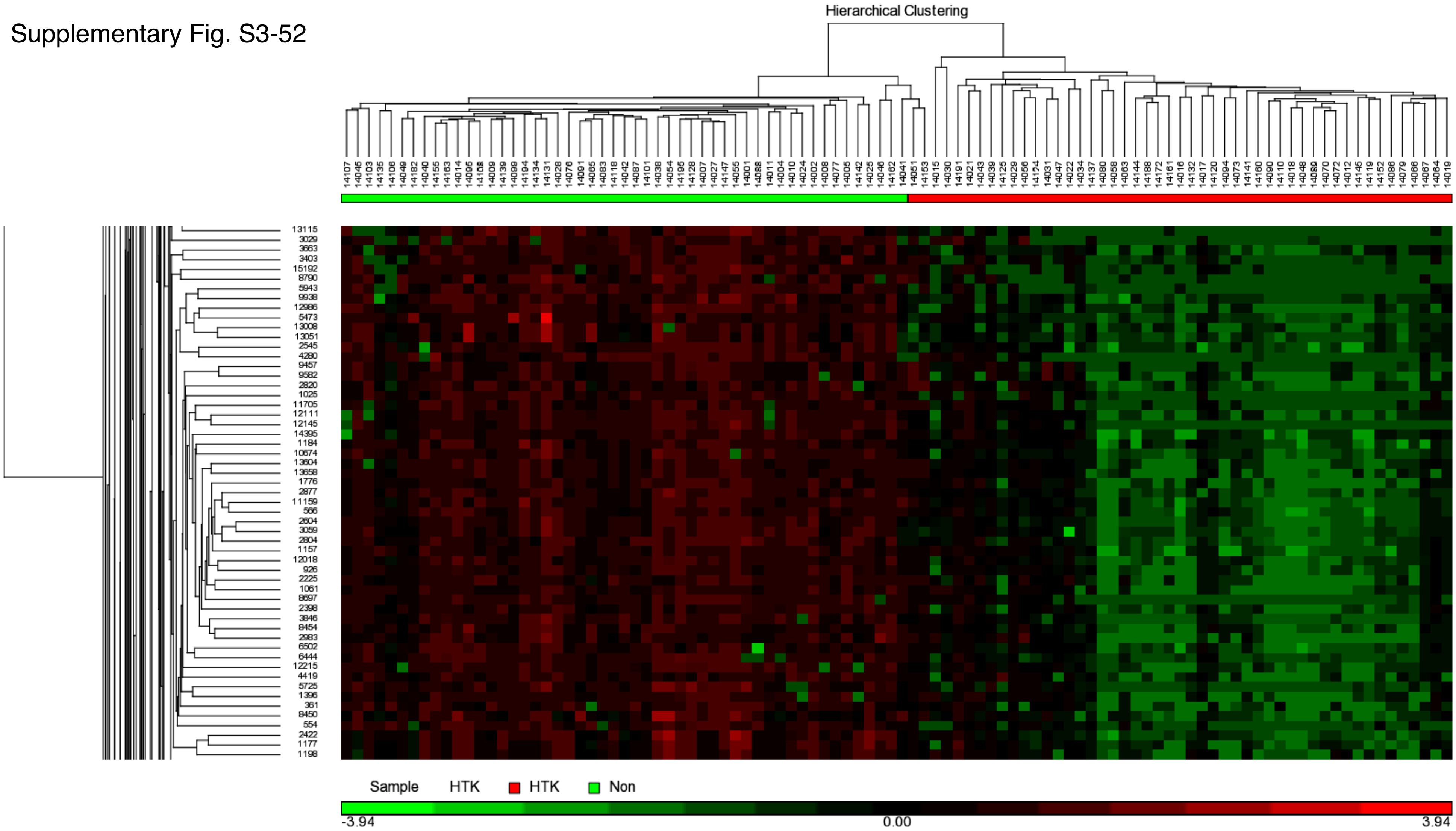

Supplementary Fig. S3-53

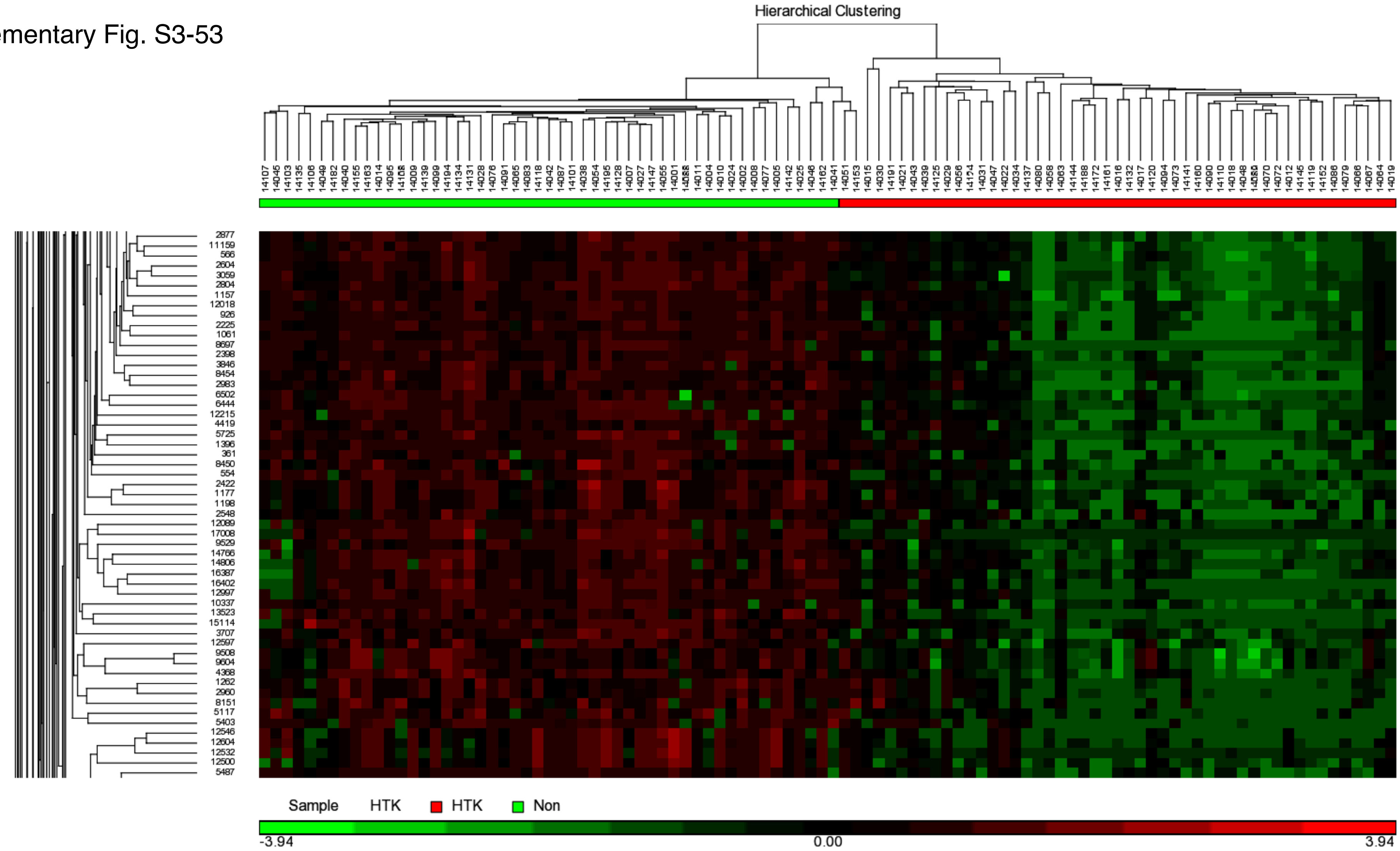

Supplementary Fig. S3-54

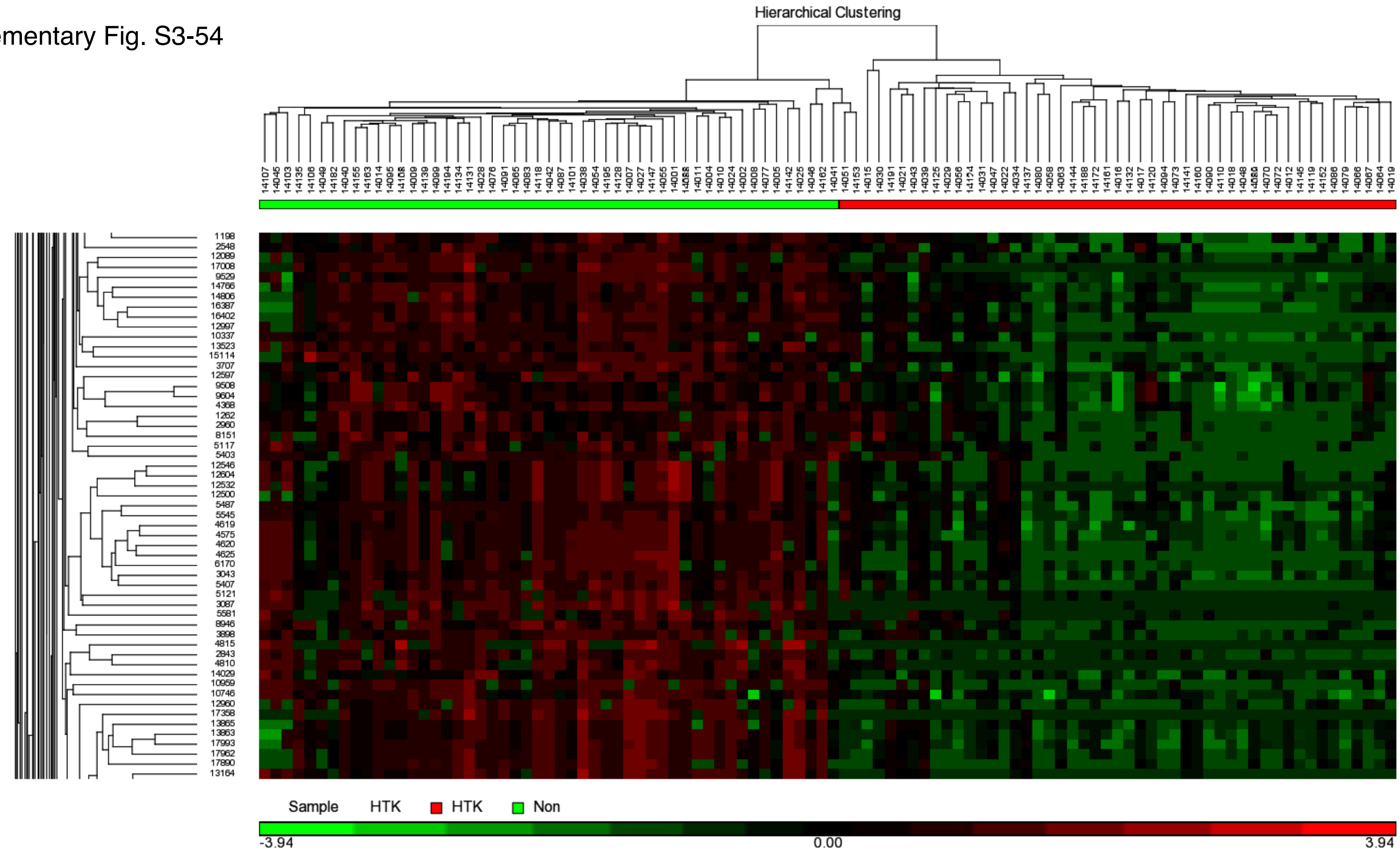

Supplementary Fig. S3-55

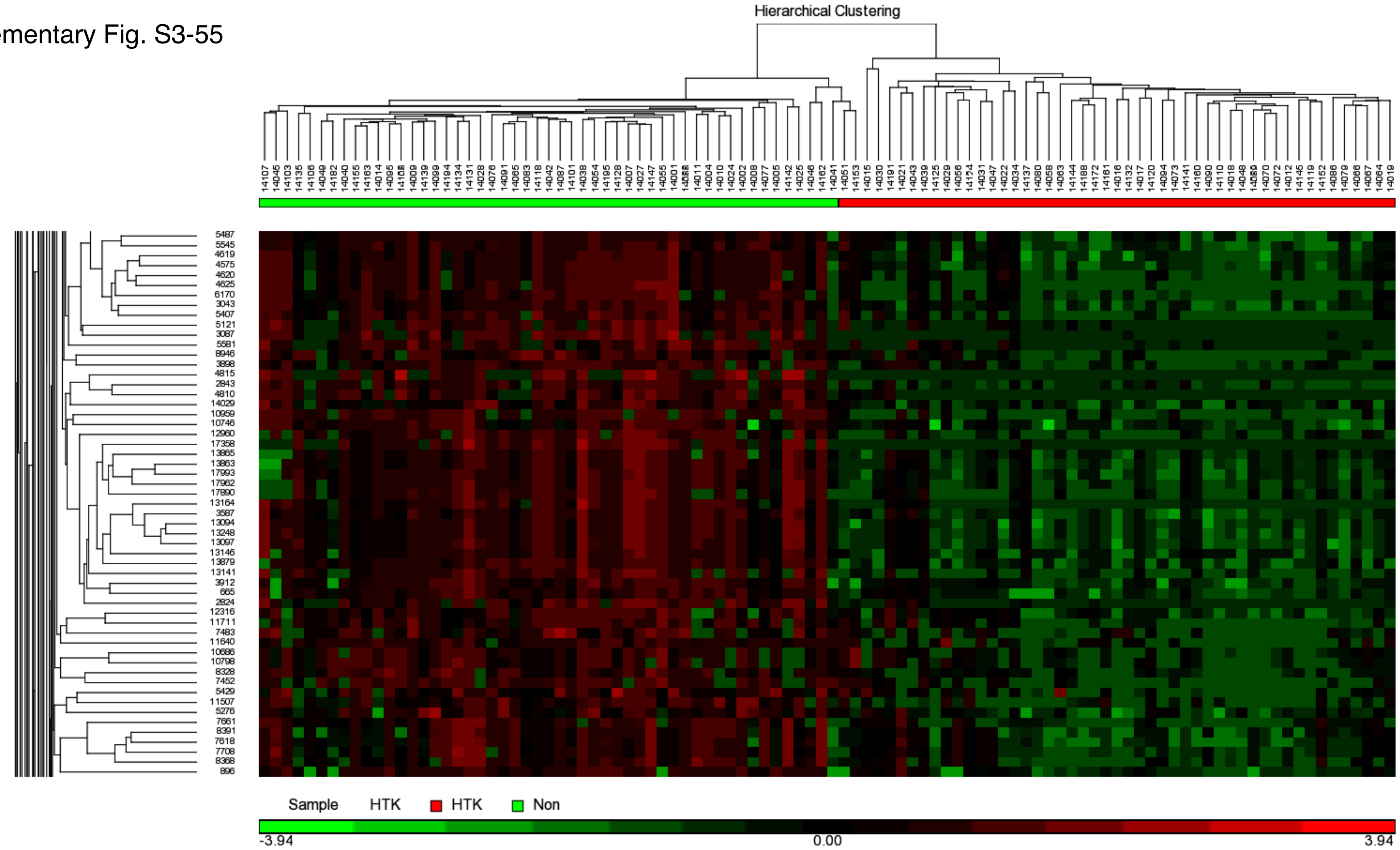

Supplementary Fig. S3-56

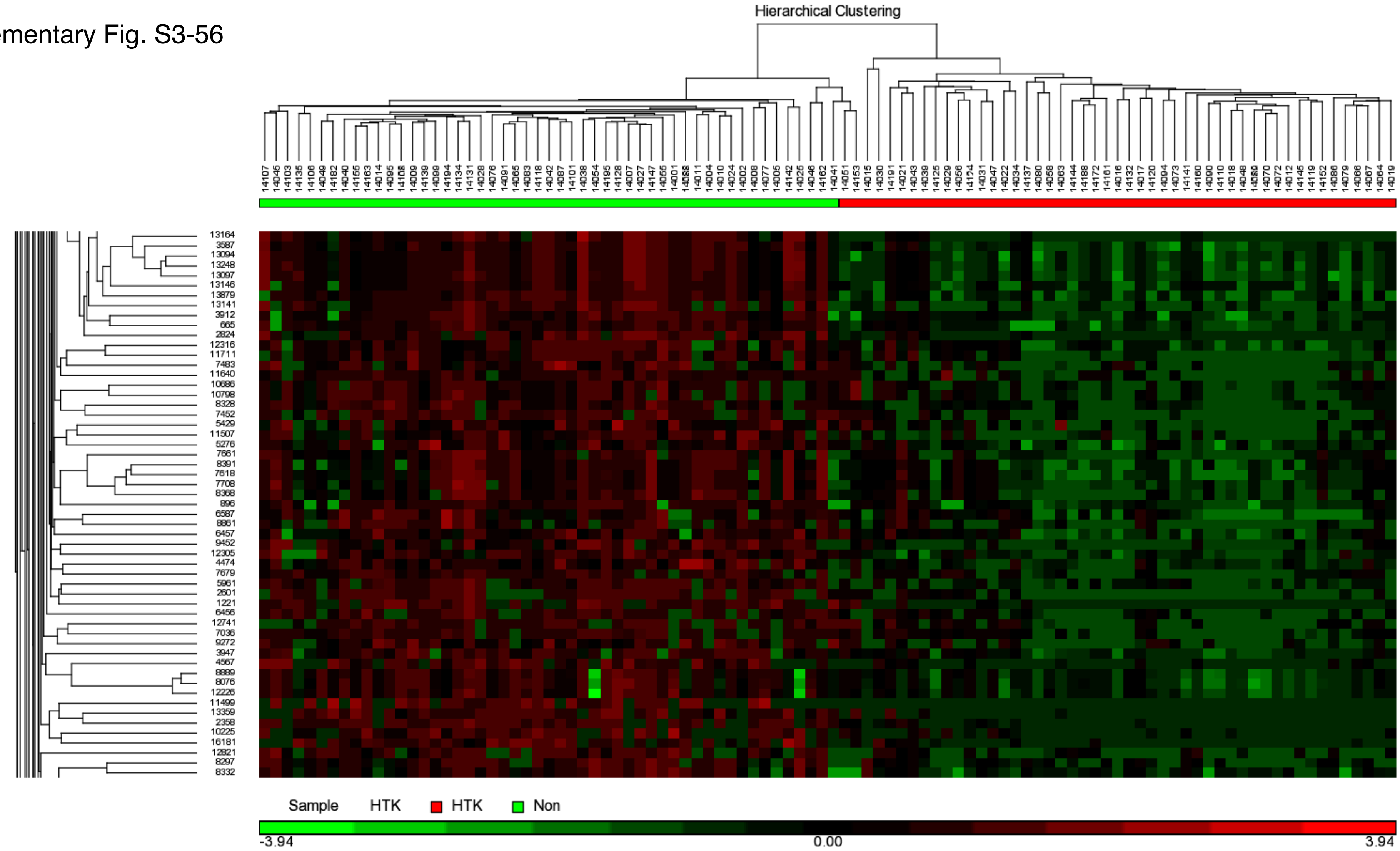

Supplementary Fig. S3-57

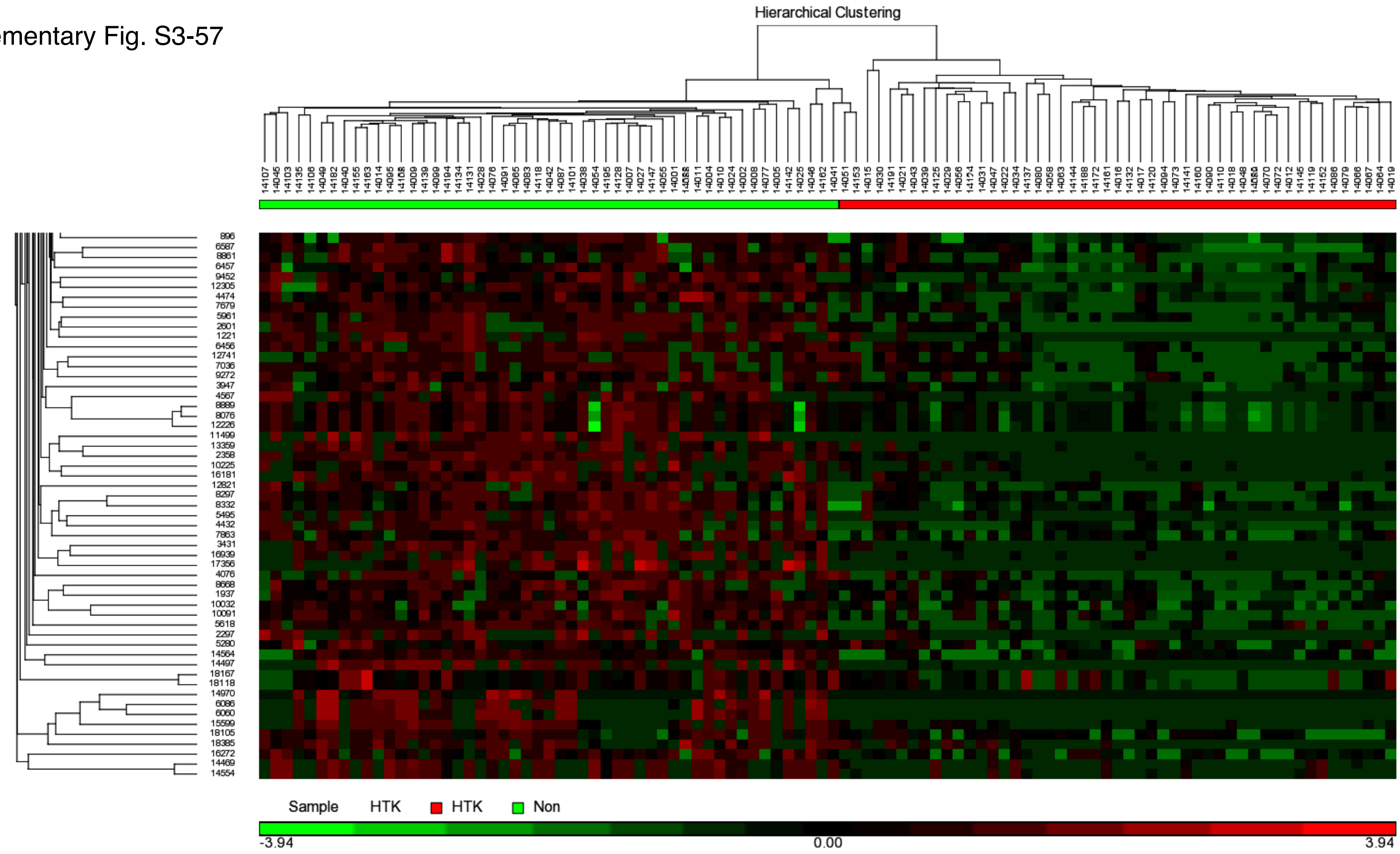

Supplement: Supplementary file 1 — Supplementary information [file 41598_2018_35631_MOESM1_ESM.pdf]
